# Differential Metabolic Stability of 4α,25- and 4β,25-Dihydroxyvitamin D_3_ and Identification of Their Metabolites

**DOI:** 10.3390/biom13071036

**Published:** 2023-06-24

**Authors:** Yuka Mizumoto, Ryota Sakamoto, Kazuto Iijima, Naoto Nakaya, Minami Odagi, Masayuki Tera, Takatsugu Hirokawa, Toshiyuki Sakaki, Kaori Yasuda, Kazuo Nagasawa

**Affiliations:** 1 Department of Biotechnology and Life Science, Faculty of Engineering, Tokyo University of Agriculture and Technology, 2-24-16 Naka-cho, Koganei 184-8588, Japan; 2 Faculty of Engineering, Toyama Prefectural University, 5180 Kurokawa, Imizu 939-0398, Japan; 3 Transborder Medical Research Center, University of Tsukuba, 1-1-1 Tennodai, Tsukuba 305-8575, Japan; 4 Division of Biomedical Science, Faculty of Medicine, University of Tsukuba, 1-1-1 Tennodai, Tsukuba 305-8575, Japan

**Keywords:** vitamin D_3_, vitamin D_3_ metabolites, 4,25-dihydroxyvitamin D_3_, 4,24,25-trihydroxyvitamin D_3_, Cytochrome P450, CYP3A4

## Abstract

Vitamin D_3_ (**1**) is metabolized by various cytochrome P450 (CYP) enzymes, resulting in the formation of diverse metabolites. Among them, 4α,25-dihydroxyvitamin D_3_ (**6a**) and 4β,25-dihydroxyvitamin D_3_ (**6b**) are both produced from 25-hydroxyvitamin D_3_ (**2**) by CYP3A4. However, **6b** is detectable in serum, whereas **6a** is not. We hypothesized that the reason for this is a difference in the susceptibility of **6a** and **6b** to CYP24A1-mediated metabolism. Here, we synthesized **6a** and **6b**, and confirmed that **6b** has greater metabolic stability than **6a**. We also identified 4α,24*R*,25- and 4β,24*R*,25-trihydroxyvitamin D_3_ (**16a** and **16b**) as metabolites of **6a** and **6b**, respectively, by HPLC comparison with synthesized authentic samples. Docking studies suggest that the β-hydroxy group at C4 contributes to the greater metabolic stability of **6b** by blocking a crucial hydrogen-bonding interaction between the C25 hydroxy group and Leu325 of CYP24A1.

## 1. Introduction

**Scheme 1:**
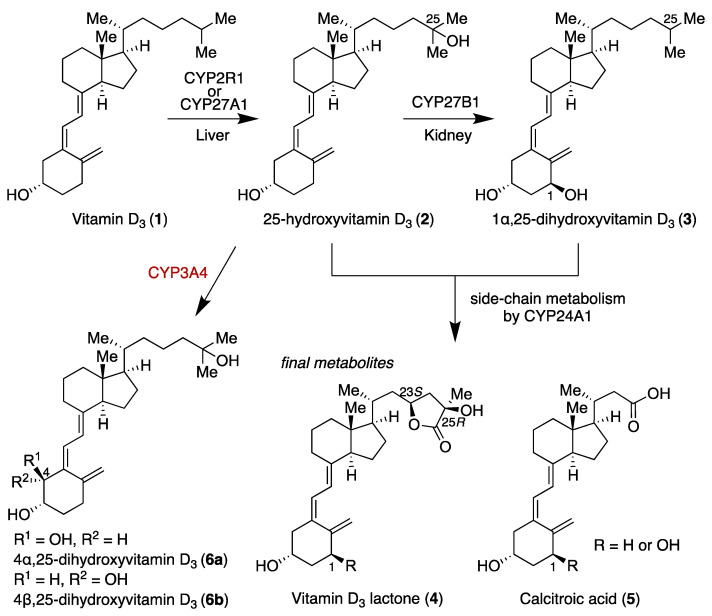
Metabolic pathways of vitamin D_3_ (**1**).

Vitamin D_3_ (**1**) is a steroid hormone that is synthesized in the skin upon exposure to sunlight or can be absorbed from foods. It is transported to the liver, where it undergoes hydroxylation at C25 by cytochrome (CYP) P450 family members CYP2R1 and CYP27A1 to form 25-hydroxyvitamin D_3_ (**2**, 25D) (Scheme 1). The resulting **2** is then transported to the kidneys, where it undergoes further hydroxylation at C1α by CYP27B1 to afford 1α,25-dihydroxyvitamin D_3_ (**3**, 1,25D) [1], which regulates multiple physiological functions, including calcium homeostasis [2], bone metabolism [3], cell differentiation [4], and immune regulation [5] by binding to the vitamin D receptor (VDR), which is a nuclear receptor. Subsequently, both **2** and **3** undergo metabolism of their side chains. Specifically, CYP24A1 mediates hydroxylation at the C23*S* or C24*R* position of **2** and **3**, leading to the formation of vitamin D_3_ lactone (**4**) and calcitroic acid (**5**), respectively, as final metabolites [6,7,8,9,10,11].

In 2011, Thummel et al. found a novel vitamin D metabolite, 4β,25-dihydroxyvitamin D_3_ (**6b**), which is formed from **2** by hydroxylation at C4β [12]. The conversion of compound **2** to compound **6** may be involved in drug-induced osteomalacia. Notably, in vitro studies revealed that CYP3A4 generated 4α,25-dihydroxyvitamin D_3_ (**6a**) and **6b** at a ratio of 1:2. Interestingly, however, analysis of human serum showed the presence of only **6b**, with no detection of **6a**. This intriguing result led us to propose that differential metabolic stability between **6a** and **6b** might explain this discrepancy. We hypothesized that a difference in metabolic stability between **6a** and **6b** would account for this finding. Here, we synthesized **6a** and **6b**, and evaluated their metabolic stability in the presence of CYP24A1, which plays a major role in vitamin D metabolism. We also identified metabolites of **6** generated by CYP24A1 by means of HPLC comparison with synthesized authentic samples.

## 2. Materials and Methods

### 2.1. Synthesis of ***6a*** and ***6b***

All reagents were supplied by commercial sources without further purification. All reactions involving air- or moisture-sensitive reagents were carried out in flame-dried glassware under argon atmosphere. Flash chromatography was performed using silica gel 60 (spherical, particle size 0.040−0.100 mm; Kanto Co., Inc., Tokyo, Japan), and preparative TLC (PLC) was performed using PLC silica gel 60 F254 (0.5 mm, Merck Ltd., Darmstadt, Germany). Optical rotations were measured on a JASCO P-2200 polarimeter (JASCO Co., Inc., Tokyo, Japan). ^1^H and ^13^C NMR spectra were recorded on JNM-AL300 (300 MHz, JEOL Ltd., Tokyo, Japan), JNM-ECX 400 (400 MHz, JEOL Ltd., Tokyo, Japan), and JNM-ECA 500 (500 MHz, JEOL Ltd., Tokyo, Japan) spectrometers. Chemical shift in CDCl_3_ was reported in the scale relative to CDCl_3_ (7.26 ppm) for ^1^H NMR. For ^13^C NMR, the chemical shift was reported in the scale relative to CDCl_3_ (77.0 ppm) and CD_3_OD (49.0 ppm) as an internal reference. HRMS (ESI) measurements were performed on a JMS-T100LC spectrometer (JEOL Ltd., Tokyo, Japan).

(*S*)-3,4-bis((*tert*-butyldimethylsilyl)oxy)butyl benzoate (**S1**): To a solution of diol **9** [13] (300 mg, 1.4 mmol) in CH_2_Cl_2_ (7 mL) was added 2,6-lutidine (0.44 mL, 3.7 mmol) and *tert*-butyldimethylsilyl triflate (0.82 mL, 3.6 mmol) at 0 °C. The resulting mixture was allowed to warm to room temperature and stirred for 30 min. The reaction mixture was quenched with H_2_O, and the aqueous layer was extracted with CH_2_Cl_2_ three times. The combined organic layer was washed with brine, dried over MgSO_4_, filtered, and concentrated in vacuo. The residue was purified by flash chromatography on silica gel (*n*-hexane/ethyl acetate = 20:1) to give the protected product **S1** (627 mg, 99%) as a colorless oil. [α]${}_{D}^{25}$ = –10.6 (*c* 0.87, CHCl_3_); ^1^H NMR (300 MHz, CDCl_3_) δ 8.05 (d, *J* = 7.9 Hz, 2H), 7.55 (t, *J* = 7.7 Hz, 1H), 7.44 (t, *J* = 7.7 Hz, 2H), 4.34–4.51 (m, 2H), 3.86–3.94 (m, 1H), 3.62 (q, *J* = 4.9 Hz, 1H), 3.48 (q, *J* = 5.5 Hz, 1H), 2.05–2.15 (m, 1H), 1.81 (td, *J* = 13.6, 5.6 Hz, 1H), 0.89 (s, 18H), 0.07 (d, *J* = 4.8 Hz, 12H); ^13^C NMR (75 MHz, CDCl_3_) δ 166.5, 132.8, 130.4, 129.5, 128.3, 70.1, 67.5, 61.8, 33.4, 31.1, 25.9, 25.8, 25.7, −3.0, −4.3, −5.0, −5.4; HRMS (ESI) *m*/*z*: [M + Na]^+^ calcd for C_23_H_42_O_4_Si_2_Na 461.2519, found 461.2493.

(*S*)-3-((*tert*-butyldimethylsilyl)oxy)-4-hydroxybutyl benzoate (**10**): To a solution of diol **S1** (627 mg, 1.4 mmol) in MeOH/THF = 1:1 (17 mL) was added (±)-CSA (40 mg, 0.17 mmol) at room temperature. The resulting mixture was stirred at the same temperature for 1 h. The reaction mixture was quenched with sat. NaHCO_3_ aq, and the aqueous layer was extracted with CH_2_Cl_2_ three times. The combined organic layer was washed with brine, dried over MgSO_4_, filtered, and concentrated in vacuo. The residue was purified by flash chromatography on silica gel (*n*-hexane/ethyl acetate = 10:1) to give **10** (77 mg, 54%) as a colorless oil. [α]${}_{D}^{25}$ = –9.8 (*c* 0.53, CHCl_3_); ^1^H NMR (300 MHz, CDCl_3_) δ 8.03 (d, *J* = 7.2 Hz, 2H), 7.56 (t, *J* = 7.4 Hz, 1H), 7.44 (t, *J* = 7.7 Hz, 2H), 4.31–4.49 (m, 2H), 3.96–4.03 (m, 1H), 3.66 (q, *J* = 4.9 Hz, 1H), 3.55 (q, *J* = 5.3 Hz, 1H), 1.99 (q, *J* = 6.3 Hz, 2H), 0.91 (s, 9H), 0.10 (d, *J* = 2.1 Hz, 6H); ^13^C NMR (75 MHz, CDCl_3_) δ 166.5, 132.9, 130.2, 129.5, 128.4, 69.7, 66.4, 61.6, 32.9, 25.8, 18.0, −4.6, −4.8; HRMS (ESI) *m*/*z*: [M + Na]^+^ calcd for C_17_H_28_O_4_SiNa 347.1655, found 347.1670.

(3*S*,4*R*)-3-((*tert*-butyldimethylsilyl)oxy)-4-hydroxy-6-(trimethylsilyl)hex-5-yn-1-yl benzoate (**11b**): To a solution of oxalic acid dichloride (0.48 mL, 5.6 mmol) in CH_2_Cl_2_ (7 mL) was added dimethyl sulfoxide (1.0 mL, 14.0 mmol) at –78 °C. The resulting mixture was stirred at the same temperature for 10 min, then a solution of **10** in CH_2_Cl_2_ (0.4 M, 7 mL, 2.78 mmol) and triethylamine (3.9 mL, 27.8 mmol) were added dropwise at the same temperature. The resulting mixture was allowed to warm to room temperature and stirred for 1 h. The reaction mixture was quenched with H_2_O, and the aqueous layer was extracted with CH_2_Cl_2_ three times. The combined organic layer was washed with brine, dried over MgSO_4_, filtered, and concentrated in vacuo. The residue was purified by flash chromatography on silica gel (*n*-hexane/ethyl acetate = 1:1) to give the aldehyde as a yellow oil. The crude residue was used in the subsequent step without purification. To a solution of trimethylsilylacetylene (1.0 mL, 7.2 mmol) in THF (12 mL) was added *n*-butyllithium (2.6 M in hexane; 2.3 mL, 6.0 mmol) at –78 °C. The resulting mixture was stirred at the same temperature for 30 min, then a solution of the aldehyde in THF (0.3 M, 9.3 mL, 2.78 mmol) was added dropwise at the same temperature. The resulting mixture was stirred at the same temperature for 15 min. The reaction mixture was quenched with sat. NH_4_Cl aq, and the aqueous layer was extracted with ethyl acetate three times. The combined organic layer was washed with brine, dried over MgSO_4_, filtered, and concentrated in vacuo. The residue was purified by flash chromatography on silica gel (*n*-hexane/ethyl acetate = 30:1) to give **11b** (910 mg, 90%) as a colorless oil. [α]${}_{D}^{25}$ = 1.5 (*c* 5.36, CHCl_3_); ^1^H NMR (300 MHz, CDCl_3_) δ 8.04 (d, *J* = 6.9 Hz, 2H), 7.56 (t, *J* = 7.4 Hz, 1H), 7.44 (t, *J* = 7.6 Hz, 2H), 4.48–4.56 (m, 1H), 4.30–4.42 (m, 2H), 3.99–4.04 (m, 1H), 2.16–2.27 (m, 1H), 1.97–2.08 (m, 1H), 0.91 (s, 9H), 0.16 (s, 9H), 0.10 (d, *J* = 5.2 Hz, 6H); ^13^C NMR (75 MHz, CDCl_3_) δ 166.4, 132.9, 130.2, 129.5, 128.3, 103.0, 91.7, 71.9, 66.5, 61.6, 31.1, 25.7, 18.0, −0.3, −4.5, −4.7; HRMS (ESI) *m*/*z*: [M + Na]^+^ calcd for C_22_H_36_O_4_Si_2_Na 443.2050, found 443.2017.

(*S*)-3-((*tert*-butyldimethylsilyl)oxy)-4-oxo-6-(trimethylsilyl)hex-5-yn-1-yl benzoate (**S2**): To a solution of **11b** (100 mg, 0.24 mmol) and pyridine (0.38 mL) in CH_2_Cl_2_ (19 mL) was added Dess–Martin periodinane (404 mg, 0.95 mmol) at 0 °C. The resulting mixture was allowed to warm to room temperature and stirred for 30 min. The reaction mixture was quenched with sat. Na_2_S_2_O_3_ aq, and the aqueous layer was extracted with CH_2_Cl_2_ three times. The combined organic layer was washed with brine, dried over MgSO_4_, filtered, and concentrated in vacuo. The residue was purified by flash chromatography on silica gel (*n*-hexane/ethyl acetate = 10:1) to give **S2** (94 mg, 94%) as a colorless oil. [α]${}_{D}^{25}$ = +0.7 (*c* 1.37, CHCl_3_); ^1^H NMR (300 MHz, CDCl_3_) δ 8.02 (d, *J* = 6.9 Hz, 2H), 7.54 (t, *J* = 7.4 Hz, 1H), 7.43 (t, *J* = 7.4 Hz, 2H), 4.46–4.56 (m, 1H), 4.32–4.43 (m, 2H), 2.18–2.26 (m, 2H), 0.93 (s, 9H), 0.05–0.22 (m, 15H); ^13^C NMR (75 MHz, CDCl_3_) δ 189.5, 166.3, 132.9, 130.0, 129.6, 128.3, 102.4, 100.4, 75.5, 60.1, 33.7, 31.1, 25.7, 18.1, −1.0, −4.7, −5.4; HRMS (ESI) *m*/*z*: [M + Na]+ calcd for C_22_H_34_O_4_Si_2_Na 441.1893, found 441.1877.

(3*S*,4*S*)-3-((*tert*-butyldimethylsilyl)oxy)-4-hydroxy-6-(trimethylsilyl)hex-5-yn-1-yl benzoate (**11a**): To a solution of **S2** (94 mg, 0.22 mmol) in THF (11 mL) was added L-selectride (1.0 M in THF, 0.40 mL, 0.40 mmol) at –78 °C. The resulting mixture was stirred at the same temperature for 1 h. The reaction mixture was quenched with sat. NH_4_Cl aq, and the aqueous layer was extracted with ethyl acetate three times. The combined organic layer was washed with brine, dried over MgSO_4_, filtered, and concentrated in vacuo. The residue was purified by flash chromatography on silica gel (*n*-hexane/ethyl acetate = 10:1) to give **11a** (94 mg, 99%) as a colorless oil. [α]${}_{D}^{25}$ = –9.1 (*c* 0.35, CHCl_3_); ^1^H NMR (300 MHz, CDCl_3_) δ 8.05 (d, *J* = 6.9 Hz, 2H), 7.56 (t, *J* = 7.4 Hz, 1H), 7.44 (t, *J* = 7.6 Hz, 2H), 4.32–4.51 (m, 3H), 3.99 (q, *J* = 5.4 Hz, 1H), 1.99–2.19 (m, 2H), 0.92 (s, 9H), 0.12–0.16 (m, 15H); ^13^C NMR (75 MHz, CDCl_3_) δ 166.4, 132.9, 130.1, 129.5, 128.3, 104.6, 90.6, 72.5, 65.8, 61.2, 32.7, 25.9, 18.1, −0.3, −4.3, −4.6; HRMS (ESI) *m*/*z*: [M + Na]+ calcd for C_22_H_36_O_4_Si_2_Na 443.2050, found 443.2054.

(3*S*,4*S*)-3,4-bis((*tert*-butyldimethylsilyl)oxy)-6-(trimethylsilyl)hex-5-yn-1-yl benzoate (**S3a**): To a solution of **11a** (115 mg, 0.27 mmol) in CH_2_Cl_2_ (3 mL) was added 2,6-lutidine (98 μL, 0.82 mmol) and *tert*-butyldimethylsilyl triflate (94 μL, 0.41 mmol) at 0 °C. The resulting mixture was allowed to warm to room temperature and stirred for 30 min. The reaction mixture was quenched with H_2_O, and the aqueous layer was extracted with CH_2_Cl_2_ three times. The combined organic layer was washed with brine, dried over MgSO_4_, filtered, and concentrated in vacuo. The residue was purified by flash chromatography on silica gel (*n*-hexane/ethyl acetate = 40:1) to give **S3a** (143 mg, 98%) as a colorless oil. [α]${}_{D}^{25}$ = –5.6 (*c* 0.25, CHCl_3_); ^1^H NMR (300 MHz, CDCl_3_) δ 8.05 (d, *J* = 7.2 Hz, 2H), 7.55 (t, *J* = 7.2 Hz, 1H), 7.43 (t, *J* = 7.6 Hz, 2H), 4.35–4.58 (m, 3H), 3.81–3.87 (m, 1H), 2.14–2.25 (m, 1H), 1.95–2.09 (m, 1H), 0.90 (d, *J* = 1.4 Hz, 18H), 0.05–0.16 (m, 21H); ^13^C NMR (75 MHz, CDCl_3_) δ 166.5, 132.8, 130.5, 129.5, 128.3, 104.8, 90.5, 71.5, 67.3, 61.8, 31.2, 25.8, 25.8, 18.2, 18.0, −0.3, −4.5, −4.6, −4.8, −4.9; HRMS (ESI) *m*/*z*: [M + Na]+ calcd for C_28_H_50_O_4_Si_3_Na 557.2915, found 557.2909.

(3*S*,4*R*)-3,4-bis((*tert*-butyldimethylsilyl)oxy)-6-(trimethylsilyl)hex-5-yn-1-yl benzoate (**S3b**): To a solution of **11b** (264 mg, 0.63 mmol) in CH_2_Cl_2_ (6 mL) was added 2,6-lutidine (0.22 mL, 1.9 mmol) and *tert*-butyldimethylsilyl triflate (0.22 mL, 0.94 mmol) at 0 °C. The resulting mixture was allowed to warm to room temperature and stirred for 30 min. The reaction mixture was quenched with H_2_O, and the aqueous layer was extracted with CH_2_Cl_2_ three times. The combined organic layer was washed with brine, dried over MgSO_4_, filtered, and concentrated in vacuo. The residue was purified by flash chromatography on silica gel (*n*-hexane/ethyl acetate = 40:1) to give **S3b** (334 mg, 99%) as a colorless oil. [α]${}_{D}^{25}$ = –26.2 (*c* 1.38, CHCl_3_); ^1^H NMR (300 MHz, CDCl_3_) δ 8.05 (d, *J* = 7.2 Hz, 2H), 7.56 (t, *J* = 7.4 Hz, 1H), 7.44 (t, *J* = 7.4 Hz, 2H), 4.46–4.54 (m, 1H), 4.36–4.42 (m, 1H), 4.28 (d, *J* = 4.5 Hz, 1H), 3.89–3.94 (m, 1H), 2.08–2.19 (m, 1H), 1.92–2.05 (m, 1H), 0.91 (d, *J* = 1.4 Hz, 18H), 0.05–0.15 (m, 21H); ^13^C NMR (75 MHz, CDCl_3_) δ 166.5, 132.8, 130.5, 129.5, 128.3, 105.8, 90.3, 72.9, 67.8, 61.8, 32.0, 25.9, 25.8, 18.1, −0.3, −4.1, −4.4, −4.8, −5.0; HRMS (ESI) *m*/*z*: [M + Na]+ calcd for C_28_H_50_O_4_Si_3_Na 557.2915, found 557.2872.

(3*S*,4*S*)-3,4-bis((*tert*-butyldimethylsilyl)oxy)hex-5-yn-1-ol (**12a**): To a solution of **S3a** (143 mg, 0.27 mmol) in MeOH (0.9 mL) was added K_2_CO_3_ (148 mg, 1.1 mmol) at room temperature. The resulting mixture was stirred at the same temperature for 1 h. The reaction mixture was quenched with H_2_O, and the aqueous layer was extracted with ethyl acetate three times. The combined organic layer was washed with brine, dried over MgSO_4_, filtered, and concentrated in vacuo. The residue was purified by flash chromatography on silica gel (*n*-hexane/ethyl acetate = 30:1) to give **12a** (68 mg, 71%) as a colorless oil. [α]${}_{D}^{25}$ = –14.5 (*c* 0.29, CHCl_3_); ^1^H NMR (300 MHz, CDCl_3_) δ 4.38 (q, *J* = 2.3 Hz, 1H), 3.79 (tt, *J* = 8.1, 2.9 Hz, 3H), 2.38 (d, *J* = 2.1 Hz, 1H), 1.84–2.09 (m, 2H), 0.90 (d, *J* = 1.4 Hz, 18H), 0.09–0.15 (m, 12H); ^13^C NMR (75 MHz, CDCl_3_) δ 82.4, 74.0, 73.2, 66.9, 60.1, 34.8, 25.7, 18.1, 18.0, −4.6, −4.7, −4.8, −5.1; HRMS (ESI) *m*/*z*: [M + Na]^+^ calcd for C_18_H_38_O_3_Si_2_Na 381.2257, found 338.2297.

(3*S*,4*R*)-3,4-bis((*tert*-butyldimethylsilyl)oxy)hex-5-yn-1-ol (**12b**): To a solution of **S3b** (206 mg, 0.39 mmol) in MeOH (1.3 mL) was added K_2_CO_3_ (213 mg, 1.5 mmol) at room temperature. The resulting mixture was stirred at the same temperature for 1 h. The reaction mixture was quenched with H_2_O, and the aqueous layer was extracted with ethyl acetate three times. The combined organic layer was washed with brine, dried over MgSO_4_, filtered, and concentrated in vacuo. The residue was purified by flash chromatography on silica gel (*n*-hexane/ethyl acetate = 30:1) to give **12b** (104 mg, 76%) as a colorless oil. [α]${}_{D}^{25}$ = –22.3 (*c* 1.60, CHCl_3_); ^1^H NMR (300 MHz, CDCl_3_) δ 4.36 (q, *J* = 2.2 Hz, 1H), 3.94 (q, *J* = 4.8 Hz, 1H), 3.78 (td, *J* = 5.8, 2.3 Hz, 2H), 2.41 (d, *J* = 2.1 Hz, 1H), 1.82–2.06 (m, 2H), 0.90 (d, *J* = 1.7 Hz, 18H), 0.09–0.17 (m, 12H); ^13^C NMR (75 MHz, CDCl_3_) δ 83.5, 74.1, 66.9, 59.0, 34.6, 25.9, 25.7, 18.2, 18.1, −4.3, −4.6, −4.8, −5.1; HRMS (ESI) *m*/*z*: [M + Na]^+^ calcd for C_18_H_38_O_3_Si_2_Na 381.2257, found 338.2263.

(5*S*,6*S*)-5-ethynyl-6-(2-iodoethyl)-2,2,3,3,8,8,9,9-octamethyl-4,7-dioxa-3,8-disiladecane (**S4a**): To a solution of **12a** (49 mg, 0.38 mmol) in THF (0.6 mL) was added triphenylphosphine (70 mg, 0.33 mmol), imidazole (28 mg, 0.41 mmol), and iodine (112 mg, 0.44 mmol) at –20 °C. The resulting mixture was stirred at the same temperature for 30 min. The resulting mixture was allowed to warm to room temperature and stirred for 20 min. The reaction mixture was quenched with sat. Na_2_S_2_O_3_ aq, and the aqueous layer was extracted with ethyl acetate three times. The combined organic layer was washed with brine, dried over MgSO_4_, filtered, and concentrated in vacuo. The residue was purified by flash chromatography on silica gel (*n*-hexane) to give **S4a** (54 mg, 83%) as a yellow oil. [α]${}_{D}^{25}$ = –35.2 (*c* 0.25, CHCl_3_); ^1^H NMR (300 MHz, CDCl_3_) δ 4.37 (q, *J* = 2.3 Hz, 1H), 3.68–3.74 (m, 1H), 3.38 (qd, *J* = 5.4, 3.8 Hz, 1H), 3.22 (td, *J* = 9.5, 6.3 Hz, 1H), 2.33 (d, *J* = 2.1 Hz, 1H), 2.21–2.30 (m, 1H), 2.04–2.15 (m, 1H), 0.90 (d, *J* = 0.7 Hz, 18H), 0.11–0.14 (m, 12H); ^13^C NMR (75 MHz, CDCl_3_) δ 82.6, 73.9, 73.5, 66.3, 35.4, 31.1, 25.7, 18.1, 18.0, 4.2, −4.3, −4.5, −4.7, −5.1; HRMS (ESI) *m*/*z*: [M + Na]^+^ calcd for C_18_H_37_IO_2_Si_2_Na 491.1274, found 491.1255.

(5*R*,6*S*)-5-ethynyl-6-(2-iodoethyl)-2,2,3,3,8,8,9,9-octamethyl-4,7-dioxa-3,8-disiladecane (**S4b**): To a solution of **12b** (136 mg, 0.38 mmol) in THF (1.6 mL) was added triphenylphosphine (193 mg, 0.91 mmol), imidazole (77.2 mg, 1.1 mmol), and iodine (308 mg, 1.2 mmol) at –20 °C. The resulting mixture was stirred at the same temperature for 30 min. The resulting mixture was allowed to warm to room temperature and stirred for 20 min. The reaction mixture was quenched with sat. Na_2_S_2_O_3_ aq, and the aqueous layer was extracted with ethyl acetate three times. The combined organic layer was washed with brine, dried over MgSO_4_, filtered, and concentrated in vacuo. The residue was purified by flash chromatography on silica gel (*n*-hexane) to give **S4b** (149 mg, 84%) as a yellow oil. [α]${}_{D}^{25}$ = –28.3 (*c* 1.54, CHCl_3_); ^1^H NMR (300 MHz, CDCl_3_) δ 4.26 (q, *J* = 2.1 Hz, 1H), 3.77 (td, *J* = 5.3, 3.3 Hz, 1H), 3.19–3.35 (m, 2H), 2.39 (d, *J* = 2.4 Hz, 1H), 2.11–2.20 (m, 2H), 0.90 (d, *J* = 2.1 Hz, 18H), 0.12–0.15 (m, 12H); ^13^C NMR (75 MHz, CDCl_3_) δ 83.3, 75.9, 74.0, 66.8, 37.1, 25.9, 25.8, 3.1, −4.1, −4.5, −5.2; HRMS (ESI) *m*/*z*: [M + Na]^+^ calcd for C_18_H_37_IO_2_Si_2_Na 491.1274, found 491.1243.

(4*S*,5*R*)-4,5-bis((*tert*-butyldimethylsilyl)oxy)hept-6-ynenitrile (**13a**). To a solution of **S4a** (54 mg, 0.11 mmol) in DMF (0.3 mL) was added sodium cyanide (8.4 mg, 0.17 mmol) at room temperature. The resulting mixture was allowed to warm to 90 °C and stirred for 20 min. The reaction mixture was quenched with sat. NaHCO_3_ aq, and the aqueous layer was extracted with ethyl acetate three times. The combined organic layer was washed with brine, dried over MgSO_4_, filtered, and concentrated in vacuo. The residue was purified by flash chromatography on silica gel (*n*-hexane/ethyl acetate = 80:1) to give **13a** (42 mg, 99%) as a yellow oil. [α]${}_{D}^{25}$ = –27.6 (*c* 0.29, CHCl_3_); ^1^H NMR (300 MHz, CDCl_3_) δ 4.38 (q, *J* = 2.3 Hz, 1H), 3.67–3.73 (m, 1H), 2.39–2.58 (m, 2H), 2.36 (d, *J* = 2.1 Hz, 1H), 2.04–2.16 (m, 1H), 1.90–2.02 (m, 1H), 0.90 (s, 18H), 0.11–0.14 (m, 12H); ^13^C NMR (75 MHz, CDCl_3_) δ 119.9, 82.0, 74.0, 72.3, 66.4, 31.1, 27.6, 25.7, 18.1, 18.0, −4.6, −4.8, −5.1; HRMS (ESI) *m*/*z*: [M + Na]^+^ calcd for C_19_H_37_NO_2_Si_2_Na 390.2261, found 390.2290.

(4*S*,5*R*)-4,5-bis((*tert*-butyldimethylsilyl)oxy)hept-6-ynenitrile (**13b**): To a solution of **S4b** (133 mg, 0.28 mmol) in DMF (0.7 mL) was added sodium cyanide (21.0 mg, 0.43 mmol) at room temperature. The resulting mixture was allowed to warm to 90 °C and stirred for 20 min. The reaction mixture was quenched with sat. NaHCO_3_ aq, and the aqueous layer was extracted with ethyl acetate three times. The combined organic layer was washed with brine, dried over MgSO_4_, filtered, and concentrated in vacuo. The residue was purified by flash chromatography on silica gel (*n*-hexane/ethyl acetate = 80:1) to give **13b** (105 mg, 99%) as a yellow oil. [α]${}_{D}^{25}$ = –32.0 (*c* 2.14, CHCl_3_); ^1^H NMR (300 MHz, CDCl_3_) δ 4.27 (q, *J* = 2.1 Hz, 1H), 3.82 (td, *J* = 5.3, 3.2 Hz, 1H), 2.45–2.51 (m, 2H), 2.42 (d, *J* = 2.1 Hz, 1H), 1.89–2.12 (m, 2H), 0.90 (s, 18H), 0.11–0.15 (m, 12H); ^13^C NMR (75 MHz, CDCl_3_) δ 120.3, 83.0, 74.4, 73.7, 66.7, 28.2, 25.8, 25.7, 18.1, 13.0, −4.2, −4.6, −4.8, −5.2; HRMS (ESI) *m*/*z*: [M + Na]^+^ calcd for C_19_H_37_NO_2_Si_2_Na 390.2261, found 390.2249.

(5*S*,6*S*)-5-(but-3-en-1-yl)-6-ethynyl-2,2,3,3,8,8,9,9-octamethyl-4,7-dioxa-3,8-disiladecane (**8a**): To a solution of **13a** (42 mg, 0.11 mmol) in CH_2_Cl_2_ (0.6 mL) was added diisobutylaluminium hydride (1.0 M in hexane; 0.14 mL, 0.14 mmol) at 0 °C. The resulting mixture was stirred at the same temperature for 30 min. To the reaction mixture was added 2-propanol (0.098 mL), silica gel (200 mg), and water (1 mL) at room temperature. The resulting mixture was stirred at the same temperature for 30 min. The reaction mixture was filtered through a pad of Celite and concentrated in vacuo. The residue was purified by flash chromatography on silica gel (*n*-hexane/ethyl acetate = 10:1) to give the aldehyde as a yellow oil. The crude residue was used in the subsequent step without purification. To a solution of methyltriphenylphosphonium iodide (240 mg, 0.39 mmol) in THF (0.6 mL) was added sodium bis(trimethylsilyl)amide (1.9 M in THF, 0.29 mL, 0.56 mmol) at 0 °C. The resulting mixture was stirred at the same temperature for 30 min, then a solution of the aldehyde in THF (0.2 M, 0.55 mL, 0.11 mmol) was added dropwise at the same temperature. The resulting mixture was allowed to warm to room temperature and stirred for 1 h. The reaction mixture was quenched with sat. NH_4_Cl aq, and the aqueous layer was extracted with *n*-hexane three times. The combined organic layer was washed with brine, dried over MgSO_4_, filtered, and concentrated in vacuo. The residue was purified by flash chromatography on silica gel (*n*-hexane) to give **8a** (27 mg, 64%) as a colorless oil. [α]${}_{D}^{25}$ = –18.0 (*c* 0.22, CHCl_3_); ^1^H NMR (400 MHz, CDCl_3_) δ 5.78–5.88 (m, 1H), 4.94–5.06 (m, 2H), 4.34 (q, *J* = 2.3 Hz, 1H), 3.57–3.61 (m, 1H), 2.33 (d, *J* = 2.3 Hz, 1H), 2.18–2.27 (m, 1H), 2.02–2.11 (m, 1H), 1.78–1.86 (m, 1H), 1.67–1.75 (m, 1H), 0.90 (s, 18H), 0.07–0.14 (m, 12H); ^13^C NMR (100 MHz, CDCl_3_) δ 138.9, 114.3, 83.0, 74.0, 73.2, 66.8, 30.8, 29.6, 25.8, 25.8, 18.2, 18.1, −4.4, −4.5, −4.7, −5.0; HRMS (ESI) *m*/*z*: [M + Na]^+^ calcd for C_20_H_40_O_2_Si_2_Na 391.2465, found 391.2465.

(5*S*,6*R*)-5-(but-3-en-1-yl)-6-ethynyl-2,2,3,3,8,8,9,9-octamethyl-4,7-dioxa-3,8-disiladecane (**8b**): To a solution of **13b** (32.5 mg, 0.089 mmol) in CH_2_Cl_2_ (0.4 mL) was added diisobutylaluminium hydride (1.0 M in hexane; 0.11 mL, 0.11 mmol) at 0 °C. The resulting mixture was stirred at the same temperature for 30 min. To the reaction mixture was added 2-propanol (0.074 mL), silica gel (200 mg), and water (1 mL) at room temperature. The resulting mixture was stirred at the same temperature for 30 min. The reaction mixture was filtered through a pad of Celite and concentrated in vacuo. The residue was purified by flash chromatography on silica gel (*n*-hexane/ethyl acetate = 10:1) to give the aldehyde as a yellow oil. The crude residue was used in the subsequent step without purification. To a solution of methyltriphenylphosphonium iodide (189 mg, 0.47 mmol) in THF (0.5 mL) was added sodium bis(trimethylsilyl)amide (1.9 M in THF, 0.23 mL, 0.44 mmol) at 0 °C. The resulting mixture was stirred at the same temperature for 30 min, then a solution of the aldehyde in THF (0.2 M, 0.45 mL, 0.089 mmol) was added dropwise at the same temperature. The resulting mixture was allowed to warm to room temperature and stirred for 1 h. The reaction mixture was quenched with sat. NH_4_Cl aq, and the aqueous layer was extracted with *n*-hexane three times. The combined organic layer was washed with brine, dried over MgSO_4_, filtered, and concentrated in vacuo. The residue was purified by flash chromatography on silica gel (*n*-hexane) to give **8b** (23 mg, 70%) as a colorless oil. [α]${}_{D}^{25}$ = –29.1 (*c* 3.30, CHCl_3_); ^1^H NMR (300 MHz, CDCl_3_) δ 5.76–5.89 (m, 1H), 4.93–5.05 (m, 2H), 4.23 (q, *J* = 2.3 Hz, 1H), 3.73 (q, *J* = 5.2 Hz, 1H), 2.35 (d, *J* = 2.4 Hz, 1H), 2.13 (q, *J* = 7.3 Hz, 2H), 1.59–1.82 (m, 2H), 0.91 (s, 18H), 0.08–0.15 (m, 12H); ^13^C NMR (75 MHz, CDCl_3_) δ 138.8, 114.3, 84.2, 75.2, 73.3, 66.7, 32.0, 28.9, 26.0, 25.8, 18.2, −4.1, −4.5, −4.5, −5.1; HRMS (ESI) *m*/*z*: [M + Na]^+^ calcd for C_20_H_40_O_2_Si_2_Na 391.2465, found 391.2442.

4α,25-dihydroxyvitamin D_3_ (**6a**): To a solution of CD-ring **7** [14] (19 mg, 0.040 mmol), **8a** (18 mg, 0.048 mmol), and triethylamine (0.4 mL) in toluene (0.4 mL) was added tetrakis(triphenylphosphine)palladium(0) (5 mg, 0.0040 mmol) at room temperature. The resulting mixture was allowed to warm to 90 °C and stirred for 2 h. The reaction mixture was concentrated in vacuo. The residue was purified by flash chromatography on silica gel (*n*-hexane) to give the coupling product (20 mg, 66%) as a yellow oil. The crude residue was used in the subsequent step without purification. To a solution of the coupling product (20 mg, 0.026 mmol) in THF (0.3 mL) was added tetra-*n*-butylammonium fluoride (1.0 M in THF, 0.26 mL, 0.26 mmol) at room temperature. The resulting mixture was stirred at the same temperature for 12 h. The reaction mixture was quenched with sat. NaHCO_3_ aq, and the aqueous layer was extracted with ethyl acetate three times. The combined organic layer was washed with brine, dried over MgSO_4_, filtered, and concentrated in vacuo. The residue was purified by flash chromatography on silica gel (methanol/chloroform 1:20) to give **6a** (8 mg, 77%) as a yellow oil. [α]${}_{D}^{25}$ = +162.5 (*c* 0.08, CHCl_3_); ^1^H NMR (400 MHz, CDCl_3_) δ 6.58 (d, *J* = 10.1 Hz, 1H), 6.04 (d, *J* = 11.4 Hz, 1H), 5.12 (s, 1H), 4.87 (s, 1H), 3.96 (d, *J* = 8.2 Hz, 1H), 3.57 (td, *J* = 8.9, 3.7 Hz, 1H), 2.90 (d, *J* = 12.8 Hz, 1H), 2.36 (td, *J* = 8.8, 4.4 Hz, 1H), 2.16–2.21 (m, 1H), 2.07–2.13 (m, 1H), 2.00 (t, *J* = 9.2 Hz, 2H), 1.88 (t, *J* = 8.2 Hz, 1H), 1.21–1.82 (m, 22H), 0.93 (d, *J* = 6.4 Hz, 3H), 0.53 (s, 3H); ^13^C NMR (100 MHz, CDCl_3_) δ 144.1, 143.5, 137.3, 119.1, 117.1, 114.6, 78.4, 74.6, 71.1, 56.5, 56.4, 52.1, 45.9, 44.4, 40.5, 36.4, 36.1, 32.2, 29.7, 29.4, 29.2, 27.7, 23.5, 22.2, 20.8, 18.8, 12.0; HRMS (ESI) *m*/*z*: [M + Na]^+^ calcd for C_27_H_44_O_3_Na 439.3188, found 439.3177.

4β,25-dihydroxyvitamin D_3_ (**6b**): To a solution of CD-ring **7** (38 mg, 0.080 mmol), **8b** (35 mg, 0.096 mmol), and triethylamine (0.8 mL) in toluene (0.8 mL) was added tetrakis(triphenylphosphine)palladium(0) (9 mg, 0.0080 mmol) at room temperature. The resulting mixture was allowed to warm to 90 °C and stirred for 2 h. The reaction mixture was concentrated in vacuo. The residue was purified by flash chromatography on silica gel (*n*-hexane) to give the coupling product (39 mg, 65%) as a yellow oil. The crude residue was used in the subsequent step without purification. To a solution of the coupling product (9 mg, 0.012 mmol) in THF (0.6 mL) was added tetra-*n*-butylammonium fluoride (1.0 M in THF, 0.12 mL, 0.12 mmol) at room temperature. The resulting mixture was stirred at the same temperature for 12 h. The reaction mixture was quenched with sat. NaHCO_3_ aq, and the aqueous layer was extracted with ethyl acetate three times. The combined organic layer was washed with brine, dried over MgSO_4_, filtered, and concentrated in vacuo. The residue was purified by flash chromatography on silica gel (methanol/chloroform 1:20) to give **6b** (5 mg, 100%) as a yellow oil. [α]${}_{D}^{25}$ = +39.0 (*c* 0.21, CHCl_3_); ^1^H NMR (500 MHz, CDCl_3_) δ 6.51 (d, *J* = 10.9 Hz, 1H), 6.04 (d, *J* = 11.5 Hz, 1H), 5.17 (s, 1H), 4.92 (s, 1H), 4.22 (s, 1H), 3.86 (s, 1H), 2.86 (d, *J* = 13.2 Hz, 1H), 2.35–2.38 (m, 1H), 2.13–2.18 (m, 1H), 2.00–2.02 (m, 2H), 1.84–1.85 (m, 3H), 1.21–1.71 (m, 21H), 0.93 (d, *J* = 6.3 Hz, 3H), 0.54 (s, 3H); ^13^C NMR (125 MHz, CDCl_3_) δ 145.4, 142.6, 137.2, 123.5, 117.0, 115.4, 71.9, 71.1, 56.5, 56.4, 46.0, 44.4, 40.5, 36.4, 36.1, 32.0, 30.1, 29.7, 29.4, 29.2, 29.2, 27.7, 23.6, 22.2, 20.8, 18.8, 12.0; HRMS (ESI) *m*/*z*: [M + Na]^+^ calcd for C_27_H_44_O_3_Na 439.3188, found 439.3181.

### 2.2. Metabolism of ***6a*** and ***6b*** by CYP24A1

The metabolism of **6a** and **6b** by CYP24A1 was examined using recombinant human CYP24A1 as described in previous studies [7,15]. Briefly, the reaction mixture containing 20 nM CYP24A1, 2.0 μM bovine adrenodoxin, 0.2 μM bovine adrenodoxin reductase, 5 μM each substrate, 1 mM NADPH, and 1 mM ethylenediaminetetraacetic acid (EDTA) in 100 mM Tris-HCl (pH 7.4) was incubated at 37 °C for 15 or 30 min. The metabolites were extracted with 4 volumes of chloroform/methanol (3:1) and analyzed by reversed-phase HPLC under the same conditions followed in our previous study [16].

### 2.3. Synthesis of ***16a*** and ***16b*** and Identification of New Metabolites

4α,24*R*,25-trihydroxyvitamin D_3_ (**16a**): To a solution of CD-ring **15** [17] (9.6 mg, 0.016 mmol), **8a** (5 mg, 0.014 mmol), and triethylamine (0.11 mL) in toluene (0.11 mL) was added tetrakis(triphenylphosphine)palladium(0) (3.2 mg, 0.0016 mmol) at room temperature. The resulting mixture was allowed to warm to 90 °C and stirred for 2 h. The reaction mixture was concentrated in vacuo. The residue was purified by flash chromatography on silica gel (*n*-hexane) to give the coupling product (11 mg, 88%) as a yellow oil. The crude residue was used in the subsequent step without purification. To a solution of the coupling product (11 mg, 0.012 mmol) in THF (0.1 mL) was added tetra-*n*-butylammonium fluoride (1.0 M in THF, 144 μL, 0.144 mmol) at room temperature. The resulting mixture was stirred at the same temperature for 12 h. The reaction mixture was quenched with sat. NaHCO_3_ aq, and the aqueous layer was extracted with ethyl acetate three times. The combined organic layer was washed with brine, dried over MgSO_4_, filtered, and concentrated in vacuo. The residue was purified by flash chromatography on silica gel (methanol/chloroform 1:15) to give **16a** (4.9 mg, 94%) as a yellow oil. [α]${}_{D}^{25}$ = +36.6 (*c* 0.29, CHCl_3_); ^1^H NMR (300 MHz, CDCl_3_) δ 6.59 (d, *J* = 11.4 Hz, 1H), 6.04 (d, *J* = 11.4 Hz, 1H), 5.12 (s, 1H), 4.88 (s, 1H), 3.96 (d, *J* = 8.6 Hz, 1H), 3.54–3.61 (m, 1H), 3.34 (t, *J* = 5.8 Hz, 1H), 2.90 (d, *J* = 12.4 Hz, 1H), 2.32–2.40 (m, 1H), 1.86–2.27 (m, 9H), 1.25–1.71 (m, 10H), 1.22 (s, 3H), 1.17 (s, 3H), 0.94 (d, *J* = 6.2 Hz, 3H), 0.54 (s, 3H); ^13^C NMR (125 MHz, CD_3_OD) δ 146.0, 144.1, 140.0, 120.7, 118.7, 114.2, 79.7, 79.2, 75.0, 73.9, 58.1, 57.6, 47.0, 41.9, 37.2, 34.2, 33.2, 32.7, 30.1, 28.8, 28.7, 25.7, 24.9, 24.6, 23.3, 19.3, 12.4; HRMS (ESI) *m*/*z*: [M + Na]^+^ calcd for C_27_H_44_O_4_Na 455.3137, found 455.3120.

4β,24*R*,25-trihydroxyvitamin D_3_ (**16b**): To a solution of CD-ring **15** (9.6 mg, 0.016 mmol), **8b** (5 mg, 0.014 mmol), and triethylamine (0.11 mL) in toluene (0.11 mL) was added tetrakis(triphenylphosphine)palladium(0) (3.2 mg, 0.0016 mmol) at room temperature. The resulting mixture was allowed to warm to 90 °C and stirred for 2 h. The reaction mixture was concentrated in vacuo. The residue was purified by flash chromatography on silica gel (*n*-hexane) to give the coupling product (8.5 mg, 68%) as a yellow oil. The crude residue was used in the subsequent step without purification. To a solution of the coupling product (8.5 mg, 0.0096 mmol) in THF (0.1 mL) was added tetra-*n*-butylammonium fluoride (1.0 M in THF, 115 μL, 0.115 mmol) at room temperature. The resulting mixture was stirred at the same temperature for 12 h. The reaction mixture was quenched with sat. NaHCO_3_ aq, and the aqueous layer was extracted with ethyl acetate three times. The combined organic layer was washed with brine, dried over MgSO_4_, filtered, and concentrated in vacuo. The residue was purified by flash chromatography on silica gel (methanol/chloroform 1:15) to give **16b** (3.5 mg, 84%) as a yellow oil. [α]${}_{D}^{25}$ = +47.7 (*c* 0.13, CHCl_3_); ^1^H NMR (300 MHz, CDCl_3_) δ 6.51 (d, *J* = 11.4 Hz, 1H), 6.04 (d, *J* = 11.4 Hz, 1H), 5.17 (s, 1H), 4.92 (s, 1H), 4.22 (d, *J* = 2.8 Hz, 1H), 3.84–3.88 (m, 1H), 3.65 (s, 1H), 3.34 (t, *J* = 5.8 Hz, 1H), 2.86 (d, *J* = 12.4 Hz, 1H), 2.33–2.42 (m, 1H), 2.11–2.20 (m, 1H), 1.17–2.04 (m, 23H), 0.94 (d, *J* = 6.2 Hz, 3H), 0.55 (s, 3H); ^13^C NMR (125 MHz, CD_3_OD) δ 144.9, 139.9, 123.4, 118.6, 115.0, 79.8, 78.1, 73.9, 73.1, 58.1, 57.6, 47.1, 41.9, 37.2, 34.2, 33.2, 30.5, 30.1, 28.8, 28.7, 25.7, 24.9, 24.6, 23.3, 19.3, 12.4; HRMS (ESI) *m*/*z*: [M + Na]^+^ calcd for C_27_H_44_O_4_Na 455.3137, found 455.3117.

### 2.4. Docking Study

The initial structure of the human CYP24A1 was obtained from the AlphaFold protein structure database [18] (AF-Q07973-F1-model_v2.pdb) and refined for docking simulations using the Protein Preparation Wizard script within Maestro (Schrödinger, LLC, New York, NY, USA). For all compound molecules, ionization and energy minimization were performed using the OPLS3e force field in the LigPrep script in Maestro. These minimized structures were used as input structures for the docking simulations using the Glide SP docking [19,20] program (Schrödinger, LLC, New York, NY, USA), with a grid box defined by a potential binding site position from SiteMap [21,22] (Schrödinger, LLC, New York, NY, USA). In docking simulations, we also introduced hydrogen-bonding constraints between the sidechain of The395 and any polar atoms in the compound molecules, because this hydrogen-bonding formation is a key interaction in known complexes of CYP24A1 bound to the vitamin D_3_ analogs [23]. After the docking simulations were completed, the lowest distances between the Fe atom of the HEM molecule and the C24 of the compounds from 100 poses on a binding site were selected.

## 3. Results and Discussion

### 3.1. Synthesis of 4α,25-Dihydroxyvitamin D_3_ (***6a***) and 4β,25-Dihydroxyvitamin D_3_ (***6b***)

**Scheme 2:**
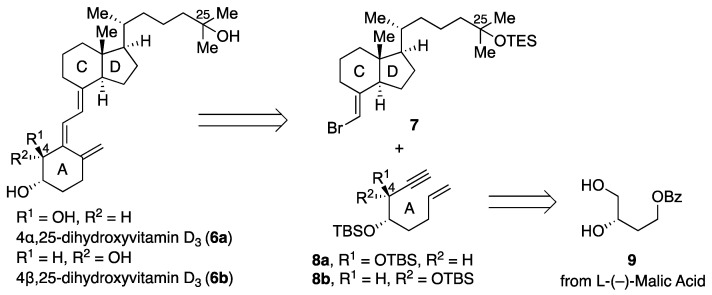
Retrosynthetic analysis for 4α,25-dihydroxyvitamin D_3_ (**6a**) and 4β,25-dihydroxyvitamin D_3_ (**6b**).

The retrosynthetic plan for the synthesis of 4α,25-dihydroxyvitamin D_3_ (**6a**) and 4β,25-dihydroxyvitamin D_3_ (**6b**) is depicted in Scheme 2. We planned to construct the triene system in **6** using a palladium-catalyzed coupling reaction [24] of the bromoolefin **7** [24] (CD-ring) and the A-ring precursor enyne **8**. The synthesis of enyne **8** was planned starting from diol **9**, which can be derived from L-(–)-malic acid [13].

**Scheme 3:**
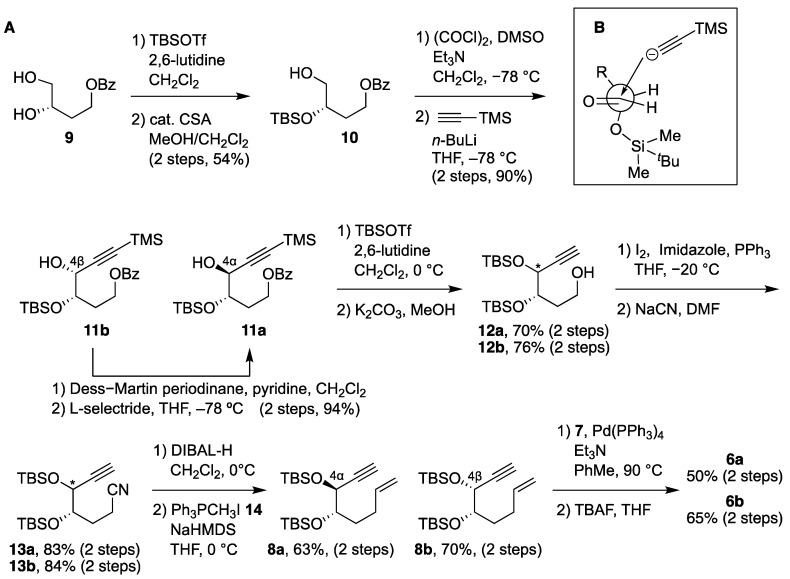
(**A**) Synthesis of **6a** and **6b**. (**B**) Felkin–Anh model for the reaction of the aldehyde derived from **10** with TMS acetylide.

The syntheses of 4α,25-dihydroxyvitamin D_3_ (**6a**) and 4β,25-dihydroxyvitamin D_3_ (**6b**) are illustrated in Scheme 3A. The primary hydroxy alcohol **10** was synthesized in 54% yield by the reaction of **9**, derived from L-(–)-malic acid, with TBSOTf followed by selective deprotection of the primary TBS ether in the presence of camphorsulfonic acid (CSA). After oxidation of the primary hydroxy group in **10** under Swern conditions, the resulting aldehyde was reacted with TMS acetylene in the presence of *n*-butyllithium to stereoselectively give 4β-**11b** in 90% yield (2 steps), where the stereochemistry at C4 is controlled according to the Felkin–Anh model (Scheme 3B). The 4α-isomer **11a** was stereoselectively obtained by oxidation of **11b** with Dess–Martin periodinane followed by reduction of the resulting ketone with a bulky reducing agent, L-selectride. The alcohols **11a** and **11b** were then converted to **6a** and **6b**, respectively, as follows. The secondary hydroxy groups in **11a** and **11b** were protected as TBS ethers in the presence of TBSOTf and 2,6-lutidine, and the benzoyl and TMS groups were removed with potassium carbonate to give alcohols **12a** and **12b** in 70% and 76% yields, respectively. Treatment of **12a** and **12b** with iodine and PPh_3_ followed by reaction with sodium cyanide gave the corresponding nitriles **13a** and **13b**. Reduction of the nitriles **13** with DIBAL-H afforded the corresponding aldehydes, which were then reacted with Wittig reagent **14** to give the A-ring synthons, enynes **8a** and **8b**, in 63% and 70% yield, respectively. Coupling of **8a** and **8b** with the CD-ring synthon, bromoolefin **7**, was performed in the presence of a Pd catalyst, and the coupling products were deprotected with TBAF to remove the silyl ether groups, affording vitamin D metabolites **6a** and **6b** in 50% yield and 65% yield, respectively.

### 3.2. Metabolism of ***6a*** and ***6b*** by CYP24A1

A reconstituted system containing human CYP24A1, adrenodoxin reductase, and adrenodoxin was employed to examine the metabolism of **6a** and **6b**. The conversion ratios of the substrate to its metabolites were obtained from the peak area ratio in HPLC chromatograms after 15 min incubation of **6** with CYP24A1 (Figure S1). The **6a** was sequentially metabolized by CYP24A1 to produce multiple metabolites, as well as 1,25D_3_ and 25D_3_ [6,7]. The total conversion ratio of **6a** to the multiple metabolites was 38.2%, whereas the conversion ratio of **6b** was only 9.8%. These results suggest that the difference in serum concentrations between **6a** and **6b** can be explained by the difference in their metabolic stability, as we had hypothesized. Specifically, the high resistance of **6b** to metabolism by CYP24A1 may explain why **6b** is detected in serum, whereas **6a** is not.

### 3.3. Identification of ***16a*** and ***16b*** as Metabolites of 4,25-(OH)_2_-D_3_ (***6***)

Next, we set out to identify the major metabolites of **6a** and **6b** generated by CYP24A1. Since CYP24A1 is known to hydroxylate C24 of **2** with *R*-stereochemistry [7], we hypothesized that **6** would undergo 24*R*-hydroxylation by CYP24A1 to afford 4,24*R*,25-trihydroxyvitamin D_3_ (**7**). To test this hypothesis, we synthesized the 4α compound **16a** and the 4β compound **16b** and compared their HPLC retention times with the metabolites of **6a** and **6b** produced by CYP24A1.

**Scheme 4:**
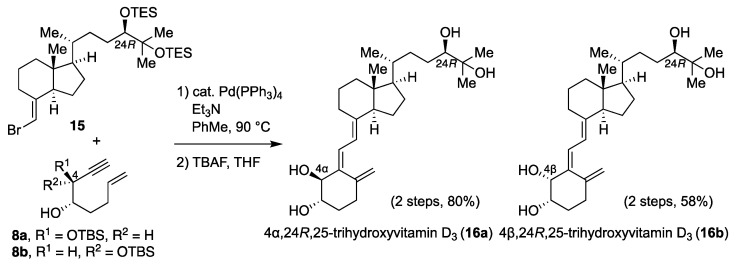
Synthesis of **16a** and **16b**.

Compounds **16a** and **16b** were synthesized by a similar procedure to that described for the 4-hydroxylated metabolites of **6** (Scheme 4). Specifically, the CD ring **15** [17] bearing an *R*-hydroxy group at C24 was reacted with the A-ring precursor enyne **8a** or **8b** in the presence of a palladium catalyst to give the coupling product; deprotection of the silyl ether using TBAF afforded **16a** and **16b** in 80% and 58% yields, respectively.

**Figure 1:**
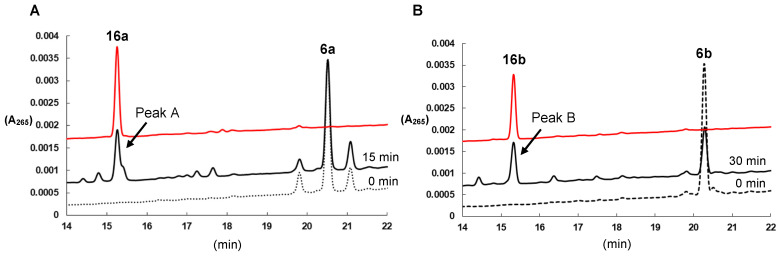
(**A**) HPLC profiles of **16a** (red line) and metabolites of **6a** after incubation with CYP24A1 for 0 min (dotted line) and 15 min (solid line). Peak A, marked with an arrow, indicates a major metabolite. (**B**) HPLC profiles of **16b** (red line) and metabolites of **6b** after incubation with CYP24A1 for 0 min (dotted line) and 30 min (solid line) after incubation with CYP24A1. Peak B, marked with an arrow, indicates a major metabolite.

Then, we compared the HPLC retention times of the synthesized **16a** and **16b** with those of the metabolites produced from **6** by CYP24A1. The retention times of **16a** and **16b** were consistent with those of the main metabolites produced by CYP24A1 from **6a** and **6b** (peak A and peak B in Figure 1A,B), respectively. In HPLC analyses on a chiral column (SUMICHIRAL OA-7000), the retention times of **16** matched those of the metabolites generated from **6** (Figure S2), indicating that the main metabolites produced by CYP24A1 from **6a** and **6b** are **16a** and **16b**, respectively. The conversion ratios to **16a** and **16b** from **6a** and **6b** were 25.6 and 7.0 %, respectively (Table 1). 

### 3.4. Docking Study

**Figure 2:**
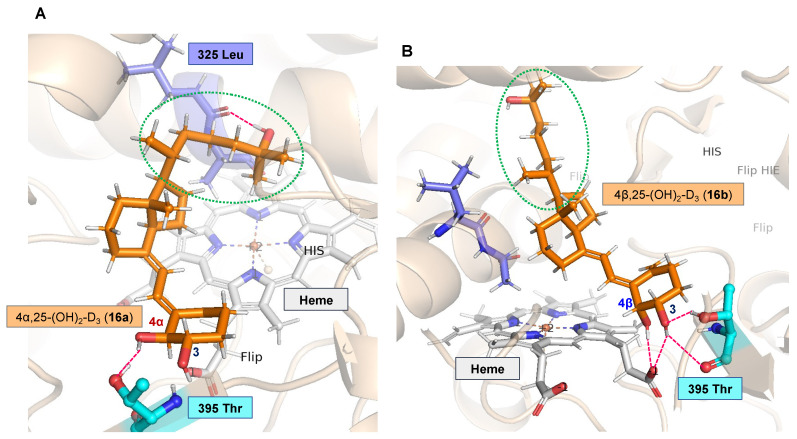
(**A**) Docking model of **6a** and CYP24A1. (**B**) Docking model of **6b** and CYP24A1.

To investigate the reason for the difference in metabolic stability between **6a** and **6b**, docking studies with CYP24A1 and **6a** and **6b** were carried out. In the docking of **6a** with CYP24A1, hydrogen bonds are formed between the C25 and C4α hydroxy groups of **6a** and Leu325 and Thr395 of CYP24A1, resulting in the formation of a stable complex in which the side chain of the CD-ring in **6a** is located close to the heme iron (Figure 2A). In contrast, in the docking of **6b** with CYP24A1, hydrogen bonds were formed between the C4β and C3 hydroxy groups of **6b** and the carboxylic acid of the side chain in heme and Thr395 of CYP24A1 (Figure 2B). In this case, the side chain of the CD ring in **6b** is located away from the heme iron, and the hydrogen bond between the C25 hydroxy group and the Leu325, which plays an important role in the metabolism of vitamin D by CYP24A1 [24], is not formed. This may explain why **6b** is less susceptible to metabolism by CYP24A1.

## 4. Conclusions

4α,25-Dihydroxyvitamin D_3_ (**6a**) and 4β,25-dihydroxyvitamin D_3_ (**6b**) are both produced from 25-hydroxyvitamin D_3_ (**2**) by CYP3A4, but **6b** is detectable in serum, whereas **6a** is not. Our findings show that **6a** is a better substrate of CYP24A1 than **6b**, and thus the greater metabolic stability of **6b** can account for its presence in human serum. Furthermore, major metabolites of **6a** and **6b** generated by CYP24A1 were identified as 4,24*R*,25-trihydroxyvitamin D_3_ (**16a**, **16b**) by HPLC, by comparison with synthesized authentic samples. Docking studies indicated that while **6a** can form a hydrogen bond between the hydroxy group at C25 and Leu325 in CYP24A1, the presence of the 4β-hydroxy group in **6b** prevents the formation of this hydrogen bond, which plays a crucial role in the metabolic activity of CYP24A1.

## Figures and Tables

**Table 1:** Evaluation of metabolic conversion ratios by human CYP24A1.

Substrate	Total Conversion Ratio (%)	Conversion Ratio to the Major Metabolite (%)
4α,25-dihydroxyvitamin D_3_ (**6a**)	38.2 ± 1.2	25.6 ± 0.3 (**16a**)
4β,25-dihydroxyvitamin D_3_ (**6b**)	9.8 ± 3.8	7.0 ± 2.7 (**16b**)

## Data Availability

Not applicable.

## Supplementary Materials

The following supporting information can be downloaded at: https://www.mdpi.com/article/10.3390/biom13071036/s1, Figure S1: HPLC profiles of metabolism of **6a** and **6b** by CYP24A1, Figure S2: HPLC profiles of metabolism of **6a** and **6b** by CYP24A1 with chiral column, Figure S3: 1H and 13C NMR Spectra.

## References

[1] Bouillon R., Okamura W.H., Norman A.W. (1995). Structure-Function Relationships in the Vitamin D Endocrine System. Endocr. Rev..

[2] Lieben L., Masuyama R., Torrekens S., Looveren R.V., Schrooten J., Baatsen P., Lafage-Proust M., Dresselaers T., Feng J.Q., Bonewald L.F. (2012). Normocalcemia is maintained in mice under conditions of calcium malabsorption by vitamin D–induced inhibition of bone mineralization. J. Clin. Investig..

[3] Kato S., Sekine K., Matsumoto T., Yoshizawa T. (1998). Molecular genetics of vitamin D receptor acting in bone. J. Bone Miner. Metab..

[4] Abe E., Miyaura C., Sakagami H., Takada M., Konno K., Yamazaki T., Yoshiki S., Suda T. (1981). Differentiation of mouse myeloid leukemia cells induced by 1α,25-dihydroxyvitamin D_3_. Proc. Natl. Acad. Sci. USA.

[5] Liu P.T., Stenger S., Li H., Wenzel L., Tan B.H., Krutzik S.R., Ochoa M.T., Schauber J., Wu K., Meinken C. (2006). Toll-like receptor triggering of a vitamin D-mediated human antimicrobial response. Science.

[6] Sakaki T., Sawada N., Nonaka Y., Ohyama Y., Inouye K. (1999). Metabolic studies using recombinant *Escherichia coli* cells producing rat mitochondrial CYP24. CYP24 can convert 1α,25-dihydroxyvitamin D_3_ to calcitroic acid. Eur. J. Biol. Chem..

[7] Sakaki T., Sawada N., Komai K., Shiozawa S., Yamada S., Yamamoto K., Ohyama Y., Inouye K. (2000). Dual metabolic pathway of 25-hydroxyvitamin D_3_ catalyzed by human CYP24. Eur. J. Biochem..

[8] Yamada S., Nakayama K., Takayama H., Shinki T., Takasaki Y., Suda T. (1984). Isolation, identification, and metabolism of (23*S*,25*R*)-25-hydroxyvitamin D_3_ 26,23-lactol. A biosynthetic precursor of (23*S*,25*R*)-25-hydroxyvitamin D_3_ 26,23-lactone. J. Biol. Chem..

[9] Ishizuka S., Norman A.W. (1987). Metabolic pathways from 1α,25-dihydroxyvitamin D_3_ to 1α,25-dihydroxyvitamin D_3_-26,23-lactone. Stereo-retained and stereo-selective lactonization. J. Biol. Chem..

[10] Yu O.B., Arnold L.A. (2016). Calcitroic Acid—A Review. ACS Chem. Biol..

[11] Kaufmann M., Martineau C., Arabian A., Traynor M., St-Arnaud R., Jones G. (2019). Calcioic acid: In vivo detection and quantification of the terminal C24-oxidation product of 25-hydroxyvitamin D_3_ and related intermediates in serum of mice treated with 24,25-dihydroxyvitamin D_3_. J. Steroid Biochem. Mol. Biol..

[12] Wang Z., Lin Y.S., Zheng X.E., Senn T., Hashizume T., Scian M., Dickmann L.J., Nelson S.D., Baillie T.A., Hebert M.F. (2012). An Inducible Cytochrome P450 3A4-Dependent Vitamin D Catabolic Pathway. Mol. Pharmacol..

[13] Hanessian S., Ugolini D., Dubé D., Glamyan A. (1984). Facile access to (*S*)-1,2,4-butanetriol and its derivatives. Can. J. Chem..

[14] Gogoi P., Sigüeiro R., Eduardo S., Mouriño A. (2010). An Expeditious Route to 1α,25-Dihydroxyvitamin D_3_ and Its Analogues by an Aqueous Tandem Palladium-Catalyzed A-Ring Closure and Suzuki Coupling to the C/D Unit. Chem. Eur. J..

[15] Urushino N., Yasuda K., Ikushiro S., Kamakura M., Ohta M., Sakaki T. (2009). Metabolism of 1α,25-dihydroxyvitamin D_2_ by human CYP24A1. Biochem. Biophys. Res. Commun..

[16] Kawagoe F., Mototani S., Yasuda K., Mano H., Sakaki T., Kittaka A. (2021). Stereoselective Synthesis of 24-Fluoro-25-Hydroxyvitamin D_3_ Analogues and Their Stability to hCYP24A1-Dependent Catabolism. Int. J. Mol. Sci..

[17] Sakamoto R., Nagata A., Ohshita H., Mizumoto Y., Iwaki M., Yasuda K., Sakaki T., Nagasawa K. (2021). Chemical Synthesis of Side-Chain-Hydroxylated Vitamin D_3_ Derivatives and Their Metabolism by CYP27B1. ChemBioChem.

[18] Tunyasuvunakool K., Adler J., Wu Z., Green T., Zielinski M., Žídek A., Bridgland A., Cowie A., Meyer C., Laydon A. (2021). Highly accurate protein structure prediction for the human proteome. Nature.

[19] Friesner R.A., Banks J.L., Murphy R.B., Halgren T.A., Klicic J.J., Mainz D.T., Repasky M.P., Knoll E.H., Shelley M., Perry J.K. (2004). Glide: A new approach for rapid, accurate docking and scoring. 1. Method and assessment of docking accuracy. J. Med. Chem..

[20] Halgren T.A., Murphy R.B., Friesner R.A., Beard H.S., Frye L.L., Pollard W.T., Banks J.L. (2004). Glide: A new approach for rapid, accurate docking and scoring. 2. Enrichment factors in database screening. J. Med. Chem..

[21] Halgren T. (2007). New method for fast and accurate binding-site identification and analysis. Chem. Biol. Drug Des..

[22] Halgren T.A. (2009). Identifying and characterizing binding sites and assessing druggability. J. Chem. Inf. Model..

[23] Rhieu S.Y., Annalora A.J., Gathungu R.M., Vouros P., Uskokovic M.R., Schuster I., Palmore G.T.R., Reddy G.S. (2011). A new insight into the role of rat cytochrome P450 24A1 in metabolism of selective analogs of 1α,25-dihydroxyvitamin D_3_. Arch. Biochem. Biophys..

[24] Trost B.M., Dumas J., Villa M. (1992). New strategies for the synthesis of vitamin D metabolites via palladium-catalyzed reactions. J. Am. Chem. Soc..

