# Supplementary material for: Differential Metabolic Stability of 4α,25- and 4β,25-Dihydroxyvitamin D3 and Identification of Their Metabolites
Source: Biomolecules. 2023 Jun 24;13(7):1036. doi: 10.3390/biom13071036 (PMC10377336; doi:10.3390/biom13071036)
Supplement: Supplementary file 1 [file biomolecules-13-01036-s001.zip › biomolecules-2452880-supplementary.pdf]

Differential metabolic stability of 4α,25- and 4β,25-dihydroxyvitamin D_3 and identification of their metabolites

Yuka Mizumoto,[1] Ryota Sakamoto,[1] Kazuto Ijima,[1] Naoto Nakaya,[2] Minami Odagi,[1] Masayuki Tera,[1] Takatsugu Hirokawa,[3,4] Toshiyuki Sakaki,[2,*] Kaori Yasuda,[2,*] Kazuo Nagasawa[1,*]

1 Department of Biotechnology and Life Science, Faculty of Engineering, Tokyo University of Agriculture and Technology, 2-24-16, Naka-cho, Koganei 184-8588, Tokyo, Japan
2 Faculty of Engineering, Toyama Prefectural University, 5180 Kurokawa, Imizu 939-0398, Toyama, Japan
3 Transborder Medical Research Center, University of Tsukuba, 1-1-1 Tennodai, Tsukuba 305-8575, Ibaraki, Japan
4 Division of Biomedical Science, Faculty of Medicine, University of Tsukuba, 1-1-1 Tennodai, Tsukuba 305-8575, Ibaraki, Japan
* Correspondence: tsakaki@pu-toyama.ac.jp (T.S.); kyasuda@pu-toyama.ac.jp (K.Y.); knaga@cc.tuat.ac.jp (K.N.)

Table of Contents

1. HPLC profiles of metabolism of **6a** and **6b** by CYP24A1.. S2
2. HPLC profiles of metabolism of **6a** and **6b** by CYP24A1 with chiral column………….... S3
3. ^{1}H and ^{13}C NMR Spectra.. S4

1. HPLC profiles of metabolism of **6a** and **6b** by CYP24A1

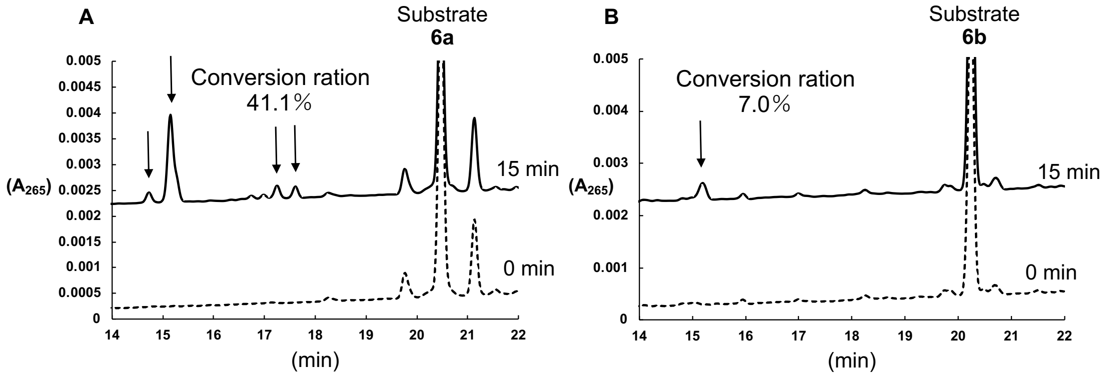

Figure S1. A) HPLC profiles of metabolites of **6a** at 0 min (dotted line) and 15 min (solid line) after incubation with CYP24A1. The peaks marked with arrows indicate metabolites. **B)** HPLC profiles of metabolites of **6b** at 0 min (dotted line) and 15 min (solid line) after incubation with CYP24A1. The peaks marked with arrows indicate metabolites.

2. HPLC profiles of metabolism of **6a** and **6b** by CYP24A1 with chiral column

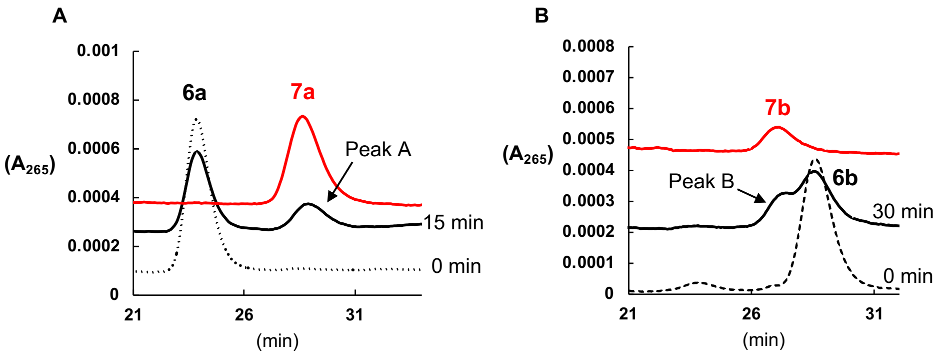

Figure S2. A) HPLC profiles of **7a** (red line), and metabolites of **6a** after incubation with CYP24A1 for 0 min (dotted line) and 15 min (solid line). Peak A marked with an arrow indicates a major metabolite. **B)** HPLC profiles of **7b** (red line), and metabolites of **6b** after incubation with CYP24A1 for 0 min (dotted line) and 30 min (solid line) after incubation with CYP24A1. Peak B marked with an arrow indicates a major metabolite. HPLC was performed under the following conditions: column, SUMICHIRAL OA-7000 (Sumika Chemical Analysis Service ,Ltd., Tokyo, Japan); UV detection, 265 nm; flow-rate, 0.7 mL/min; mobile phase, CH_3CN:H_2O = 88:12 (isocratic).

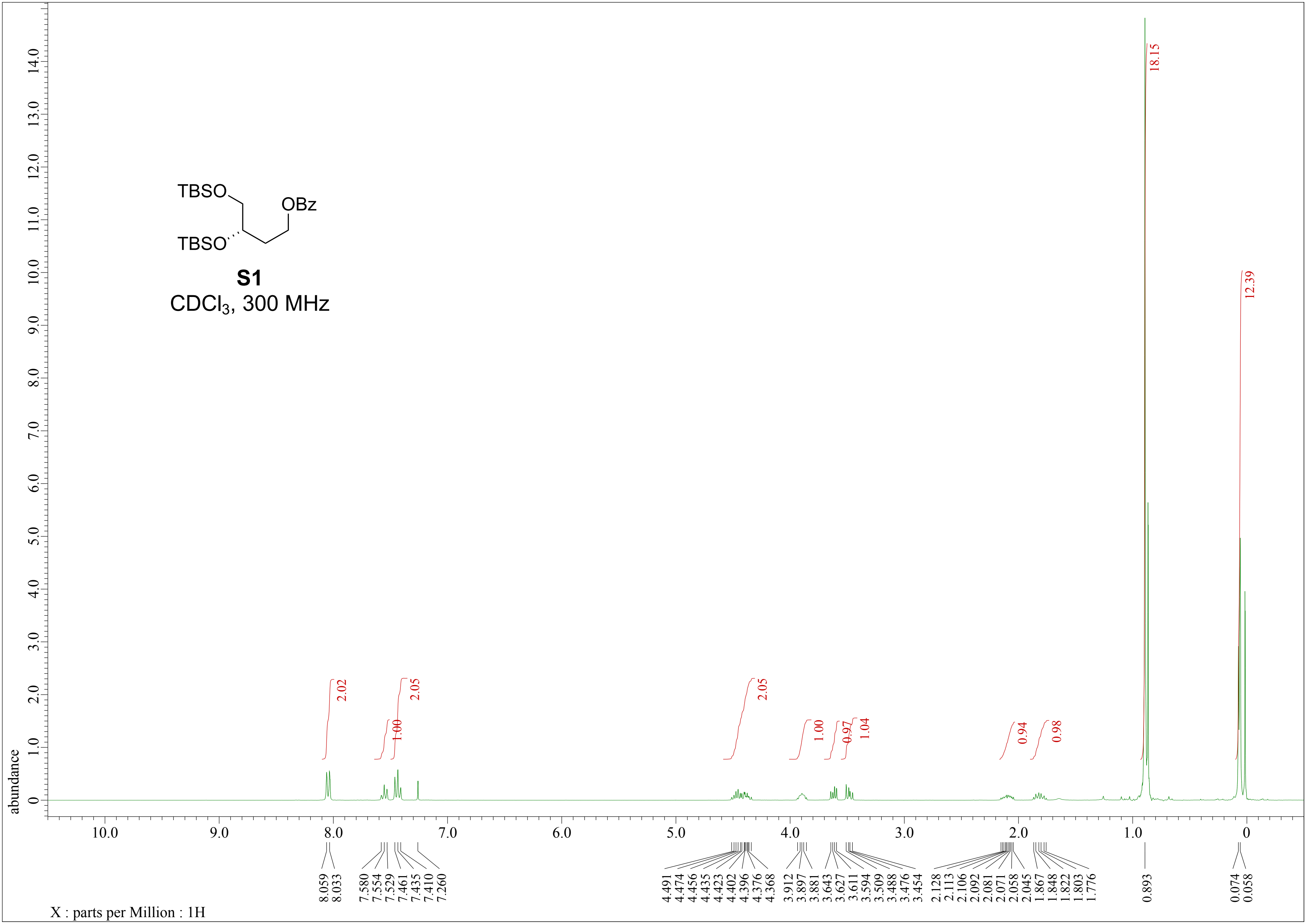
TBSO
TBSO
OBz
S1
CDCl3, 300 MHz
abundance
X : parts per Million : 1H
18.15
12.39
2.02
1.00
2.05
2.05
1.00
0.97
1.04
0.94
0.98
8.059
8.033
7.580
7.554
7.529
7.461
7.435
7.410
7.260
4.491
4.474
4.456
4.435
4.423
4.402
4.396
4.376
4.368
3.912
3.897
3.881
3.643
3.627
3.611
3.594
3.509
3.488
3.476
3.454
2.128
2.113
2.106
2.092
2.081
2.071
2.058
2.045
1.867
1.848
1.822
1.803
1.776
0.893
0.074
0.058

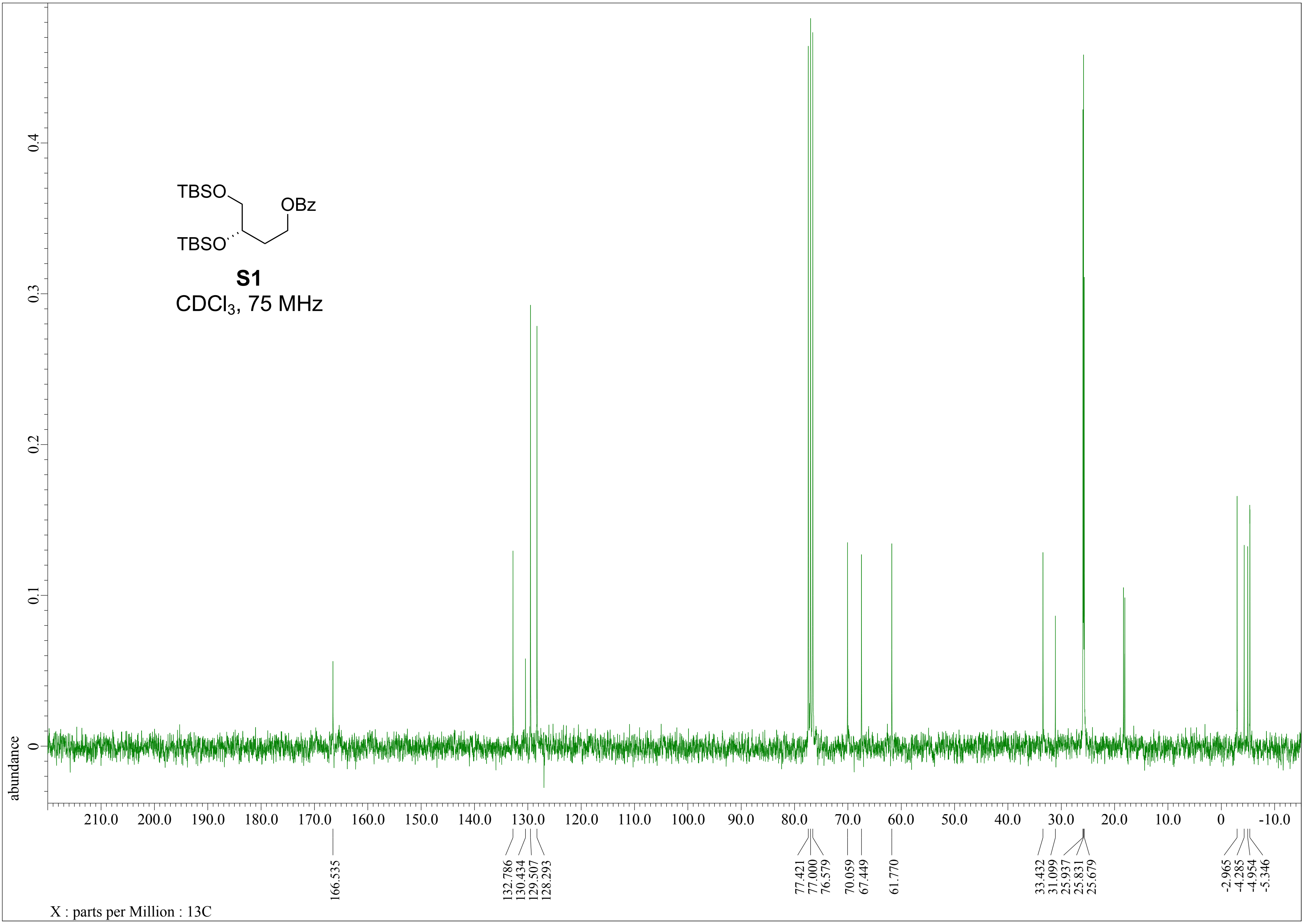
TBSO
OBz
TBSO
S1
CDCl3, 75 MHz
abundance
0.4
0.3
0.2
0.1
0
210.0 200.0 190.0 180.0 170.0 160.0 150.0 140.0 130.0 120.0 110.0 100.0 90.0 80.0 70.0 60.0 50.0 40.0 30.0 20.0 10.0 0 -10.0
166.535
132.786
130.434
129.507
128.293
77.421
77.000
76.579
70.059
67.449
61.770
33.432
31.099
25.937
25.831
25.679
-2.965
-4.285
-4.954
-5.346
X : parts per Million : 13C

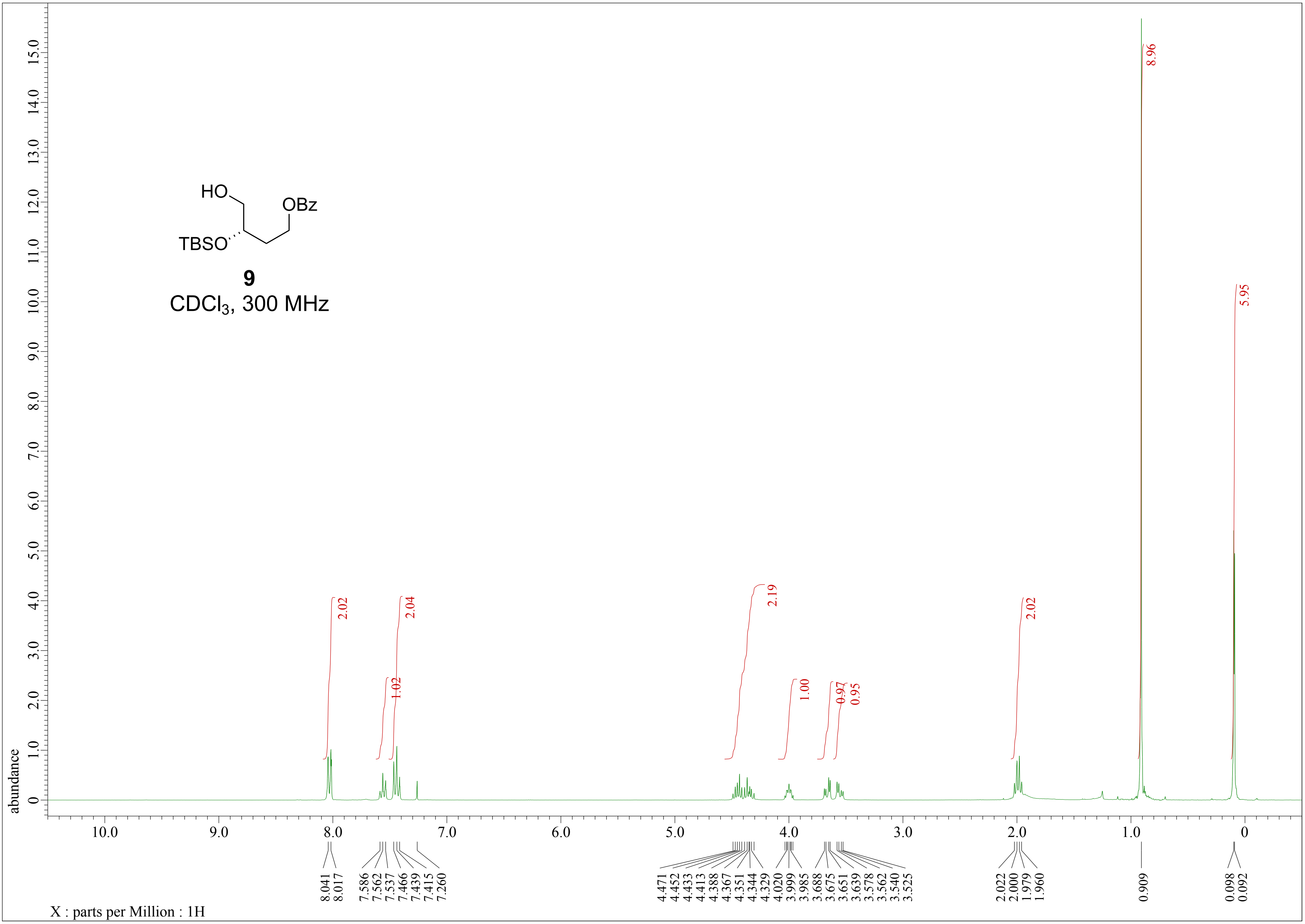
HO
OBz
TBSO
9
CDCl3, 300 MHz
abundance
X : parts per Million : 1H
8.041
8.017
7.586
7.562
7.537
7.466
7.439
7.415
7.260
4.471
4.452
4.433
4.413
4.388
4.367
4.351
4.344
4.329
4.020
3.999
3.985
3.688
3.675
3.651
3.639
3.578
3.562
3.540
3.525
2.022
2.000
1.979
1.960
0.909
0.098
0.092
2.02
1.02
2.04
2.19
1.00
0.97
0.95
2.02
8.96
5.95

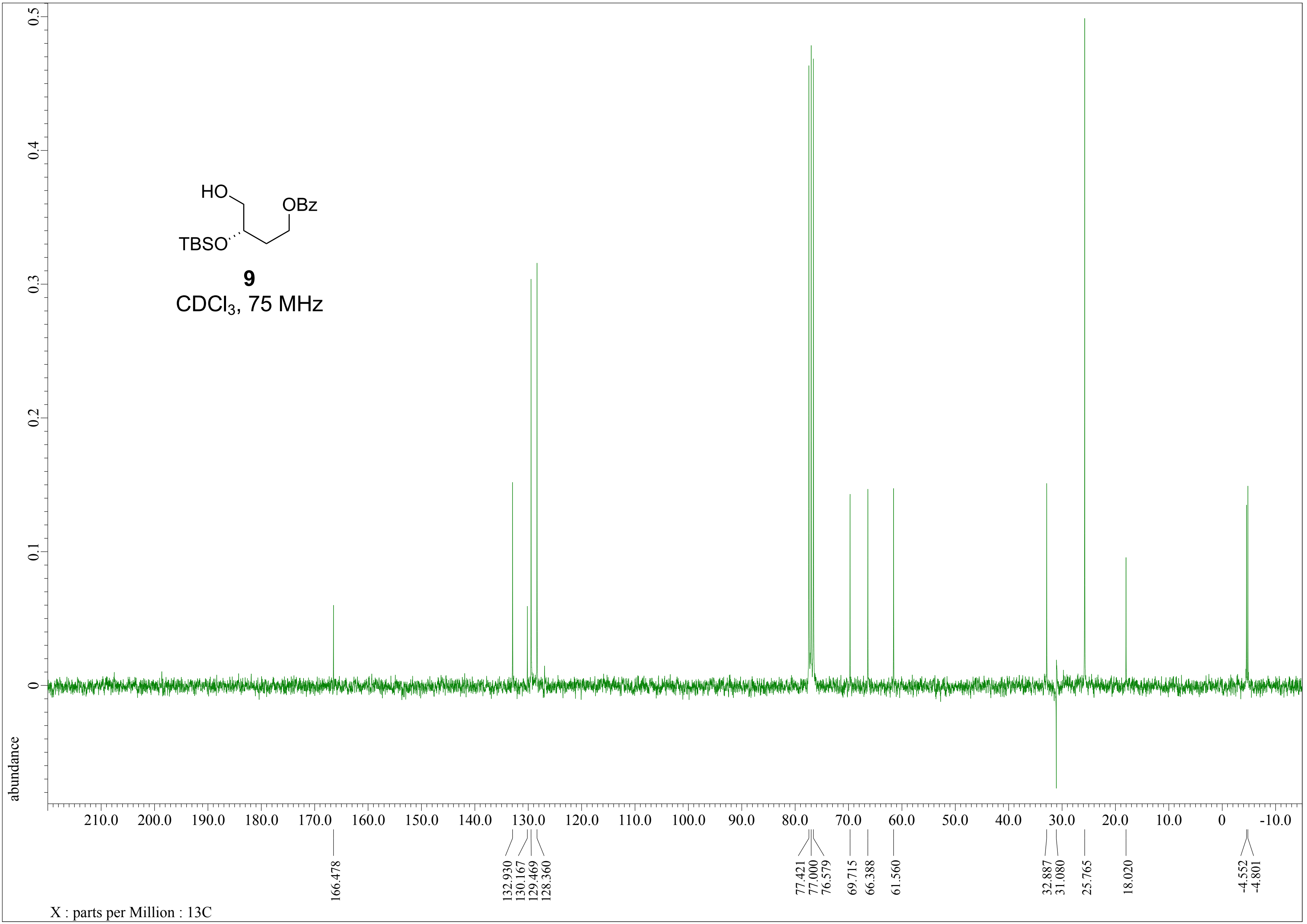
HO
OBz
TBSO
9
CDCl3, 75 MHz
0.5
0.4
0.3
0.2
0.1
0
abundance
210.0 200.0 190.0 180.0 170.0 160.0 150.0 140.0 130.0 120.0 110.0 100.0 90.0 80.0 70.0 60.0 50.0 40.0 30.0 20.0 10.0 0 -10.0
166.478
132.930
130.167
129.469
128.360
77.421
77.000
76.579
69.715
66.388
61.560
32.887
31.080
25.765
18.020
-4.552
-4.801
X : parts per Million : 13C

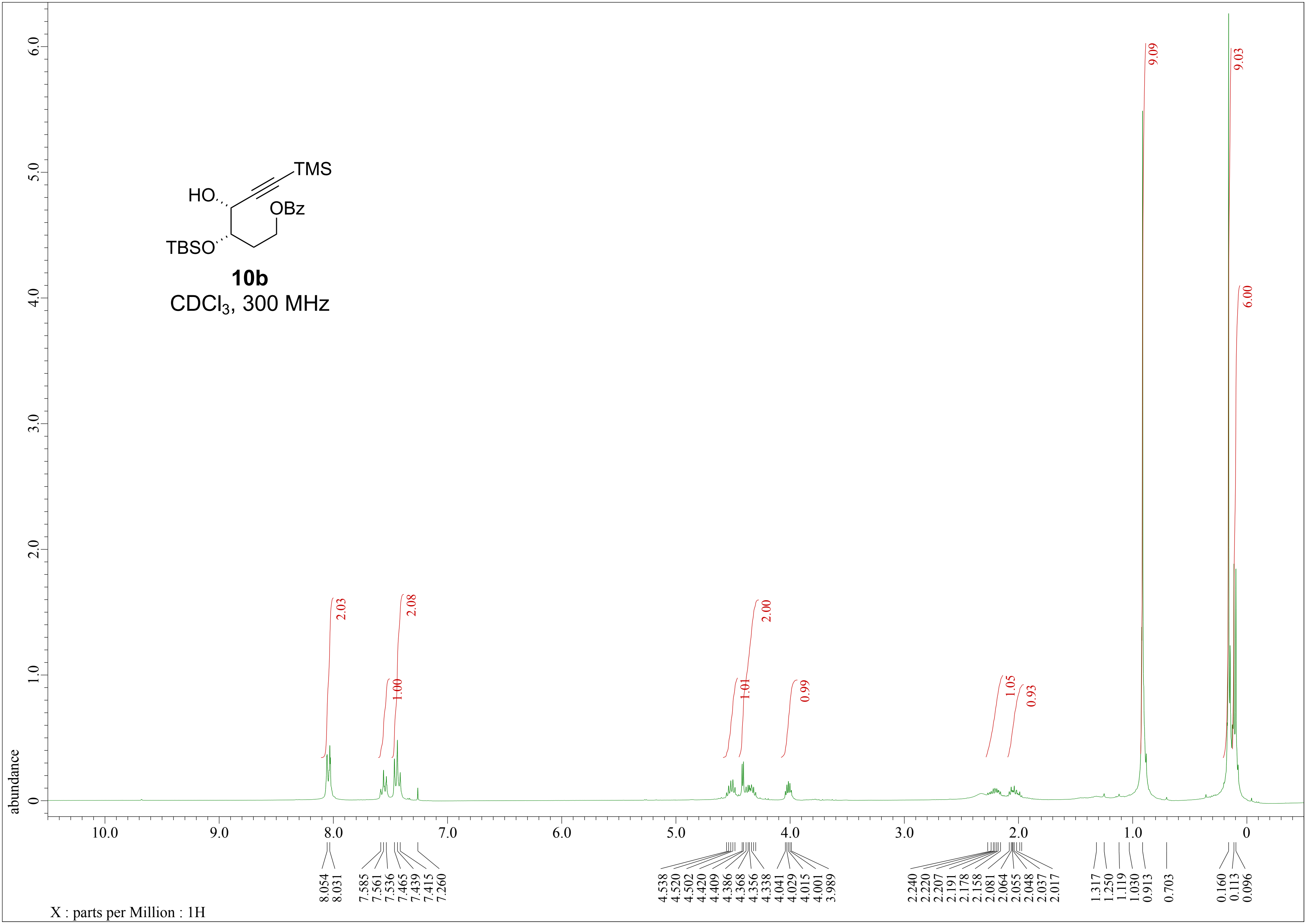

TMS
HO
OBz
TBSO
10b
CDCl3, 300 MHz
abundance
X : parts per Million : 1H
2.03
2.08
1.00
2.00
1.01
0.99
1.05
0.93
9.09
9.03
6.00
8.054
8.031
7.585
7.561
7.536
7.465
7.439
7.415
7.260
4.538
4.520
4.502
4.420
4.409
4.386
4.368
4.356
4.338
4.041
4.029
4.015
4.001
3.989
2.240
2.220
2.207
2.191
2.178
2.158
2.081
2.064
2.055
2.048
2.037
2.017
1.317
1.250
1.119
1.030
0.913
0.703
0.160
0.113
0.096

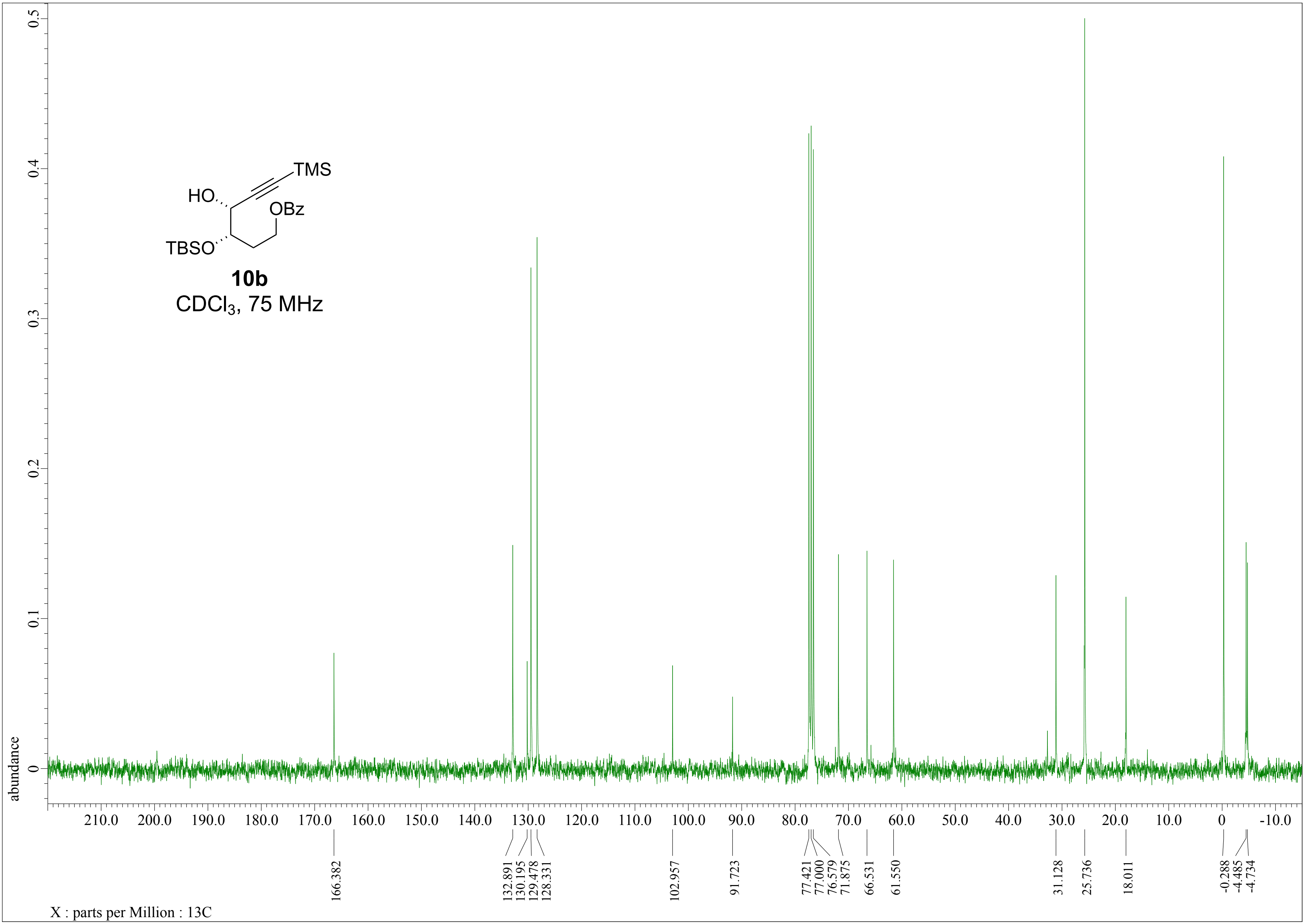
TMS
HO
OBz
TBSO
10b
CDCl3, 75 MHz
abundance
X : parts per Million : 13C
166.382
132.891
130.195
129.478
128.331
102.957
91.723
77.421
77.000
76.579
71.875
66.531
61.550
31.128
25.736
18.011
-0.288
-4.485
-4.734

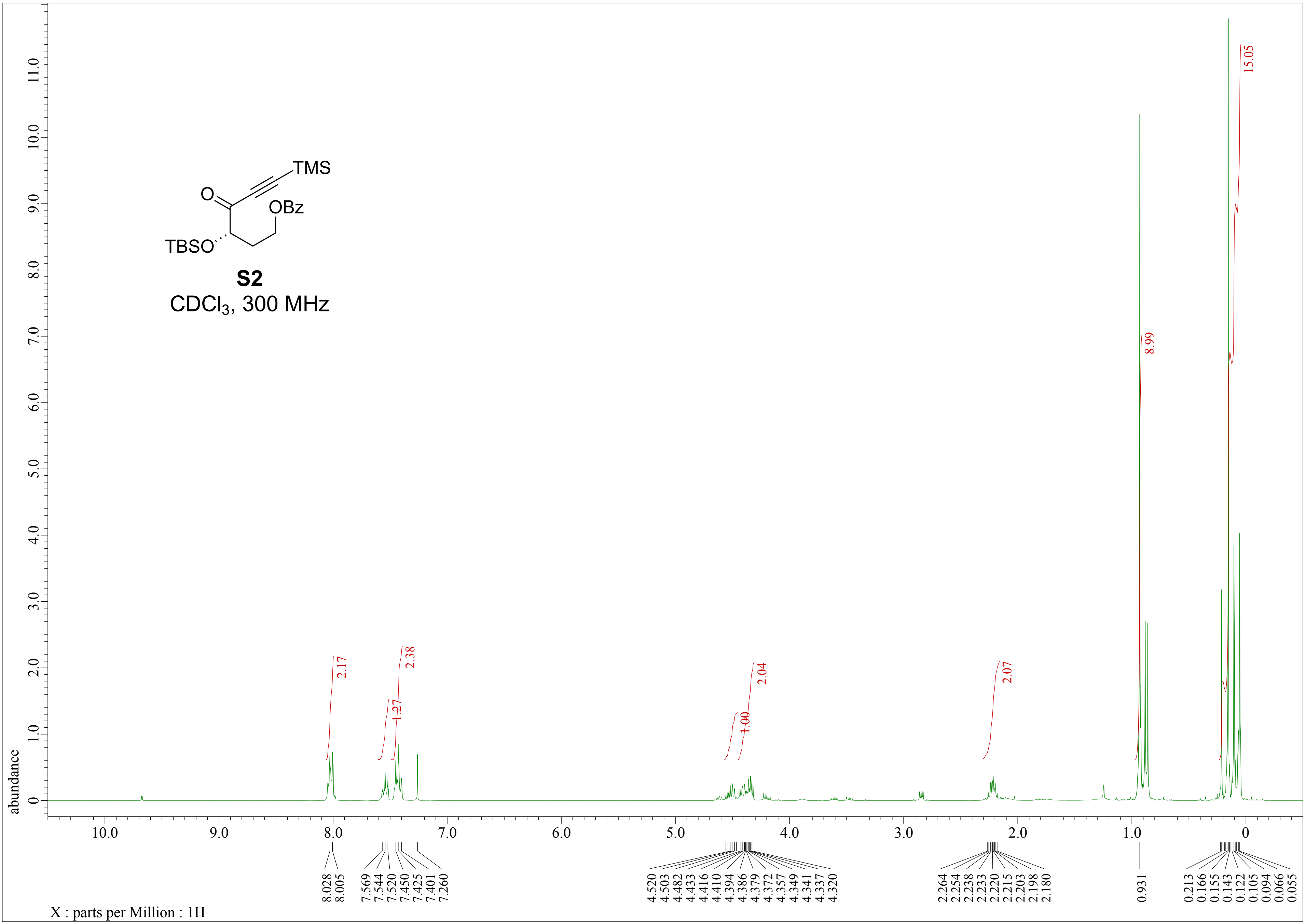
TMS
O
OBz
TBSO
S2
CDCl3, 300 MHz
2.17
1.27
2.38
1.00
2.04
2.07
8.99
15.05
8.028 8.005
7.569 7.544 7.520 7.450 7.425 7.401 7.260
4.520 4.503 4.482 4.433 4.416 4.410 4.394 4.386 4.379 4.372 4.357 4.349 4.341 4.337 4.320
2.264 2.254 2.238 2.233 2.220 2.215 2.203 2.198 2.180
0.931
0.213 0.166 0.155 0.143 0.122 0.105 0.094 0.066 0.055
abundance
X : parts per Million : 1H

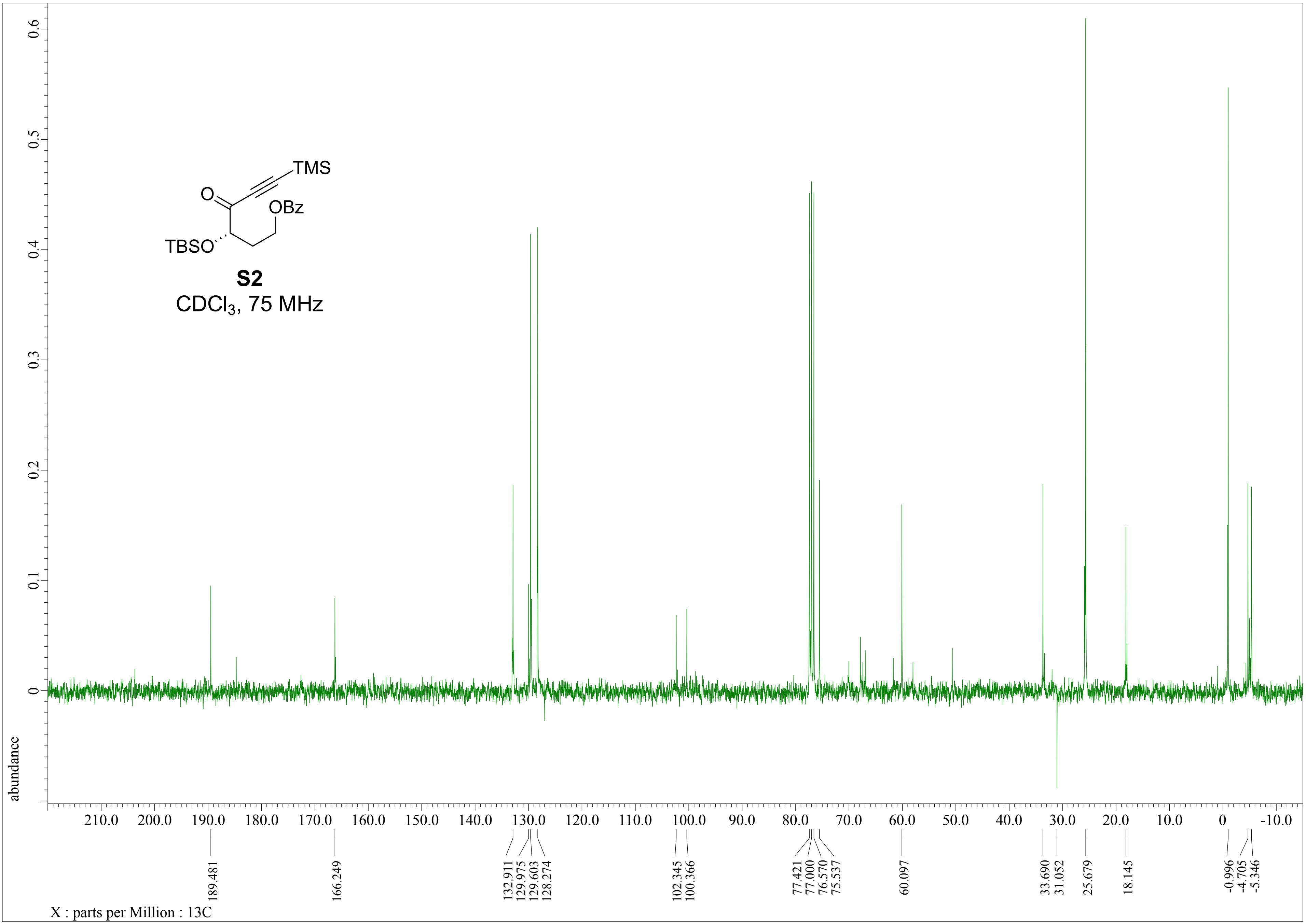
TMS
O
OBz
TBSO
S2
CDCl3, 75 MHz
abundance
X : parts per Million : 13C
189.481
166.249
132.911
129.975
129.603
128.274
102.345
100.366
77.421
77.000
76.570
75.537
60.097
33.690
31.052
25.679
18.145
-0.996
-4.705
-5.346

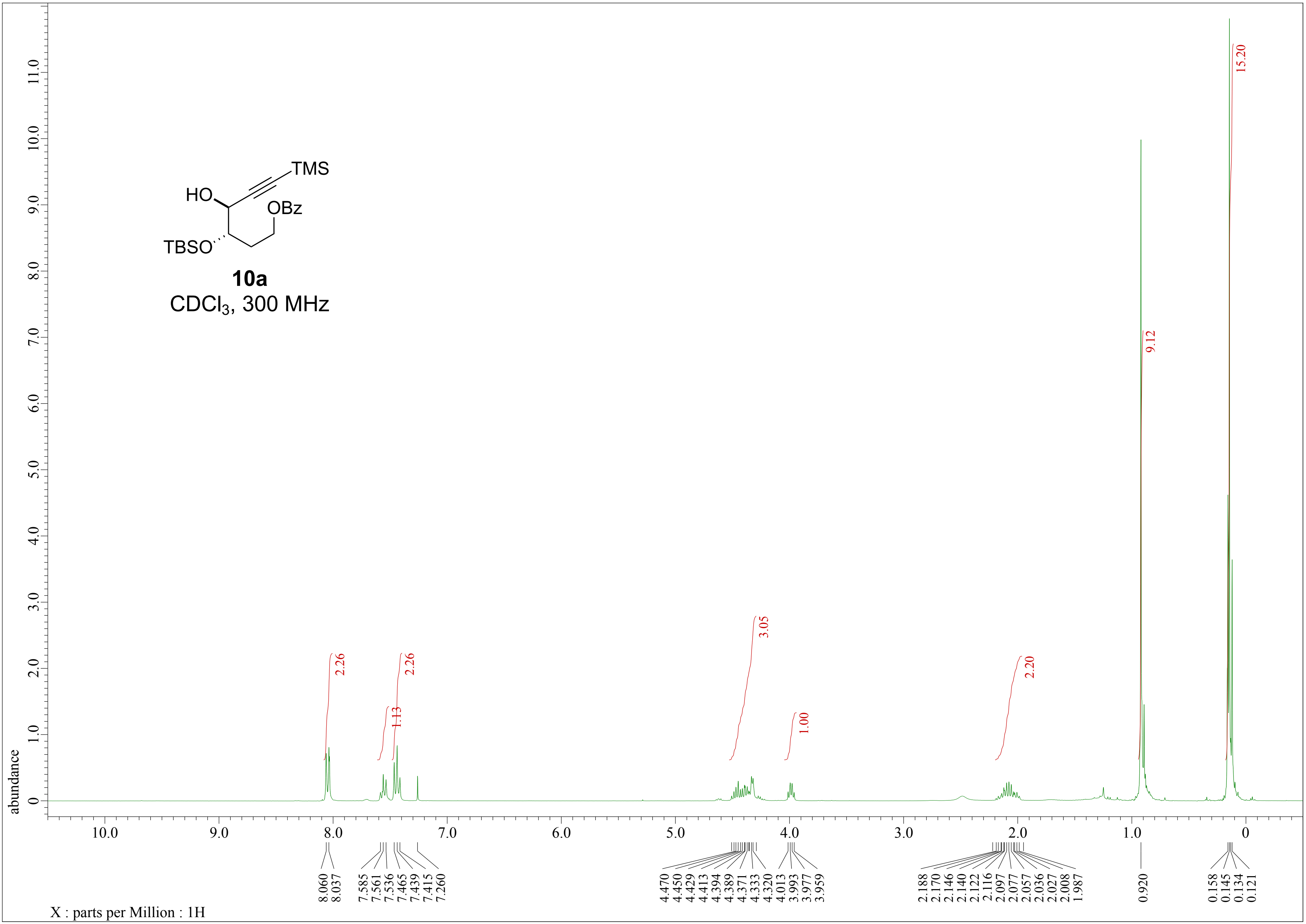
TMS
HO
OBz
TBSO
10a
CDCl3, 300 MHz
abundance
X : parts per Million : 1H
2.26
1.13
2.26
3.05
1.00
2.20
9.12
15.20
8.060
8.037
7.585
7.561
7.536
7.465
7.439
7.415
7.260
4.470
4.450
4.429
4.413
4.394
4.389
4.371
4.333
4.320
4.013
3.993
3.977
3.959
2.188
2.170
2.146
2.140
2.122
2.116
2.097
2.077
2.057
2.036
2.027
2.008
1.987
0.920
0.158
0.145
0.134
0.121

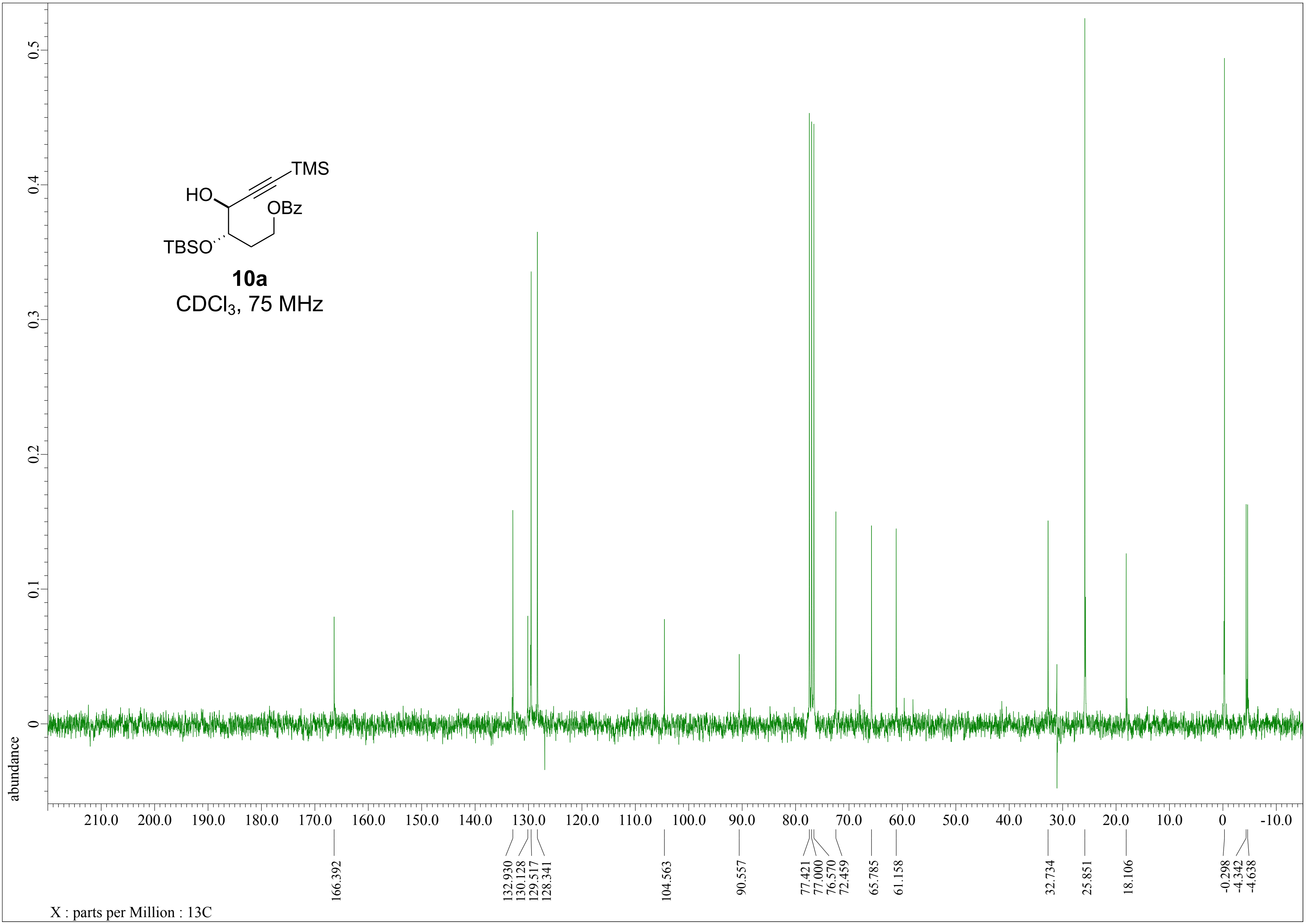
TMS
HO
OBz
TBSO
10a
CDCl3, 75 MHz
abundance
0.5
0.4
0.3
0.2
0.1
0
210.0 200.0 190.0 180.0 170.0 160.0 150.0 140.0 130.0 120.0 110.0 100.0 90.0 80.0 70.0 60.0 50.0 40.0 30.0 20.0 10.0 0 -10.0
166.392
132.930
130.128
129.517
128.341
104.563
90.557
77.421
77.000
76.570
72.459
65.785
61.158
32.734
25.851
18.106
-0.298
-4.342
-4.638
X : parts per Million : 13C

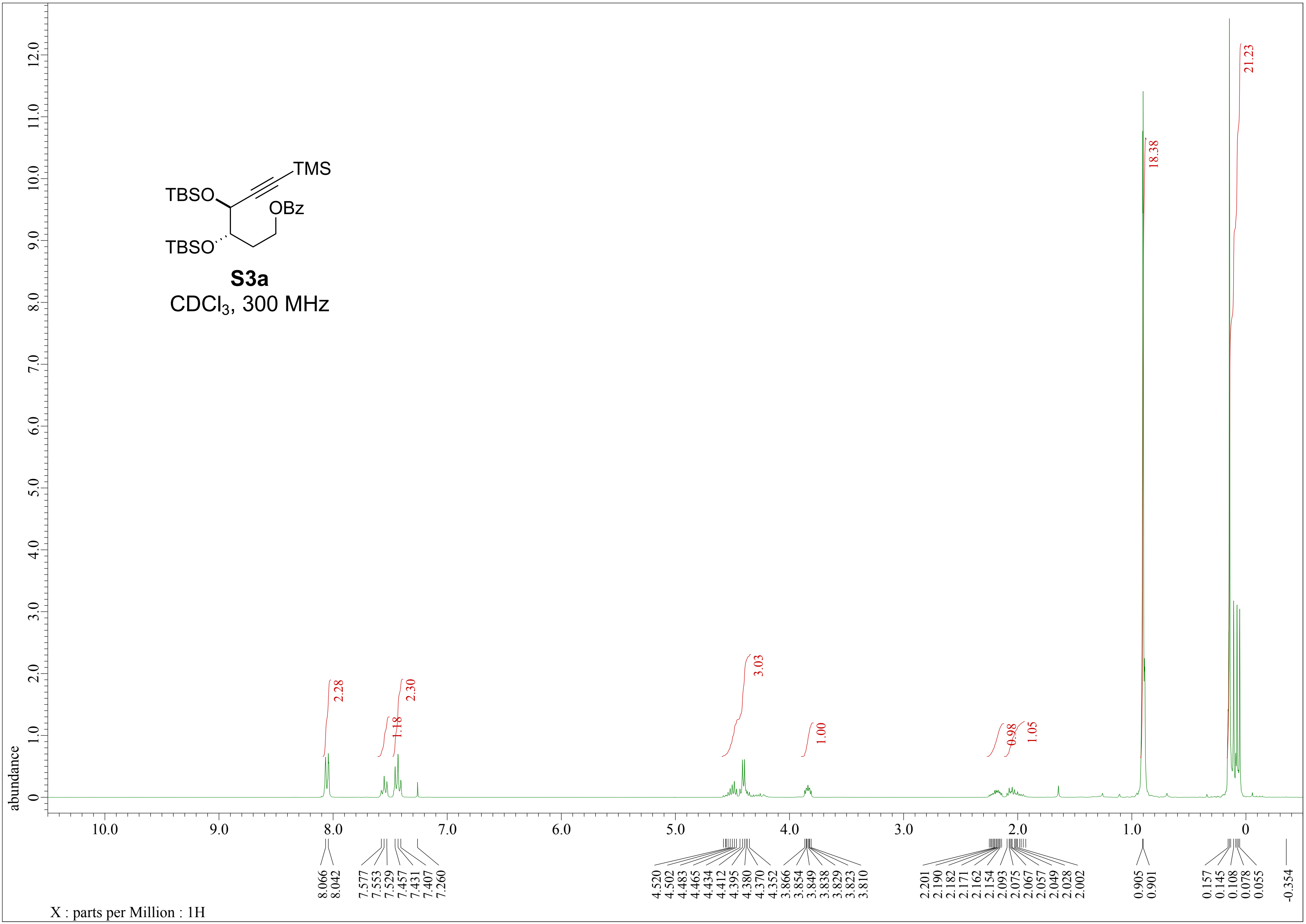
TMS
TBSO
OBz
TBSO
S3a
CDCl3, 300 MHz
abundance
X : parts per Million : 1H
2.28
1.18
2.30
3.03
1.00
0.98
1.05
18.38
21.23
8.066
8.042
7.577
7.553
7.529
7.457
7.431
7.407
7.260
4.520
4.502
4.483
4.465
4.434
4.412
4.395
4.380
4.370
4.352
3.866
3.854
3.849
3.838
3.829
3.823
3.810
2.201
2.190
2.182
2.171
2.162
2.154
2.093
2.075
2.067
2.057
2.049
2.028
2.002
0.905
0.901
0.157
0.145
0.108
0.078
0.055
-0.354

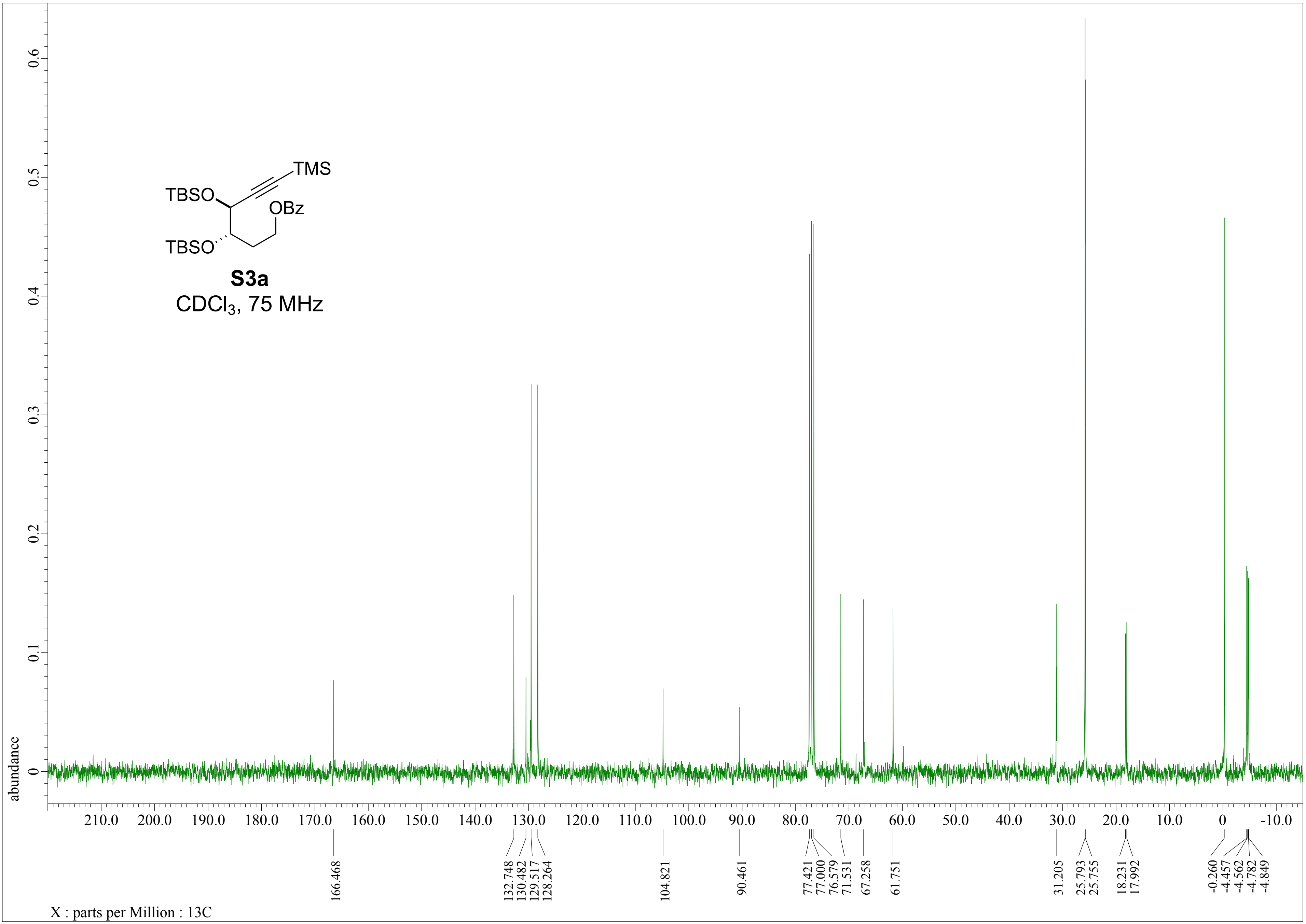
TMS
TBSO
OBz
TBSO
S3a
CDCl3, 75 MHz
abundance
0.6
0.5
0.4
0.3
0.2
0.1
0
210.0 200.0 190.0 180.0 170.0 160.0 150.0 140.0 130.0 120.0 110.0 100.0 90.0 80.0 70.0 60.0 50.0 40.0 30.0 20.0 10.0 0 -10.0
166.468
132.748
130.482
129.517
128.264
104.821
90.461
77.421
77.000
76.579
71.531
67.258
61.751
31.205
25.793
25.755
18.231
17.992
-0.260
-4.457
-4.562
-4.782
-4.849
X : parts per Million : 13C

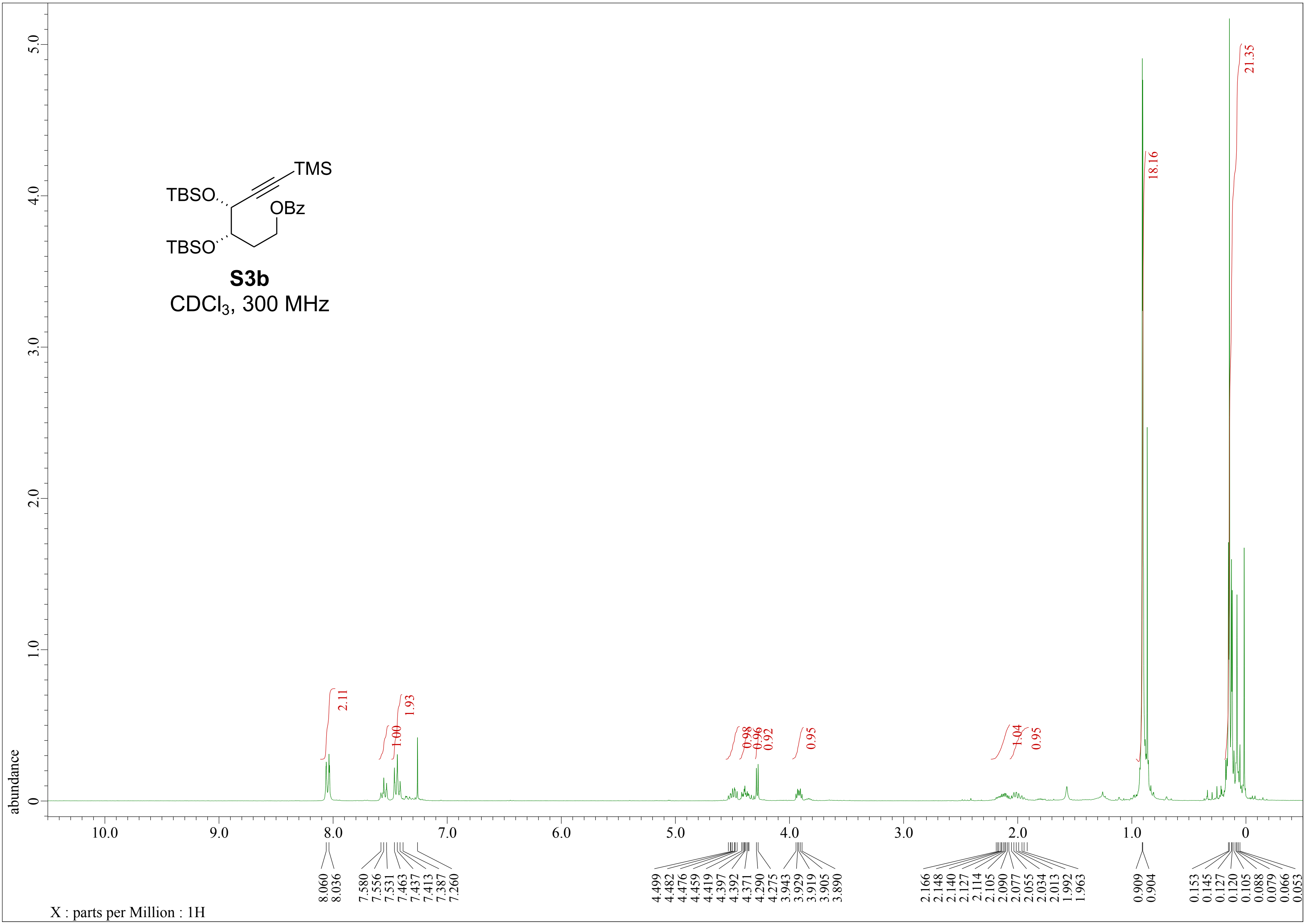
TMS
TBSO
OBz
TBSO
S3b
CDCl3, 300 MHz
abundance
X : parts per Million : 1H
2.11
1.00
1.93
0.98
0.96
0.92
0.95
1.04
0.95
18.16
21.35
8.060 8.036
7.580 7.556 7.531 7.463 7.437 7.413 7.387 7.260
4.499 4.482 4.476 4.459 4.419 4.397 4.392 4.371 4.290 4.275 3.943 3.929 3.919 3.905 3.890
2.166 2.148 2.140 2.127 2.114 2.105 2.090 2.077 2.055 2.034 2.013 1.992 1.963
0.909 0.904
0.153 0.145 0.127 0.120 0.105 0.088 0.079 0.066 0.053

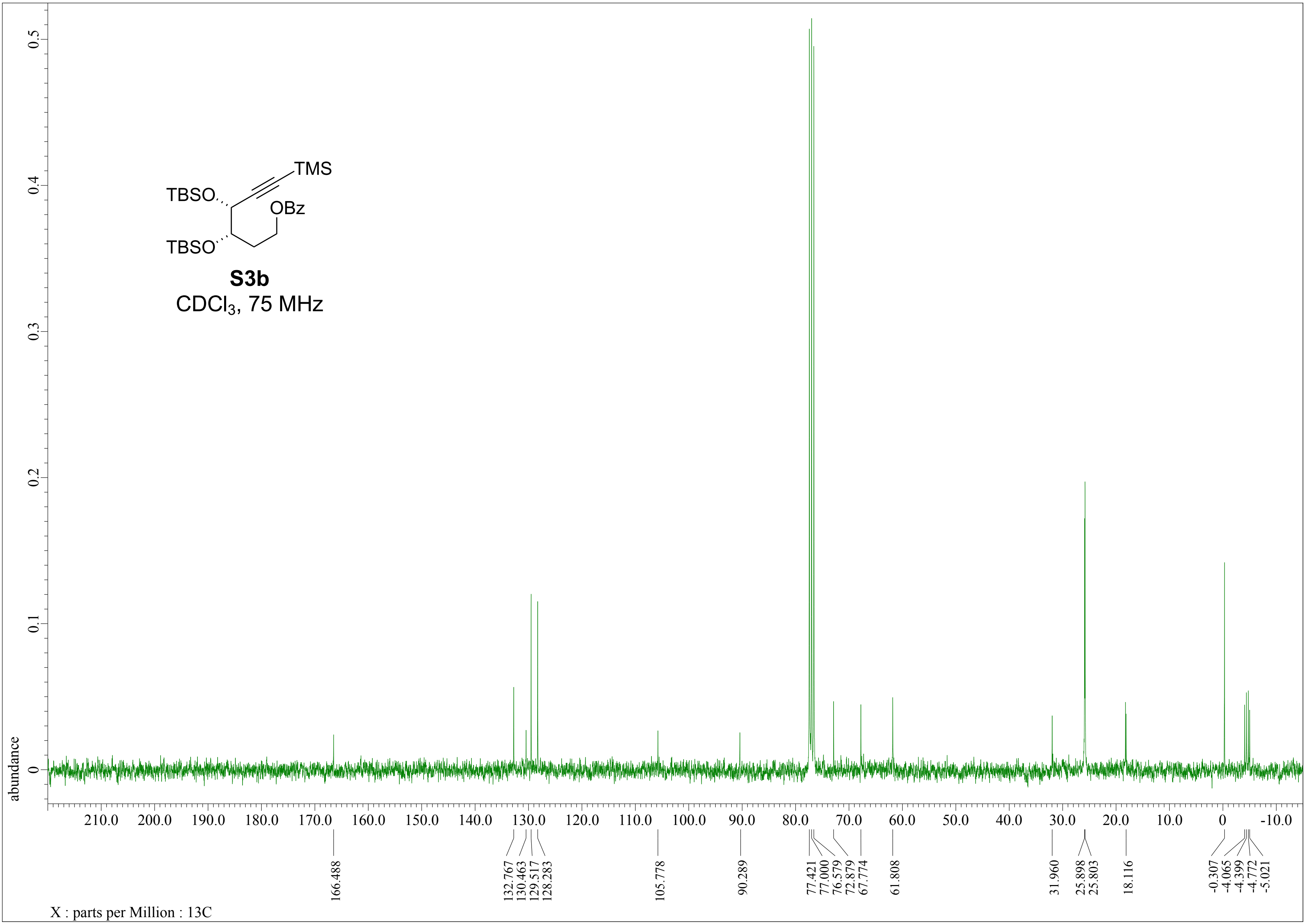

TMS
TBSO
OBz
TBSO
S3b
CDCl3, 75 MHz
abundance
0.5
0.4
0.3
0.2
0.1
0
210.0 200.0 190.0 180.0 170.0 160.0 150.0 140.0 130.0 120.0 110.0 100.0 90.0 80.0 70.0 60.0 50.0 40.0 30.0 20.0 10.0 0 -10.0
166.488
132.767
130.463
129.517
128.283
105.778
90.289
77.421
77.000
76.579
72.879
67.774
61.808
31.960
25.898
25.803
18.116
-0.307
-4.065
-4.399
-4.772
-5.021
X : parts per Million : 13C

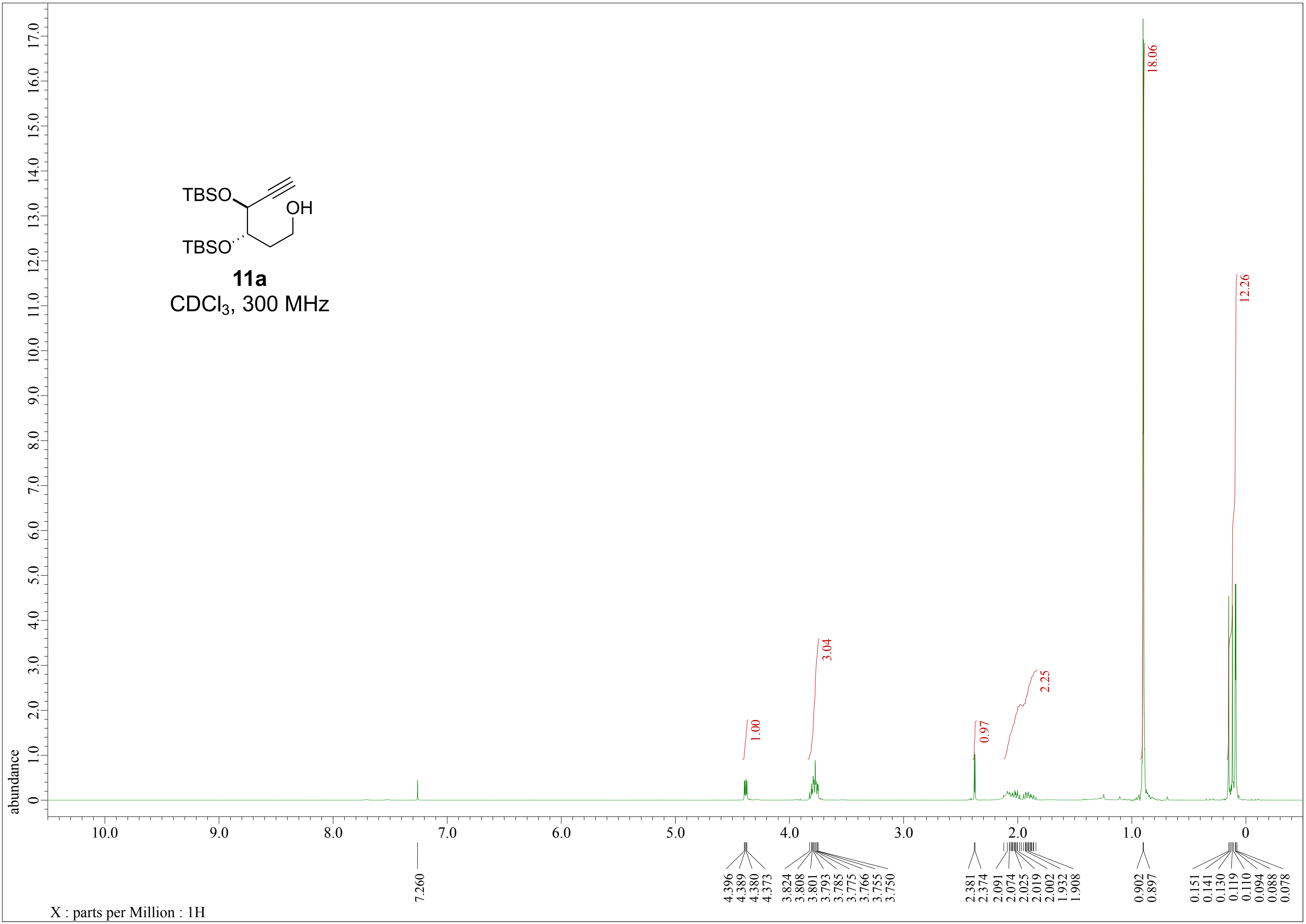
TBSO
TBSO
OH
11a
CDCl$_3$, 300 MHz
abundance
X : parts per Million : 1H
18.06
12.26
3.04
2.25
1.00
0.97
7.260
4.396 4.389 4.380 4.373
3.824 3.808 3.801 3.793 3.785 3.775 3.766 3.755 3.750
2.381 2.374
2.091 2.074 2.025 2.019 2.002 1.932 1.908
0.902 0.897
0.151 0.141 0.130 0.119 0.110 0.094 0.088 0.078

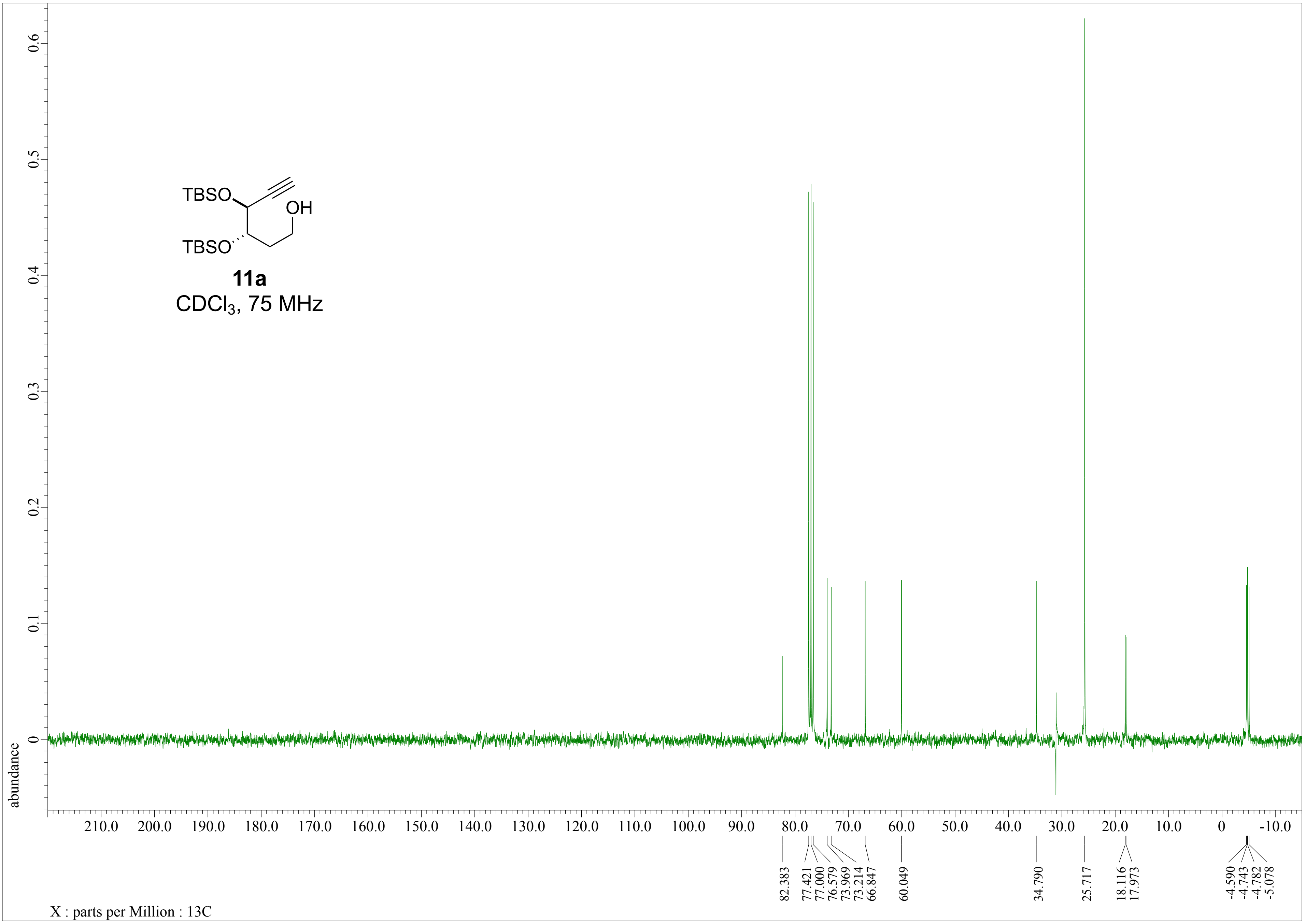
TBSO
TBSO
OH
11a
CDCl$_3$, 75 MHz
abundance
0.6
0.5
0.4
0.3
0.2
0.1
0
210.0 200.0 190.0 180.0 170.0 160.0 150.0 140.0 130.0 120.0 110.0 100.0 90.0 80.0 70.0 60.0 50.0 40.0 30.0 20.0 10.0 0 -10.0
82.383
77.421
77.000
76.579
73.969
73.214
66.847
60.049
34.790
25.717
18.116
17.973
-4.590
-4.743
-4.782
-5.078
X : parts per Million : 13C

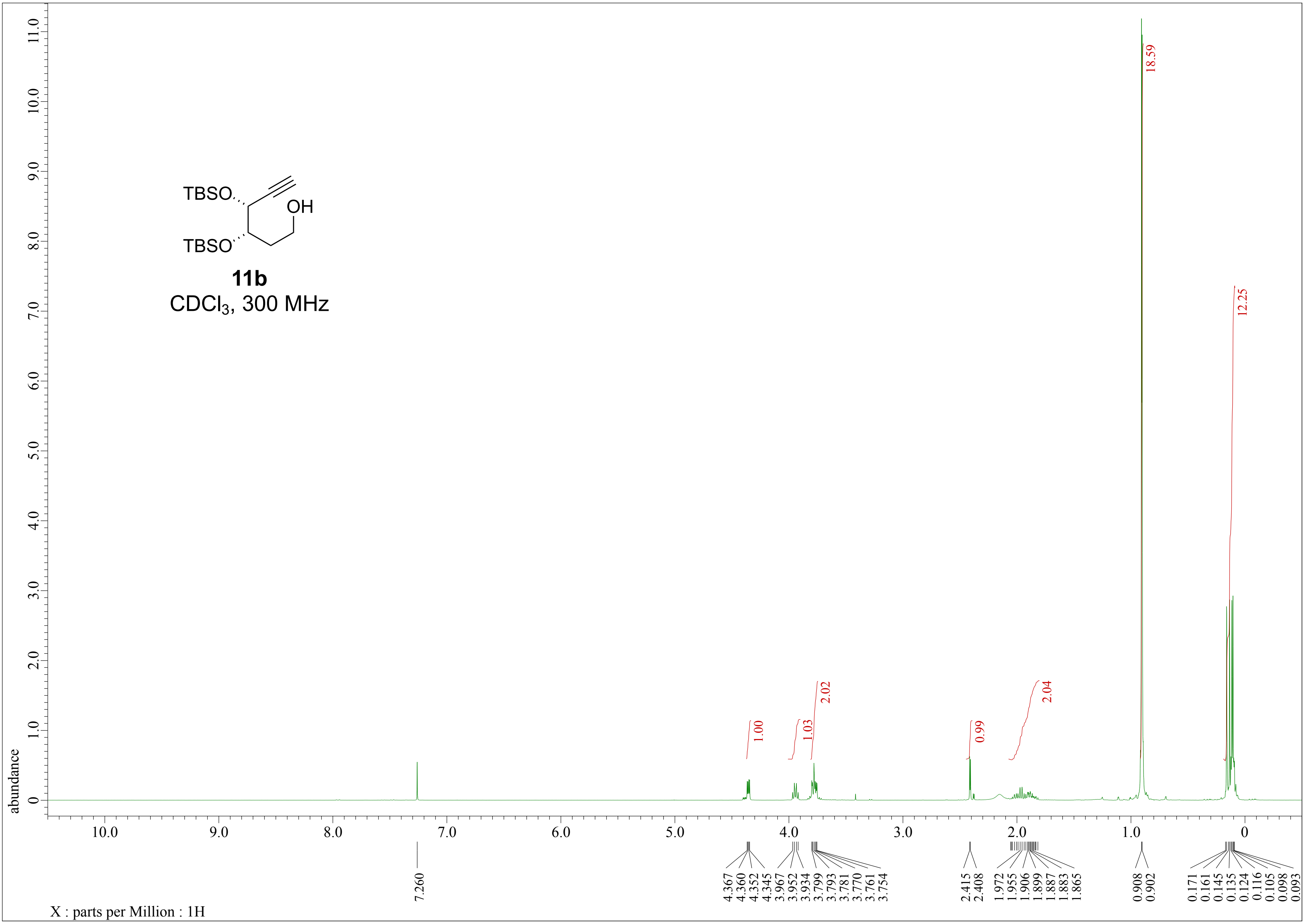

TBSO
TBSO
OH
11b
CDCl3, 300 MHz
abundance
X : parts per Million : 1H
18.59
12.25
1.00
1.03
2.02
0.99
2.04
7.260
4.367 4.360 4.352 4.345 3.967 3.952 3.934 3.799 3.793 3.781 3.770 3.761 3.754
2.415 2.408
1.972 1.955 1.906 1.899 1.887 1.883 1.865
0.908 0.902
0.171 0.161 0.145 0.135 0.124 0.116 0.105 0.098 0.093

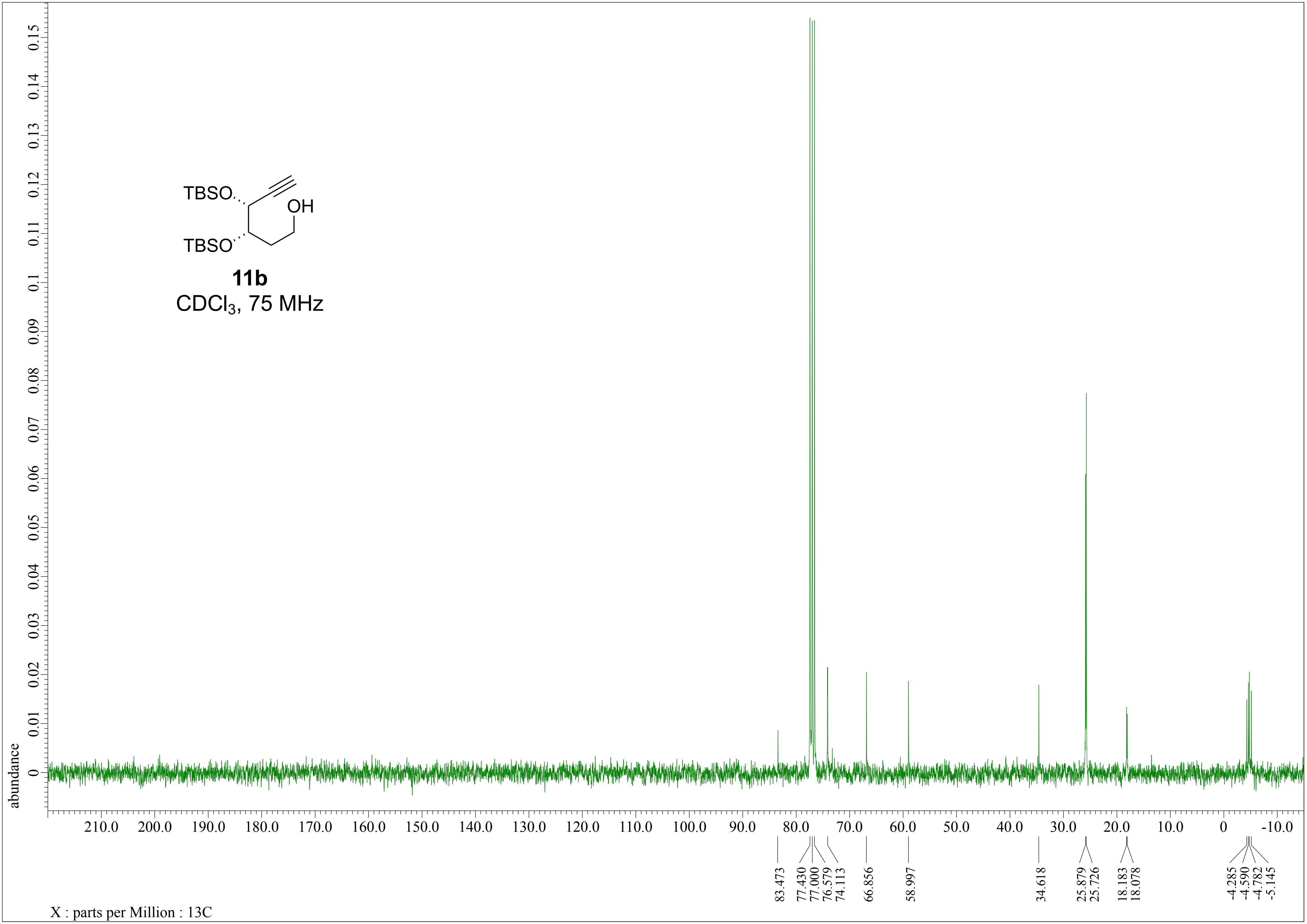

TBSO
TBSO
OH
11b
CDCl3, 75 MHz
abundance
X : parts per Million : 13C
83.473
77.430
77.000
76.579
74.113
66.856
58.997
34.618
25.879
25.726
18.183
18.078
-4.285
-4.590
-4.782
-5.145

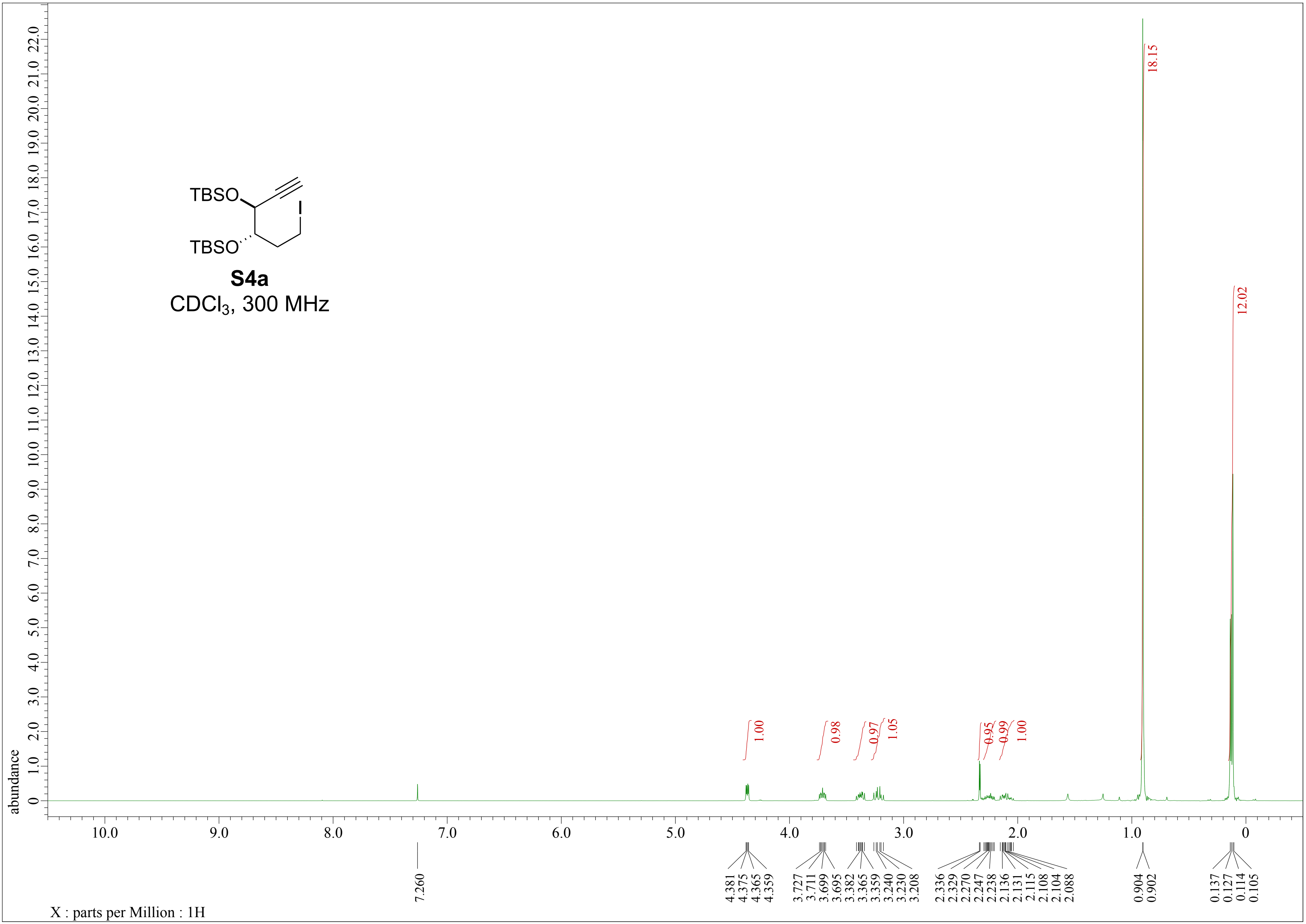
TBSO
TBSO
I
S4a
CDCl3, 300 MHz
abundance
X : parts per Million : 1H
1.00
0.98
0.97
1.05
0.95
0.99
1.00
18.15
12.02
7.260
4.381
4.375
4.365
4.359
3.727
3.711
3.699
3.695
3.382
3.365
3.359
3.240
3.230
3.208
2.336
2.329
2.270
2.247
2.238
2.136
2.131
2.115
2.108
2.104
2.088
0.904
0.902
0.137
0.127
0.114
0.105

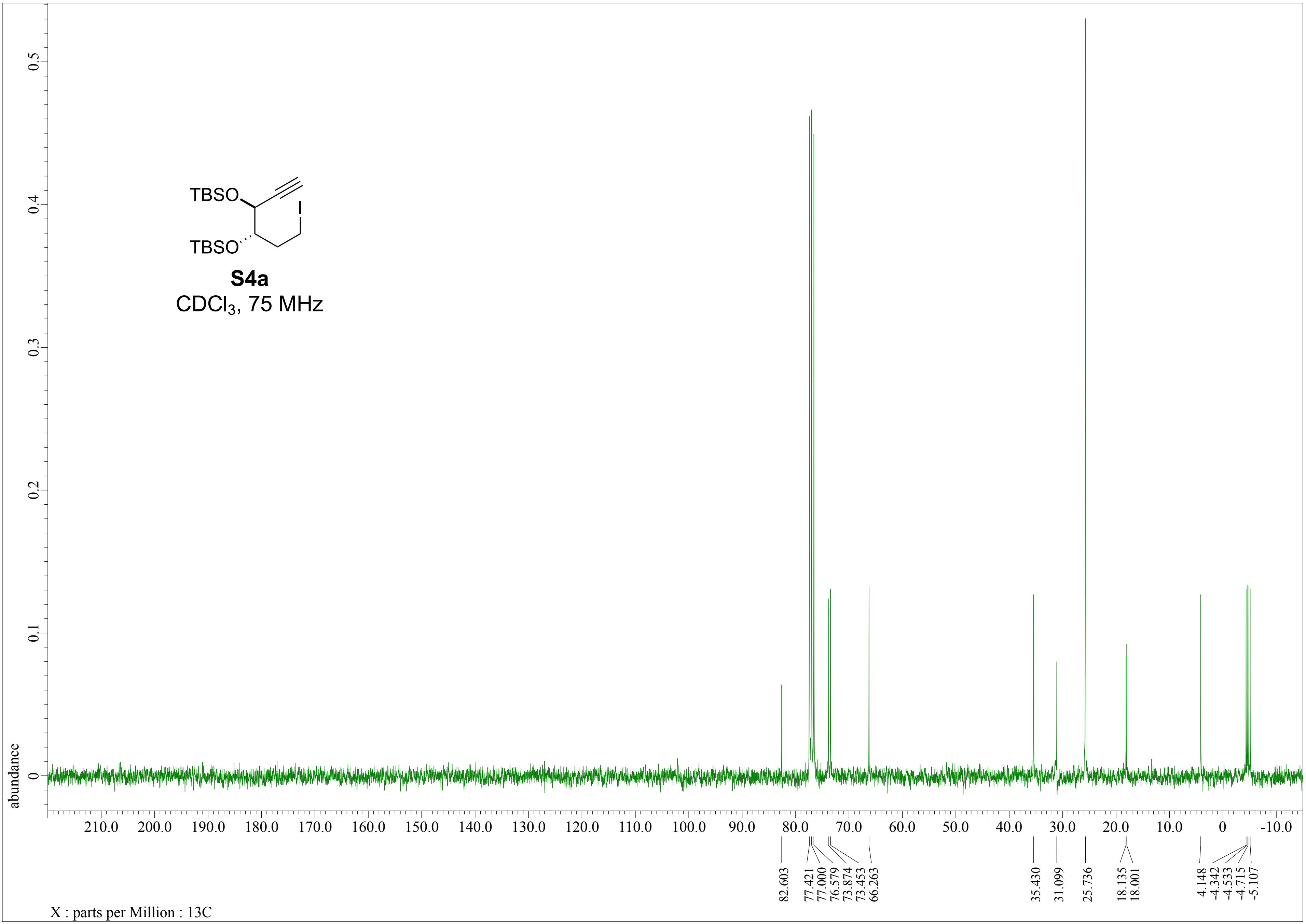
TBSO
TBSO
I
S4a
CDCl3, 75 MHz
abundance
0.5
0.4
0.3
0.2
0.1
0
210.0 200.0 190.0 180.0 170.0 160.0 150.0 140.0 130.0 120.0 110.0 100.0 90.0 80.0 70.0 60.0 50.0 40.0 30.0 20.0 10.0 0 -10.0
82.603
77.421
77.000
76.579
73.874
73.453
66.263
35.430
31.099
25.736
18.135
18.001
4.148
-4.342
-4.533
-4.715
-5.107
X : parts per Million : 13C

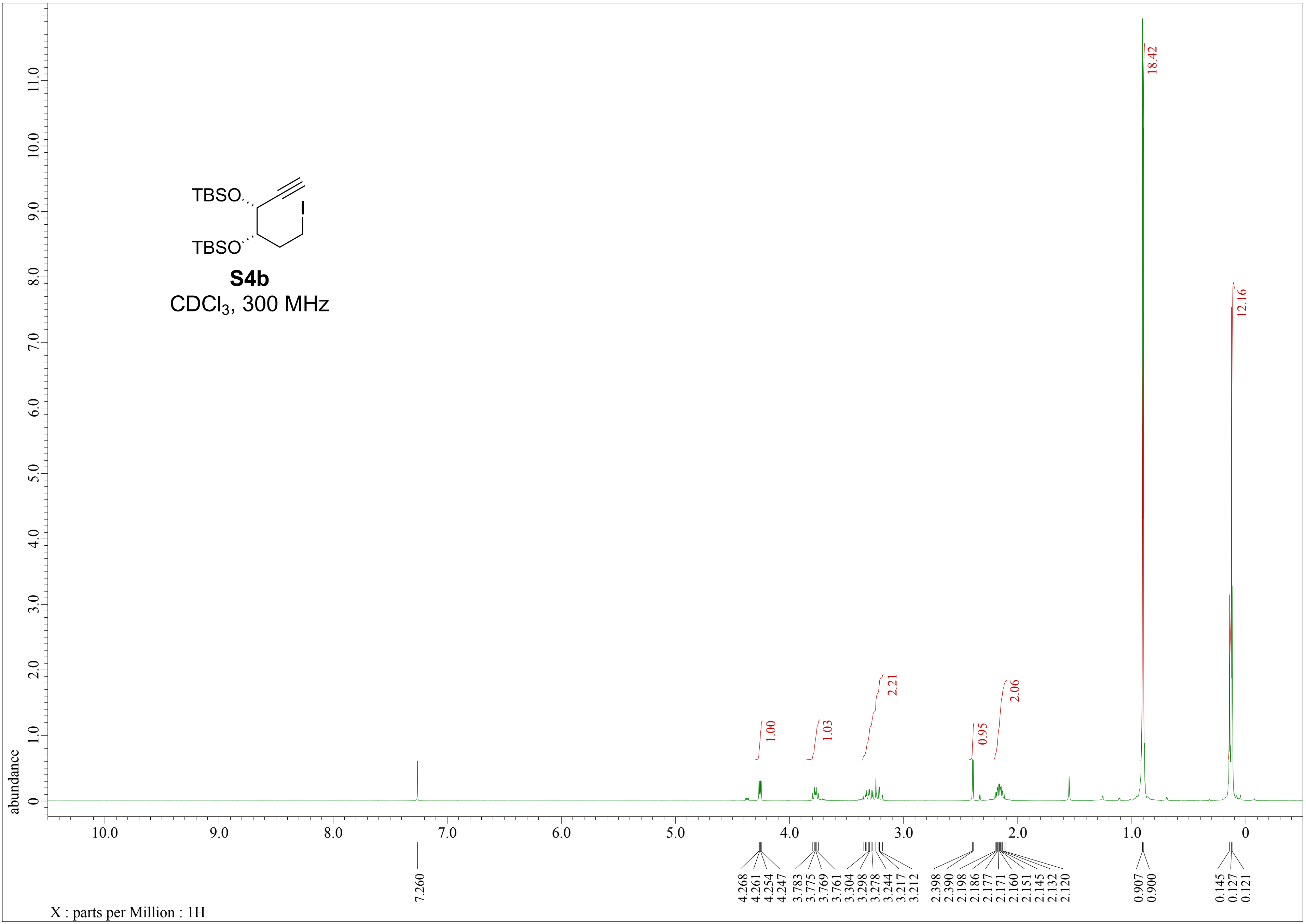

TBSO
TBSO
I
S4b
CDCl3, 300 MHz
abundance
X : parts per Million : 1H
18.42
12.16
1.00
1.03
2.21
0.95
2.06
7.260
4.268 4.261 4.254 4.247
3.783 3.775 3.769 3.761
3.304 3.298 3.278 3.244 3.217 3.212
2.398 2.390 2.198 2.186 2.177 2.171 2.160 2.151 2.145 2.132 2.120
0.907 0.900
0.145 0.127 0.121

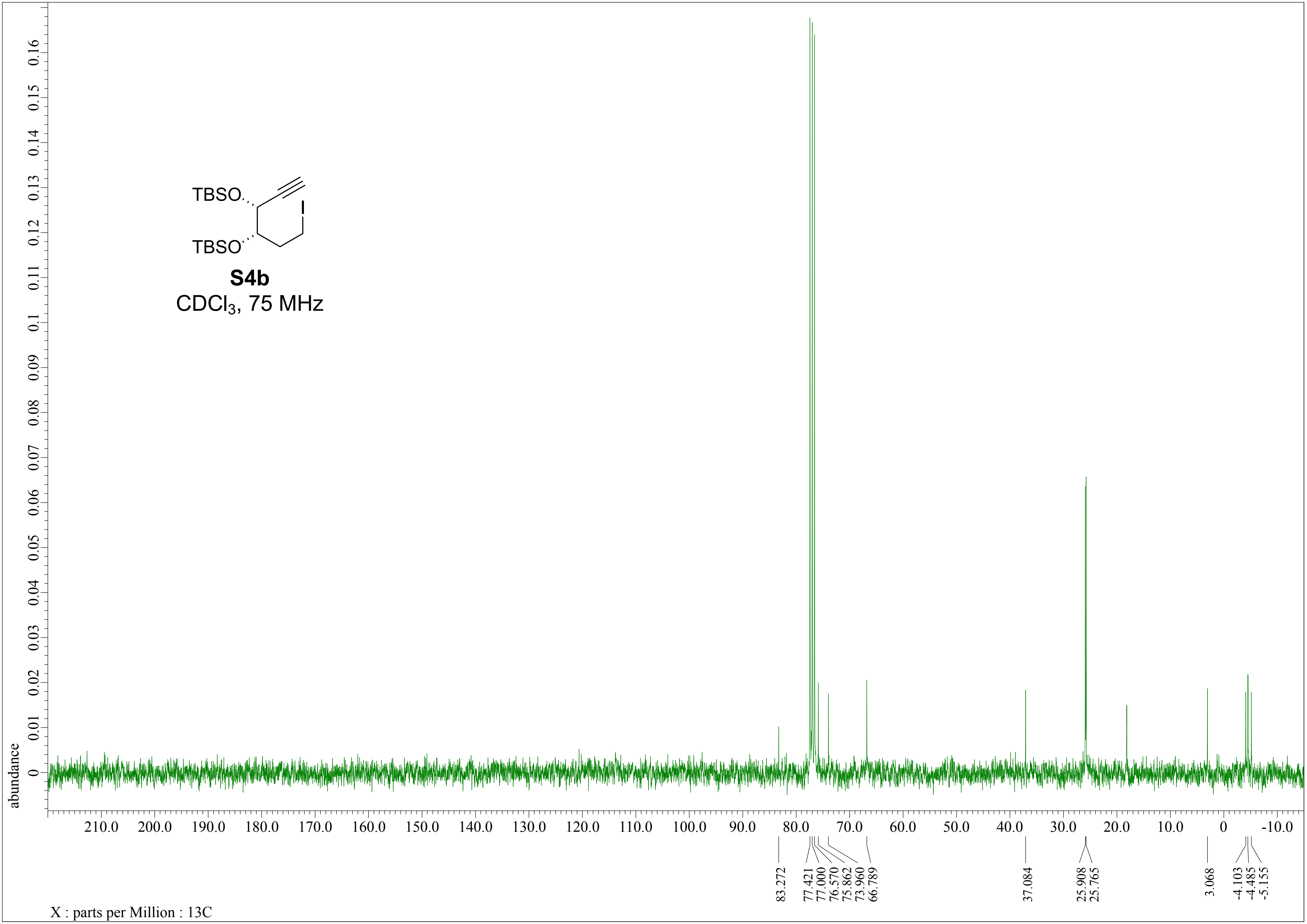

S4b

$CDCl_3$, 75 MHz

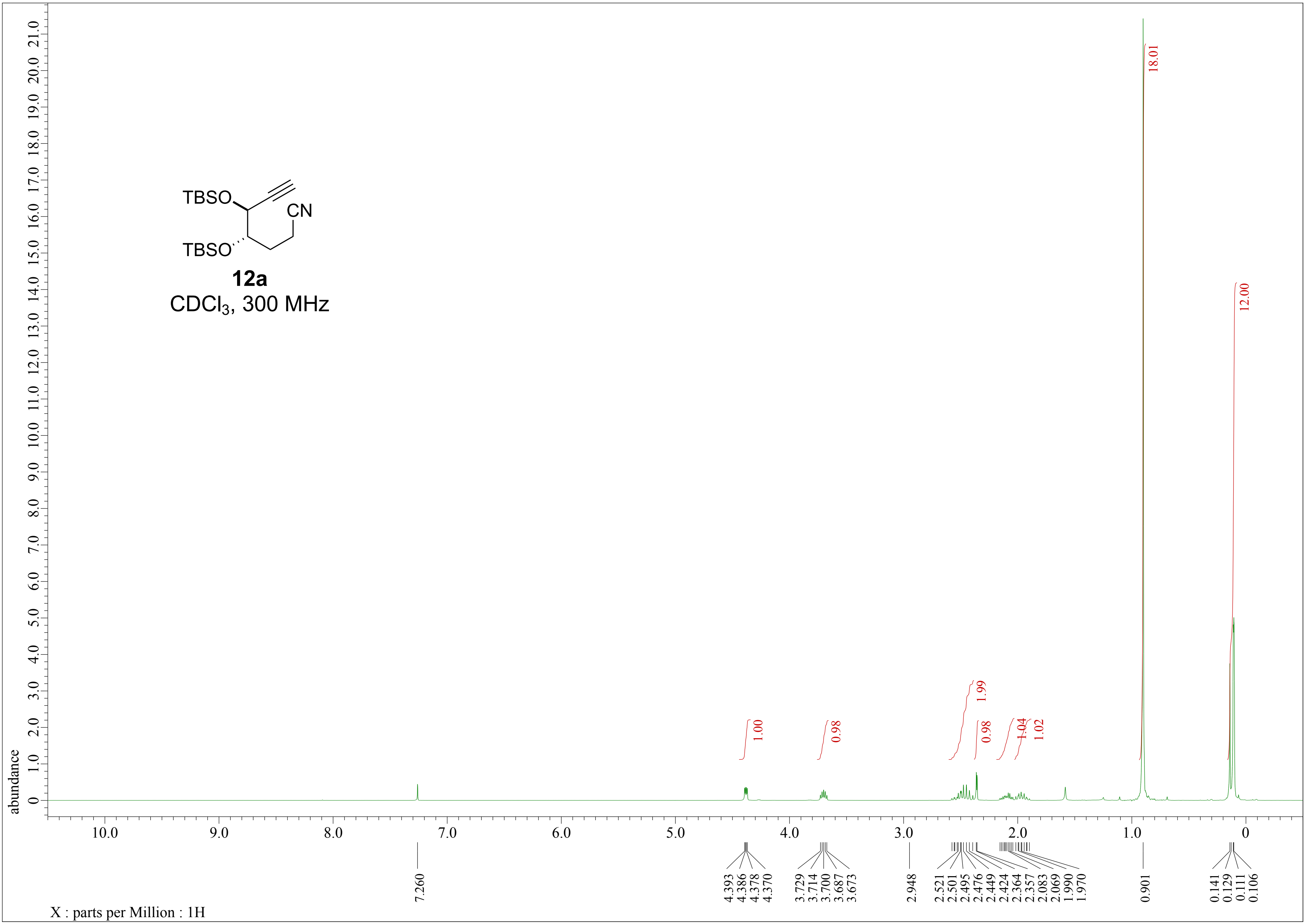
TBSO
TBSO
CN
12a
CDCl3, 300 MHz
abundance
X : parts per Million : 1H
10.0 9.0 8.0 7.0 6.0 5.0 4.0 3.0 2.0 1.0 0
18.01
12.00
1.00
0.98
1.99
0.98
1.04
1.02
7.260
4.393 4.386 4.378 4.370
3.729 3.714 3.700 3.687 3.673
2.948
2.521 2.501 2.495 2.476 2.449 2.424 2.364 2.357 2.083 2.069 1.990 1.970
0.901
0.141 0.129 0.111 0.106

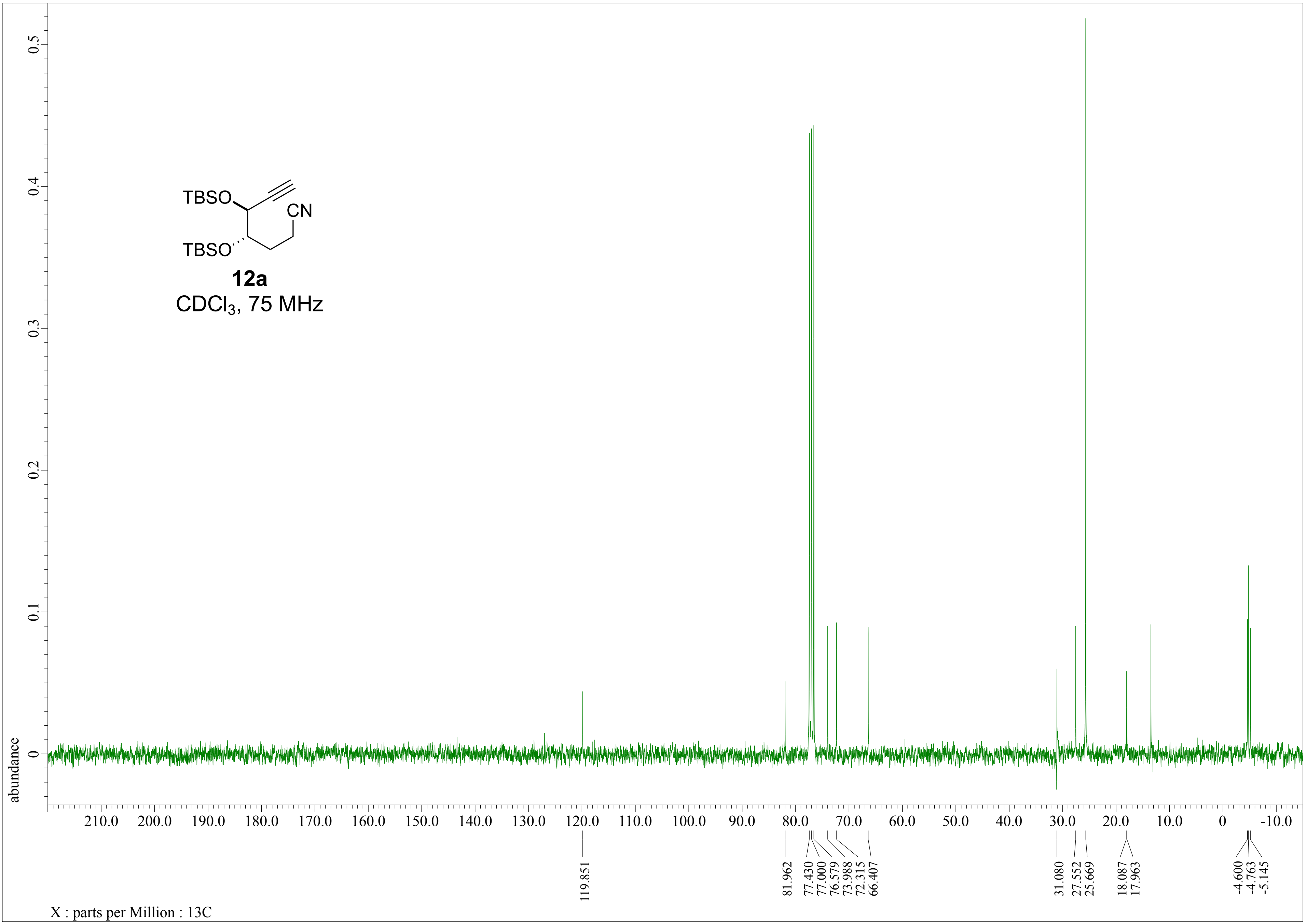
TBSO
TBSO
CN
12a
CDCl3, 75 MHz
abundance
X : parts per Million : 13C
119.851
81.962
77.430
77.000
76.579
73.988
72.315
66.407
31.080
27.552
25.669
18.087
17.963
-4.600
-4.763
-5.145

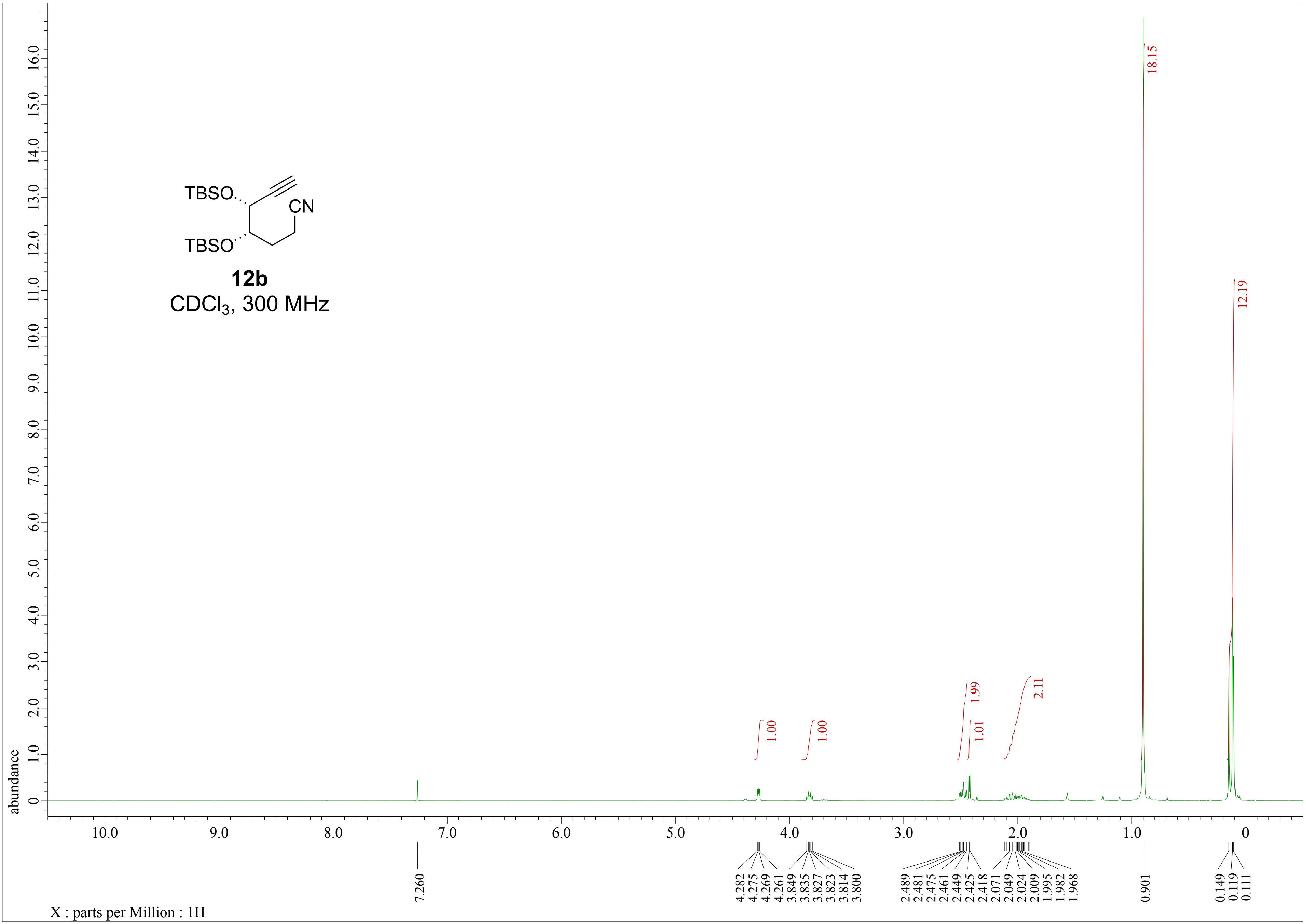
TBSO
TBSO
CN
12b
CDCl3, 300 MHz
abundance
X : parts per Million : 1H
7.260
4.282
4.275
4.269
4.261
3.849
3.835
3.827
3.823
3.814
3.800
2.489
2.481
2.475
2.461
2.449
2.425
2.418
2.071
2.049
2.024
2.009
1.995
1.982
1.968
0.901
0.149
0.119
0.111
1.00
1.00
1.99
1.01
2.11
18.15
12.19

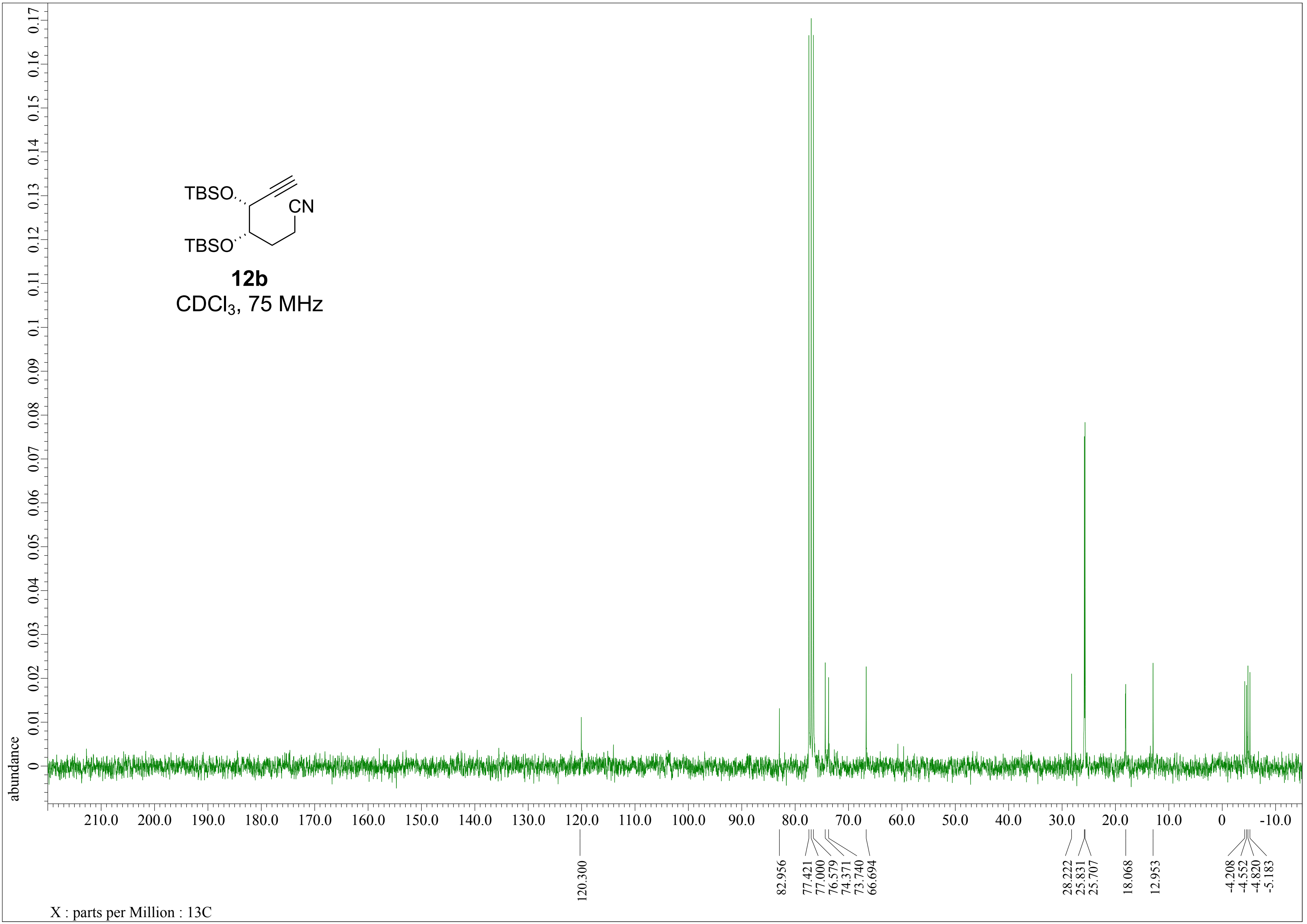
TBSO
TBSO
CN
12b
CDCl3, 75 MHz
abundance
X : parts per Million : 13C
120.300
82.956
77.421
77.000
76.579
74.371
73.740
66.694
28.222
25.831
25.707
18.068
12.953
-4.208
-4.552
-4.820
-5.183

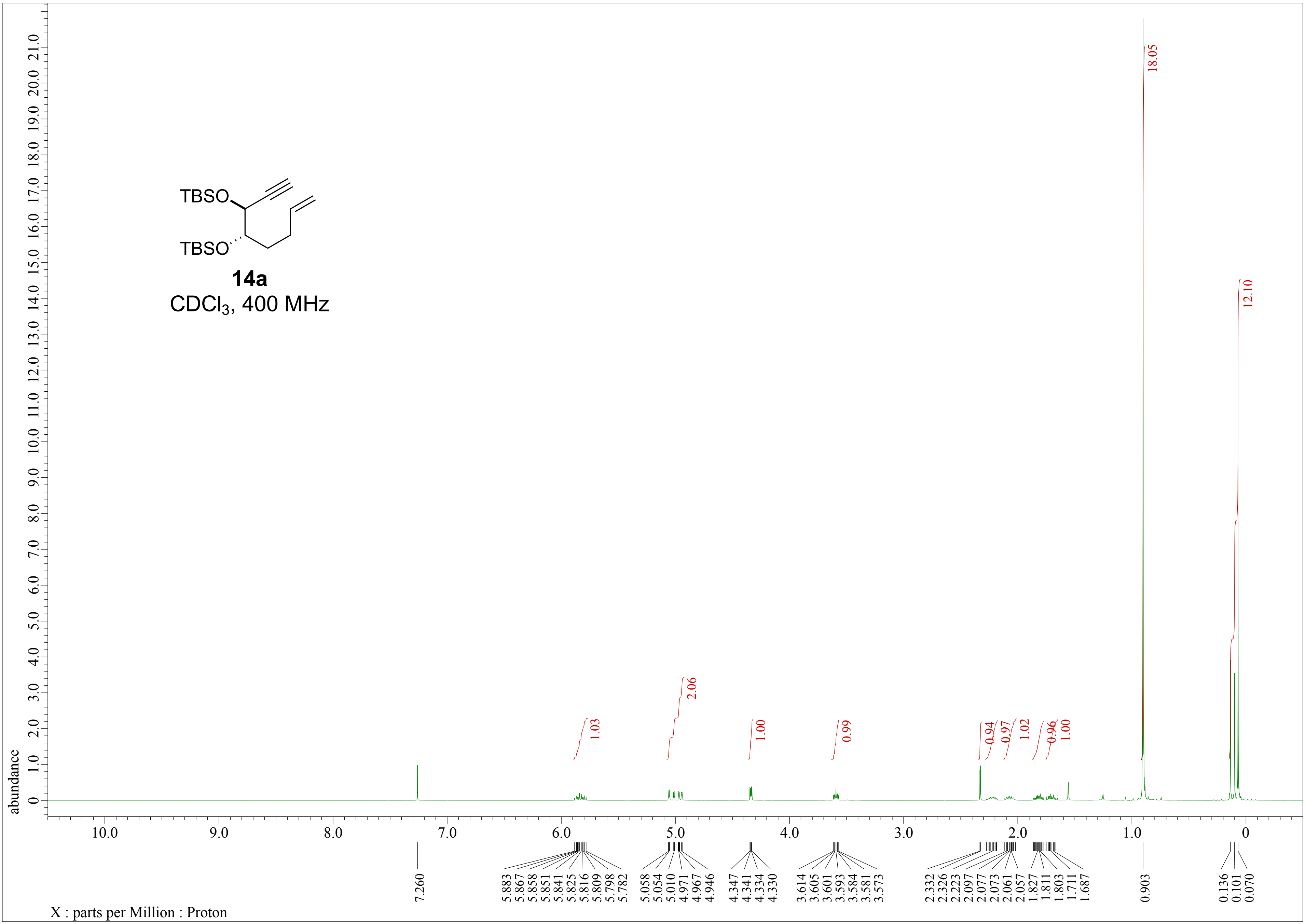

TBSO
TBSO
14a
CDCl3, 400 MHz
abundance
X : parts per Million : Proton

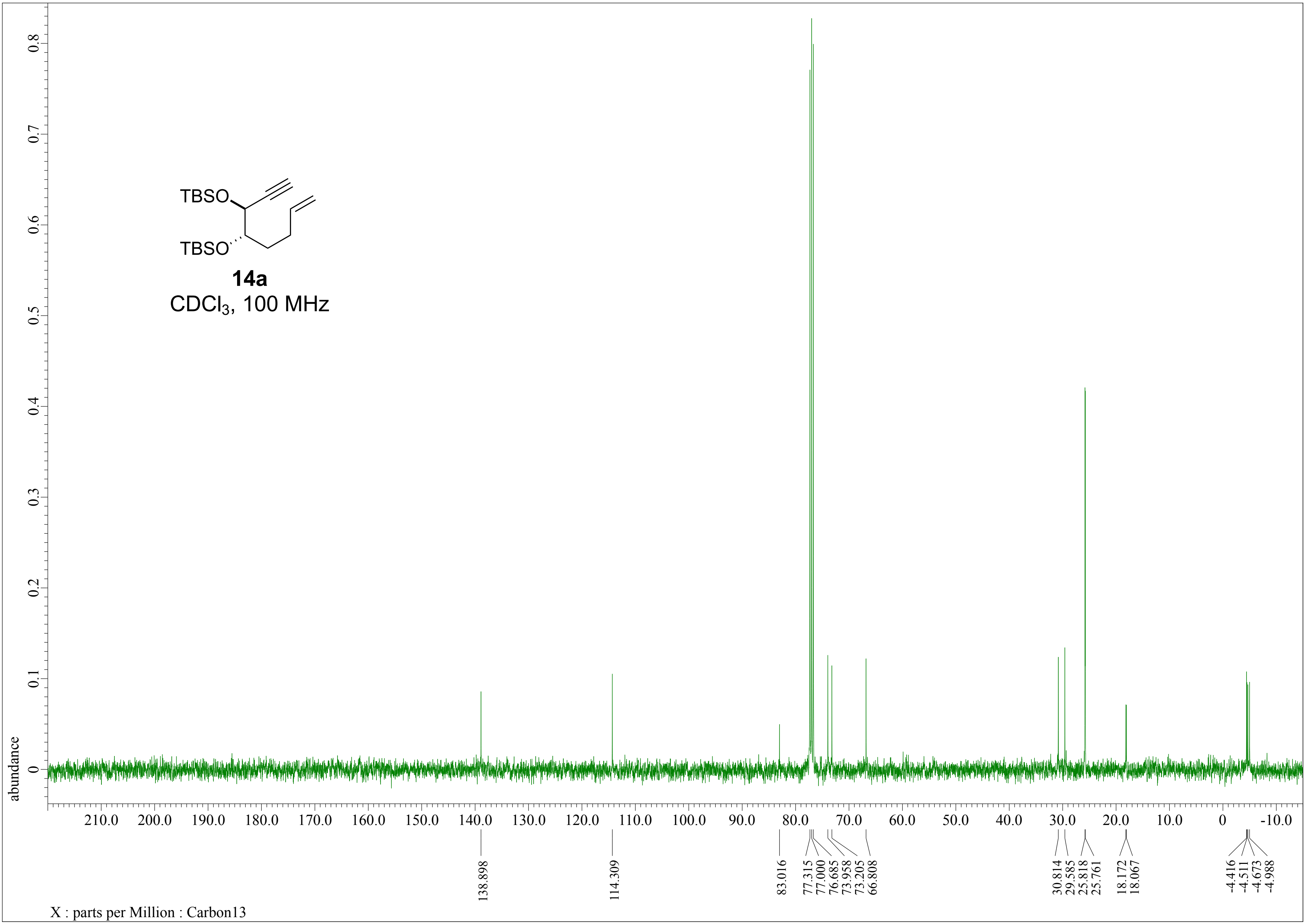
TBSO
TBSO
14a
CDCl3, 100 MHz
abundance
X : parts per Million : Carbon13
138.898
114.309
83.016
77.315
77.000
76.685
73.958
73.205
66.808
30.814
29.585
25.818
25.761
18.172
18.067
-4.416
-4.511
-4.673
-4.988

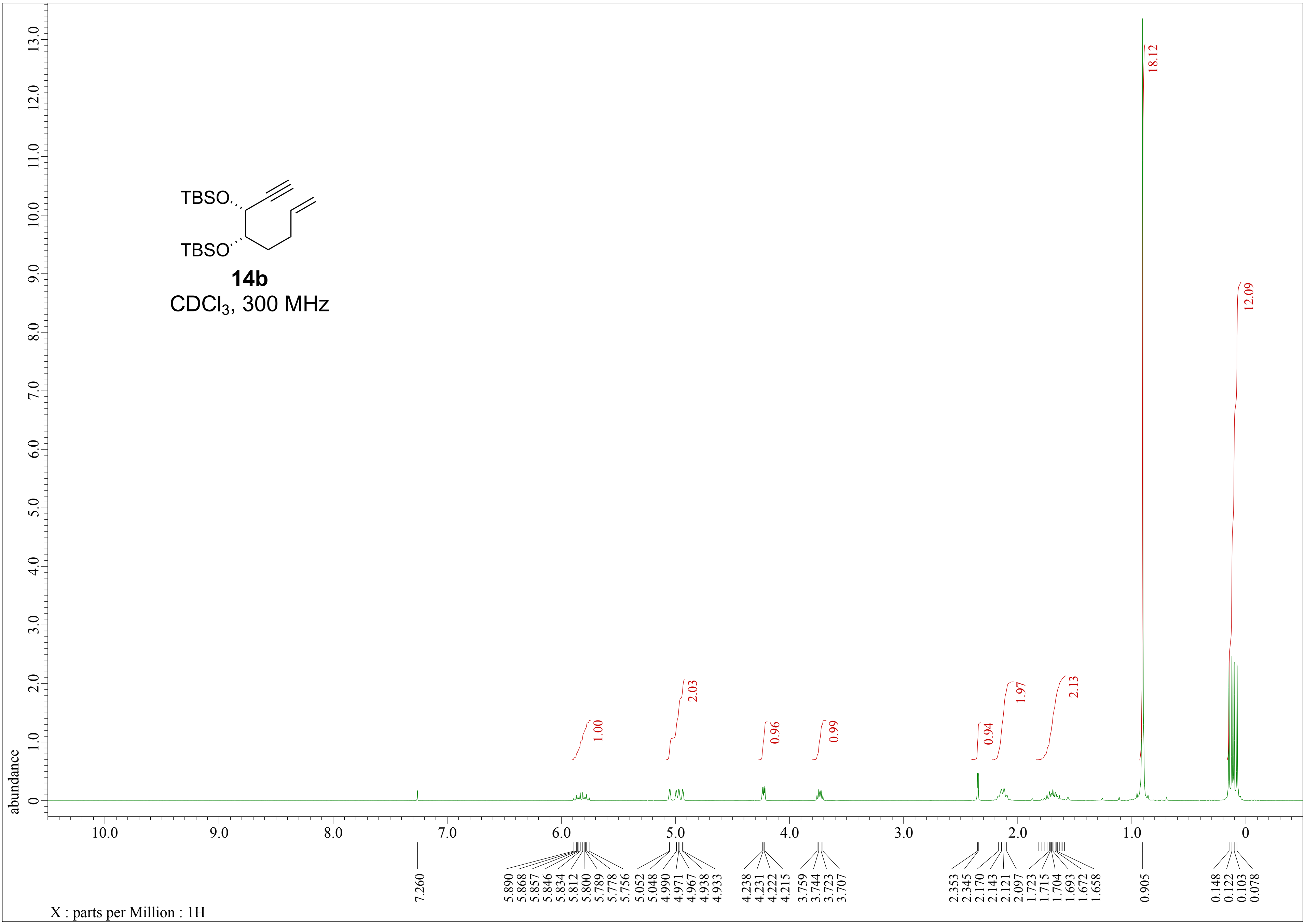
TBSO
TBSO
14b
CDCl3, 300 MHz
abundance
X : parts per Million : 1H
1.00
2.03
0.96
0.99
0.94
1.97
2.13
18.12
12.09
7.260
5.890 5.868 5.857 5.846 5.834 5.812 5.800 5.789 5.778 5.756
5.052 5.048 4.990 4.971 4.967 4.938 4.933
4.238 4.231 4.222 4.215
3.759 3.744 3.723 3.707
2.353 2.345 2.170 2.143 2.121 2.097
1.723 1.715 1.704 1.693 1.672 1.658
0.905
0.148 0.122 0.103 0.078

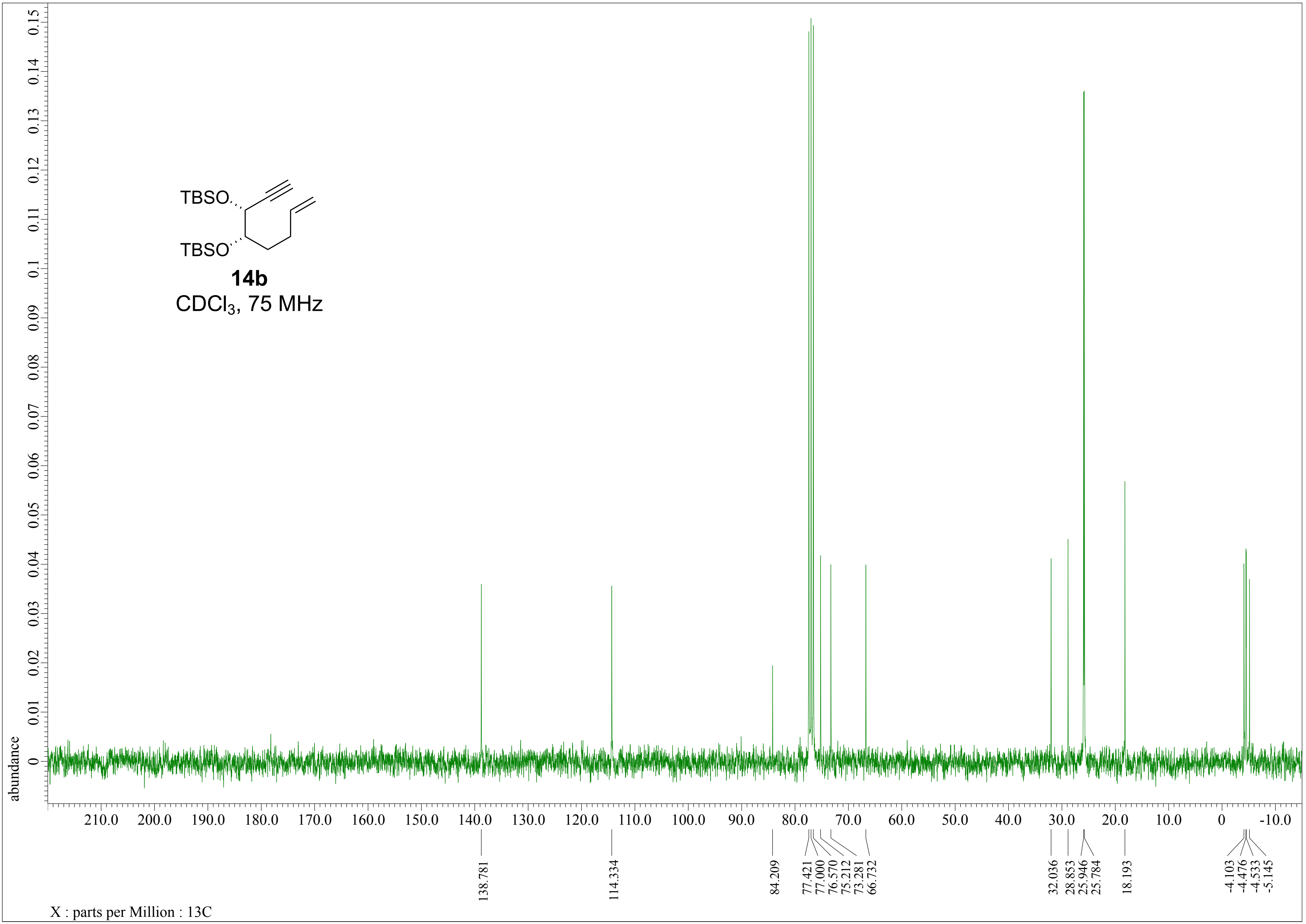
TBSO
TBSO
14b
CDCl3, 75 MHz
abundance
X : parts per Million : 13C
138.781
114.334
84.209
77.421
77.000
76.570
75.212
73.281
66.732
32.036
28.853
25.946
25.784
18.193
-4.103
-4.476
-4.533
-5.145

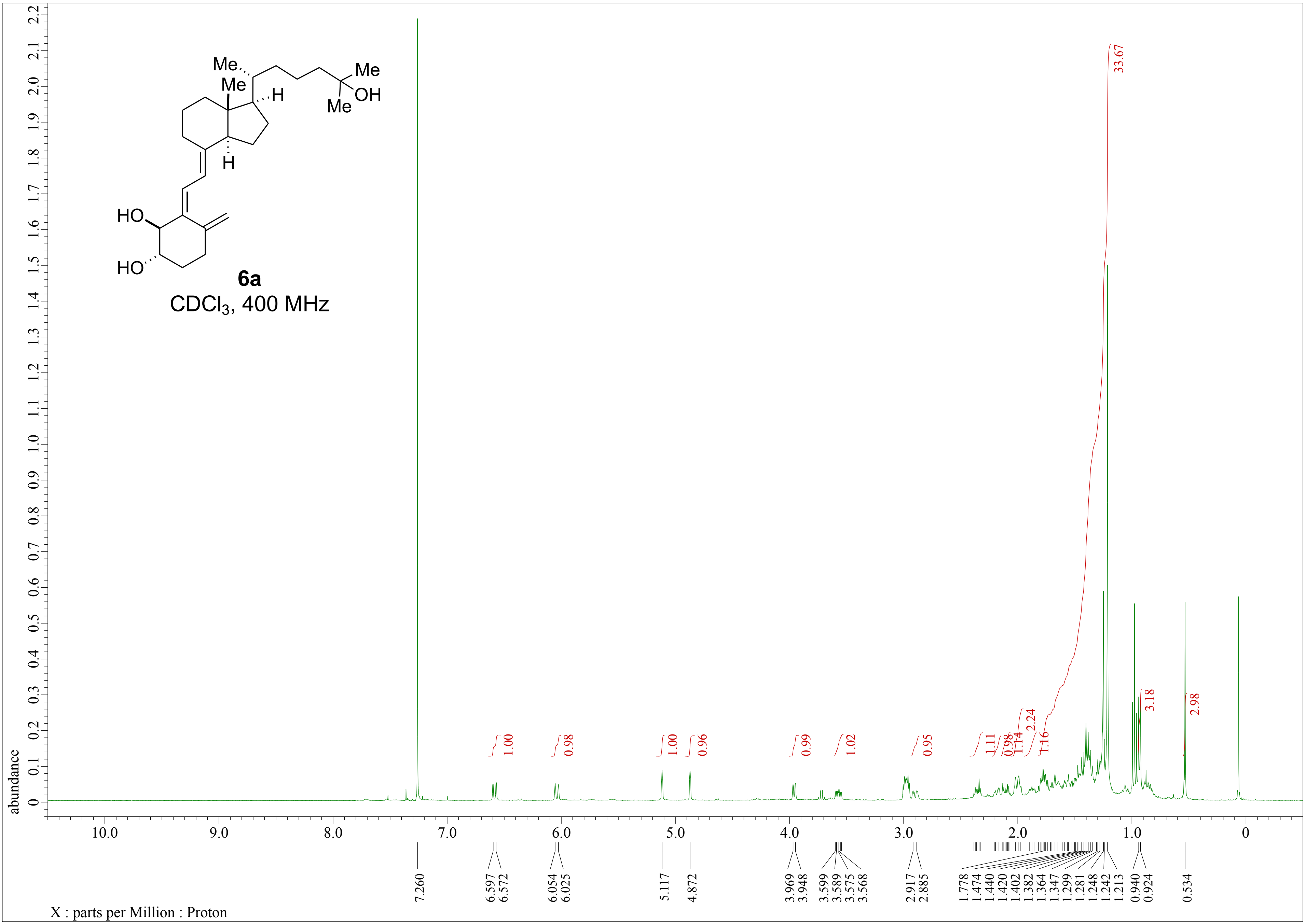
6a
CDCl3, 400 MHz
abundance
X : parts per Million : Proton
7.260
6.597
6.572
6.054
6.025
5.117
4.872
3.969
3.948
3.599
3.589
3.575
3.568
2.917
2.885
1.778
1.474
1.440
1.420
1.402
1.382
1.364
1.347
1.299
1.281
1.248
1.242
1.213
0.940
0.924
0.534
1.00
0.98
1.00
0.96
0.99
1.02
0.95
1.11
0.98
1.14
2.24
1.16
33.67
3.18
2.98

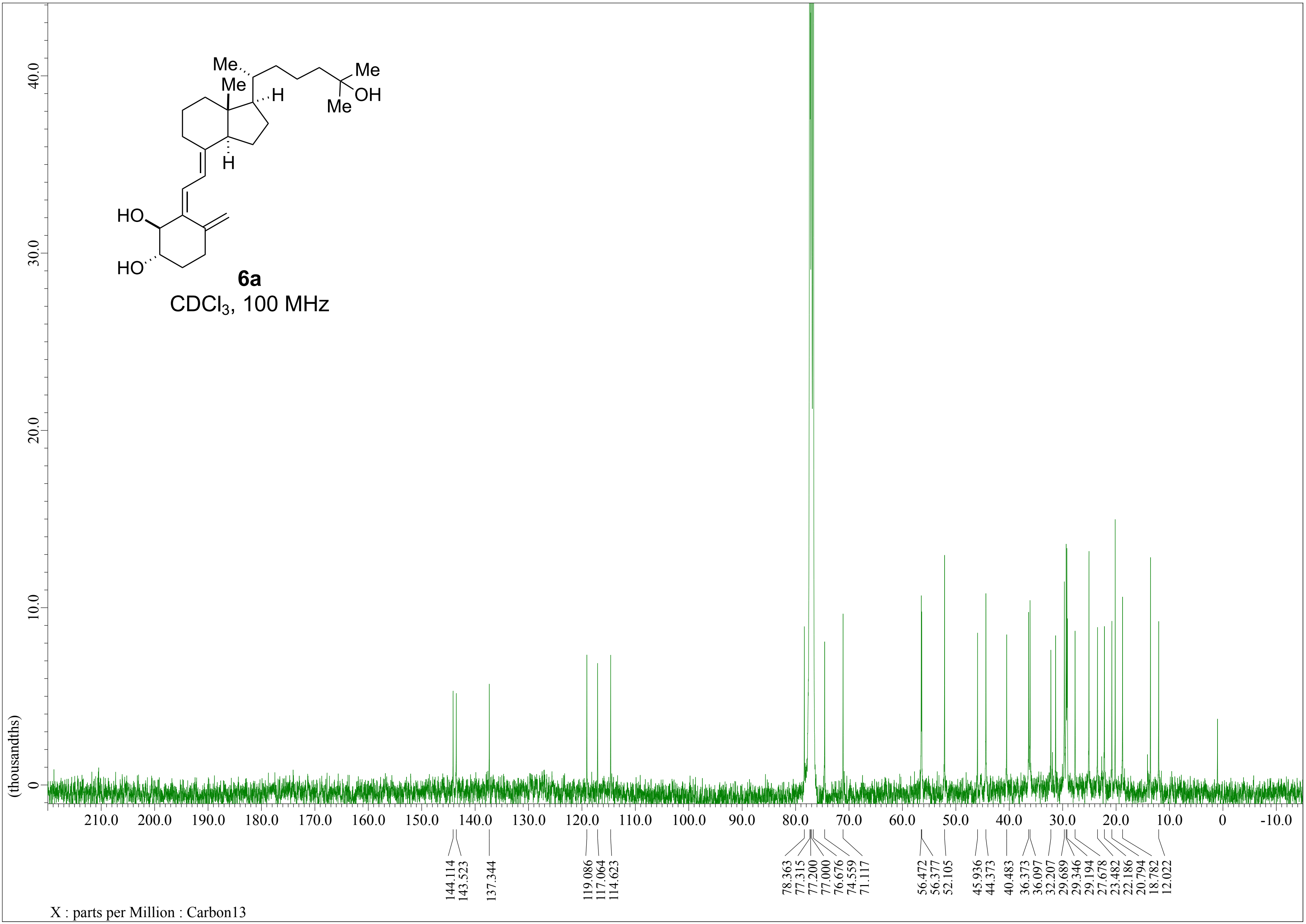

Me
Me
Me
OH
Me
H
H
HO
HO
6a
CDCl$_3$, 100 MHz
40.0
30.0
20.0
10.0
0
(thousandths)
210.0 200.0 190.0 180.0 170.0 160.0 150.0 140.0 130.0 120.0 110.0 100.0 90.0 80.0 70.0 60.0 50.0 40.0 30.0 20.0 10.0 0 -10.0
144.114
143.523
137.344
119.086
117.064
114.623
78.363
77.315
77.200
77.000
76.676
74.559
71.117
56.472
56.377
52.105
45.936
44.373
40.483
36.373
36.097
32.207
29.689
29.346
29.194
27.678
23.482
22.186
20.794
18.782
12.022
X : parts per Million : Carbon13

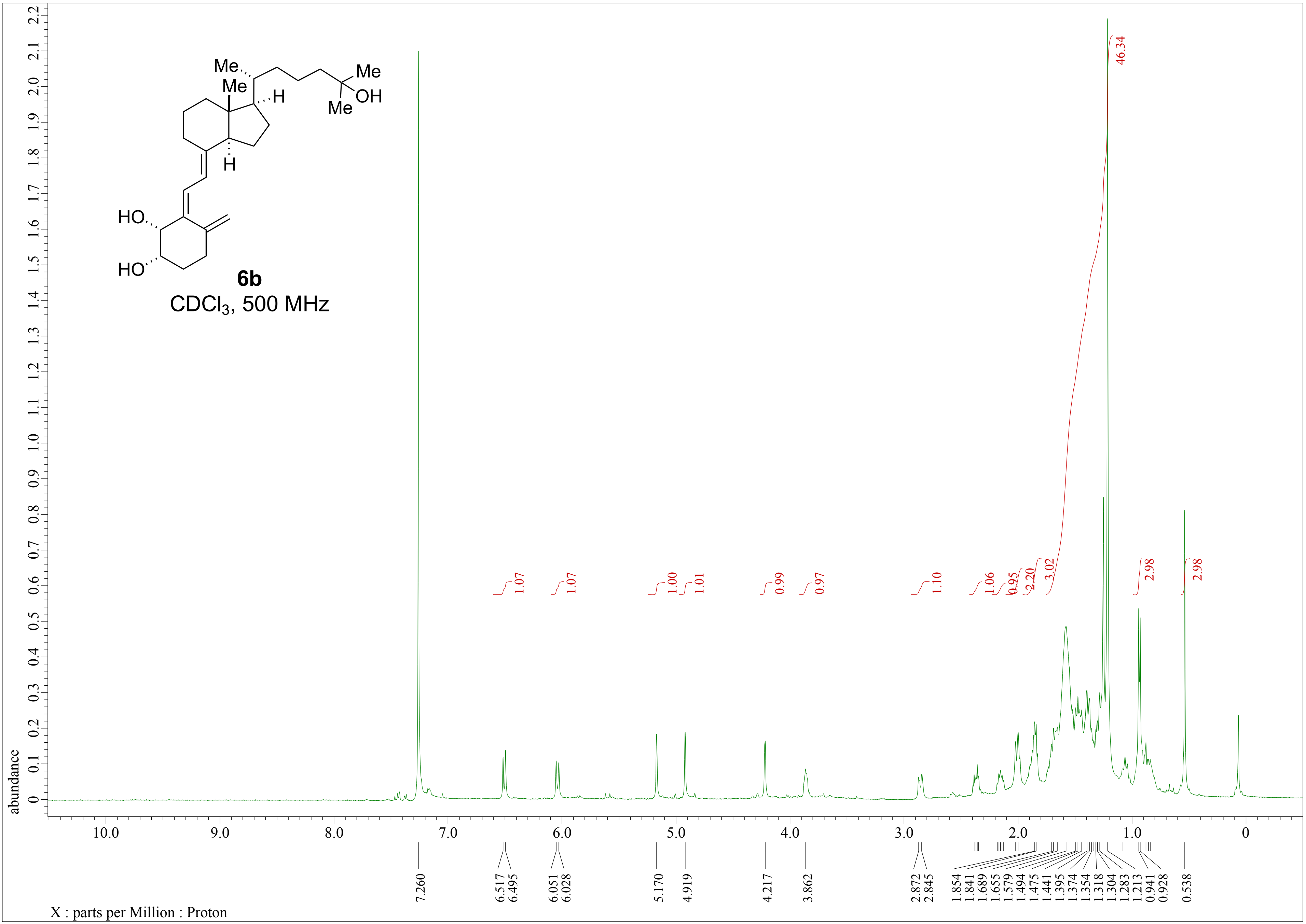

6b
CDCl3, 500 MHz
Me
Me
H
Me
OH
Me
H
HO
HO
abundance
X : parts per Million : Proton
7.260
6.517
6.495
6.051
6.028
5.170
4.919
4.217
3.862
2.872
2.845
1.854
1.841
1.689
1.655
1.579
1.494
1.475
1.441
1.395
1.374
1.354
1.318
1.304
1.283
1.213
0.941
0.928
0.538
1.07
1.07
1.00
1.01
0.99
0.97
1.10
1.06
0.95
2.20
3.02
46.34
2.98
2.98

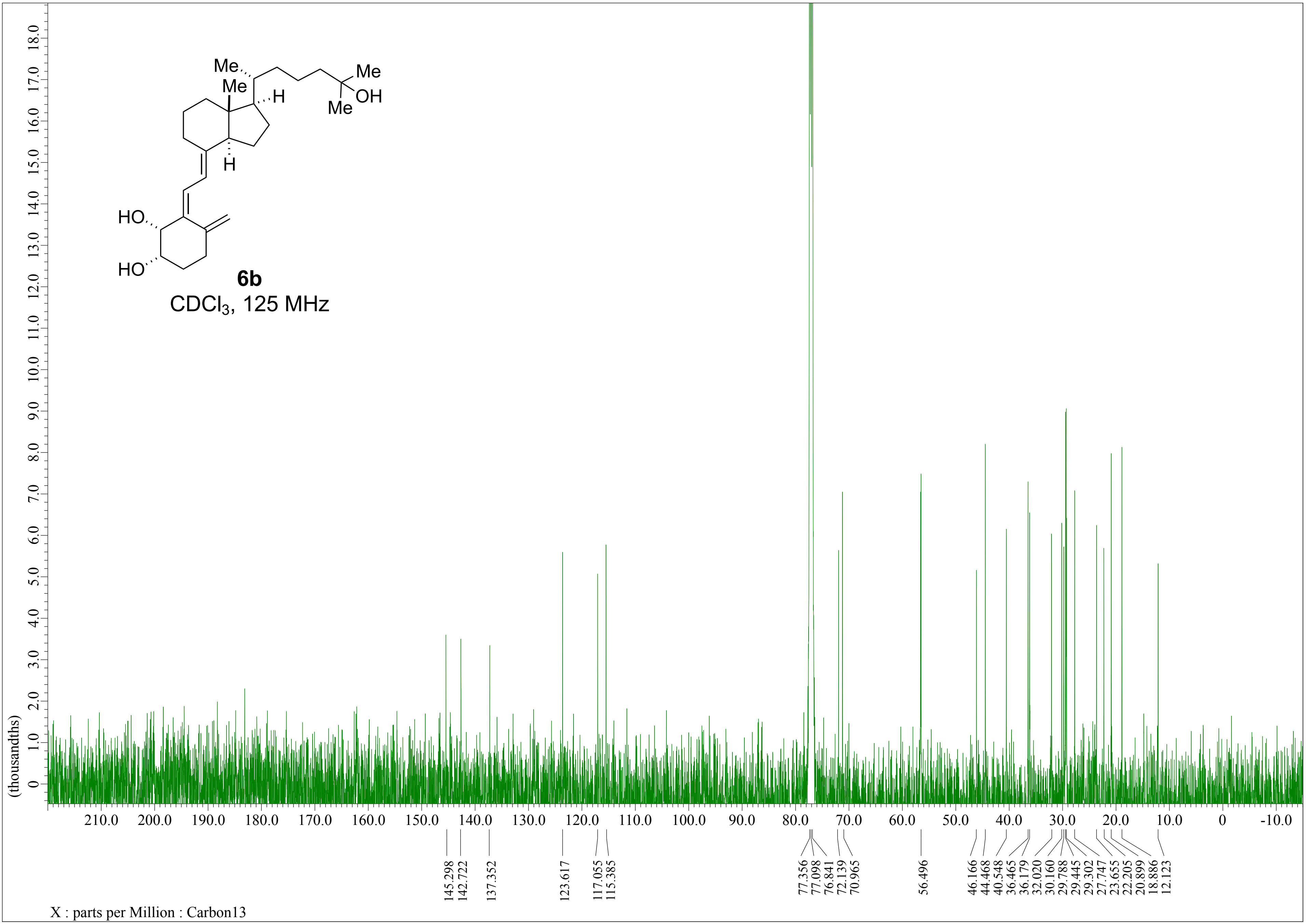
Me
Me
H
Me
OH
Me
H
HO
HO
6b
CDCl3, 125 MHz
(thousandths)
X : parts per Million : Carbon13
210.0 200.0 190.0 180.0 170.0 160.0 150.0 140.0 130.0 120.0 110.0 100.0 90.0 80.0 70.0 60.0 50.0 40.0 30.0 20.0 10.0 0 -10.0
145.298
142.722
137.352
123.617
117.055
115.385
77.356
77.098
76.841
72.139
70.965
56.496
46.166
44.468
40.548
36.465
36.179
32.020
30.160
29.788
29.445
29.302
27.747
23.655
22.205
20.899
18.886
12.123

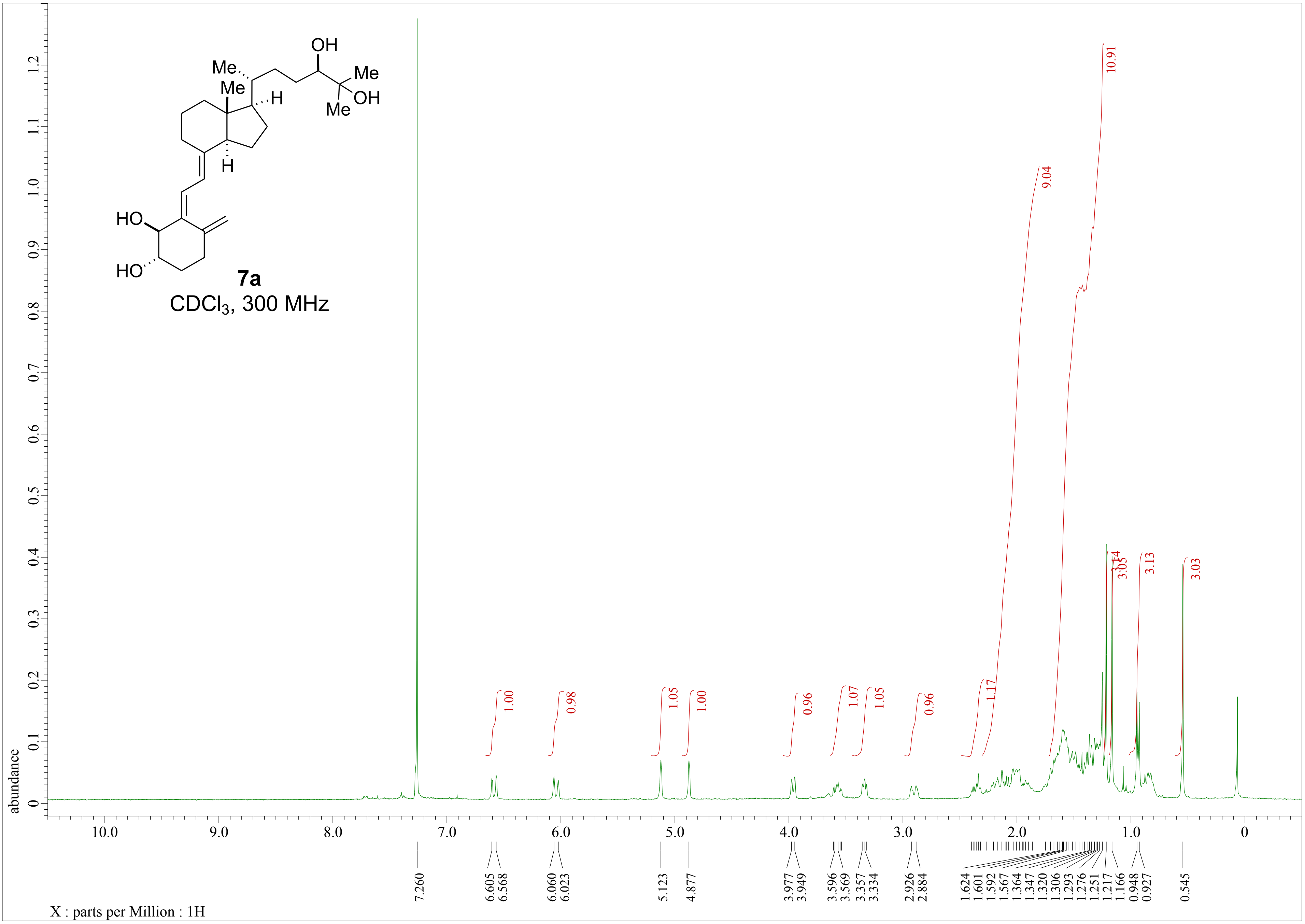

OH
Me
Me
Me
H
Me
OH
H
HO
HO
7a
CDCl3, 300 MHz
abundance
X : parts per Million : 1H
1.00
0.98
1.05
1.00
0.96
1.07
1.05
0.96
1.17
9.04
10.91
3.14
3.05
3.13
3.03
7.260
6.605
6.568
6.060
6.023
5.123
4.877
3.977
3.949
3.596
3.569
3.357
3.334
2.926
2.884
1.624
1.601
1.592
1.567
1.364
1.347
1.320
1.306
1.293
1.276
1.251
1.217
1.166
0.948
0.927
0.545

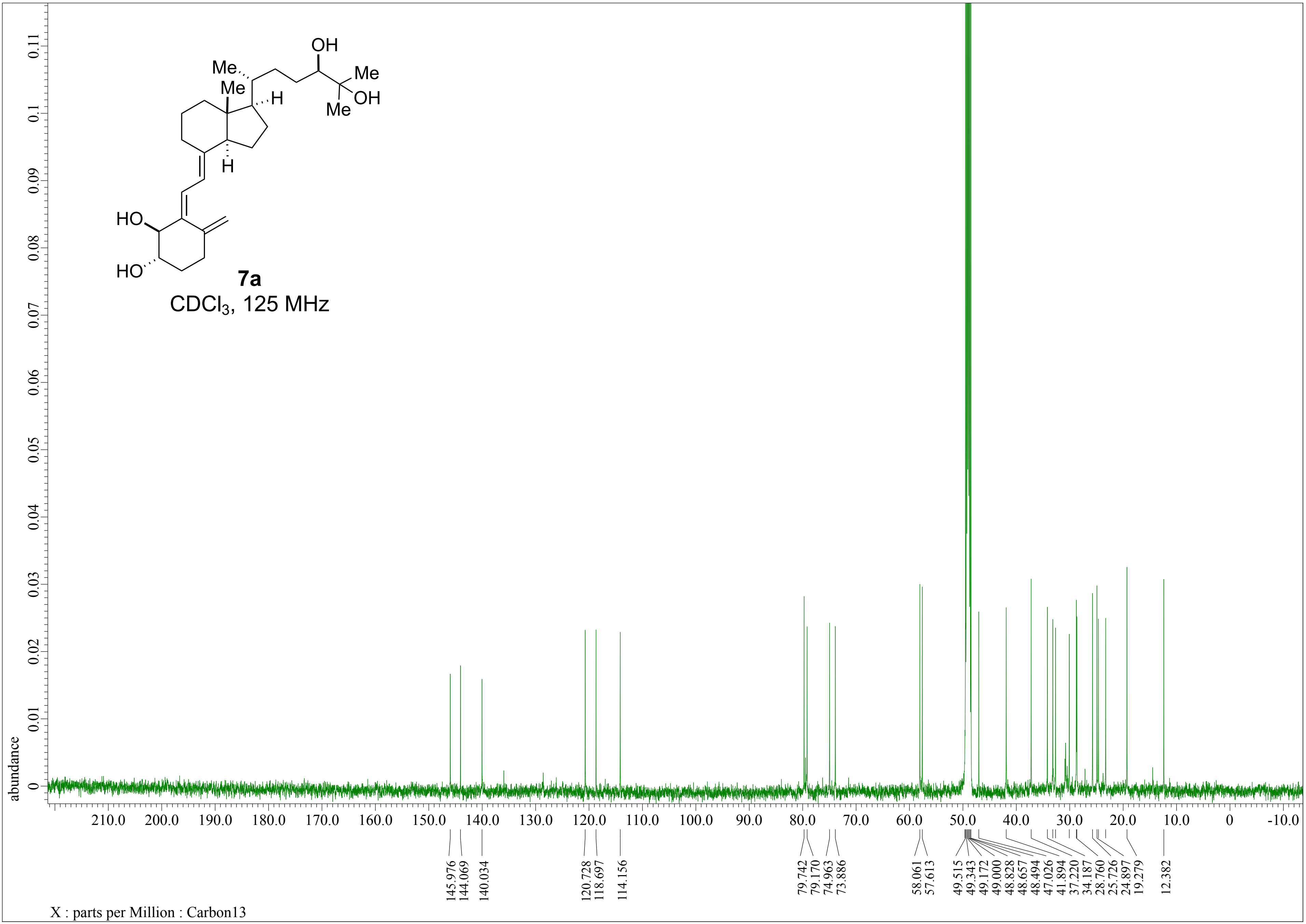
7a
CDCl3, 125 MHz
145.976
144.069
140.034
120.728
118.697
114.156
79.742
79.170
74.963
73.886
58.061
57.613
49.515
49.343
49.172
49.000
48.828
48.657
48.494
47.026
41.894
37.220
34.187
28.760
25.726
24.897
19.279
12.382
abundance
X : parts per Million : Carbon13

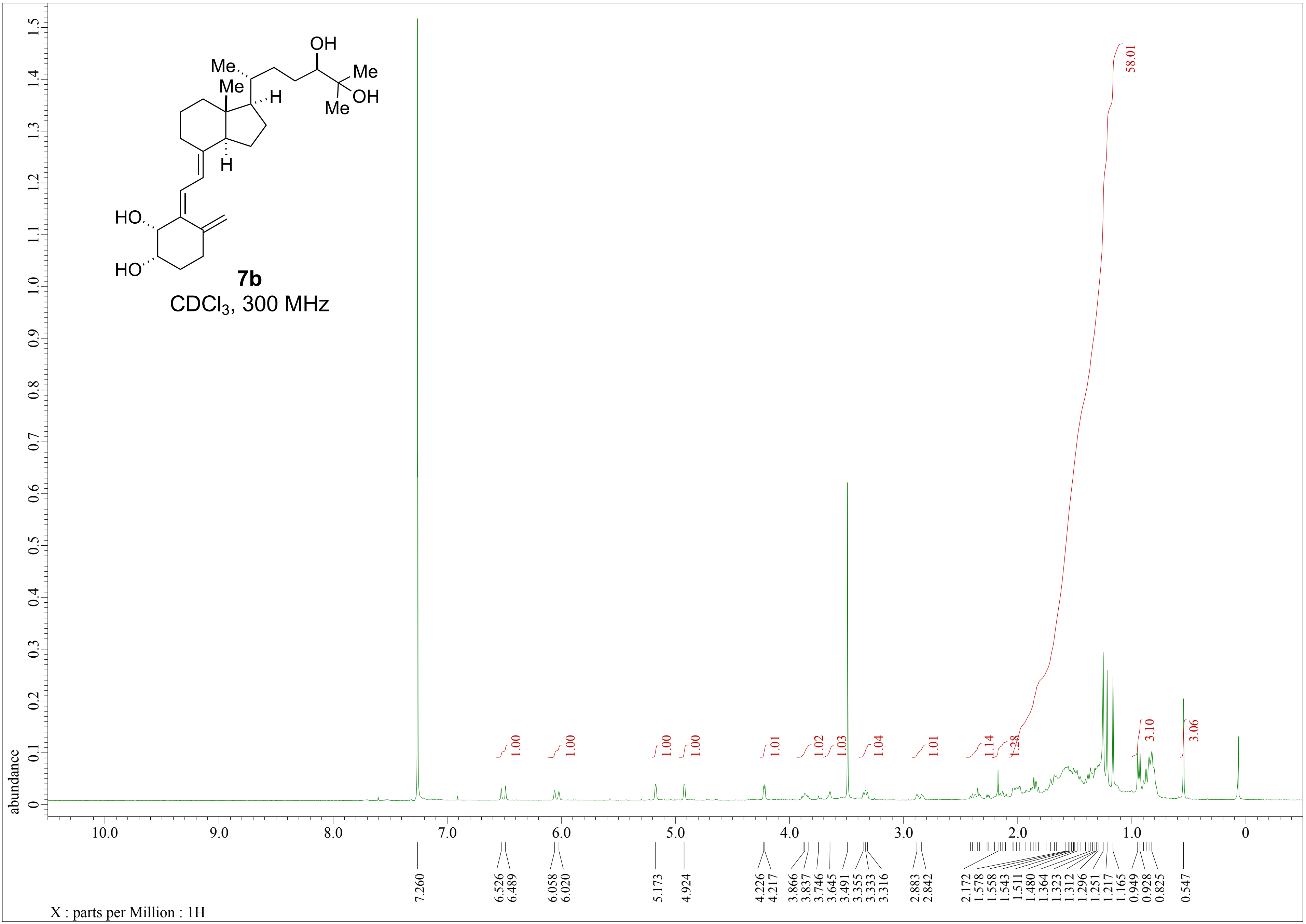
OH
Me
Me
Me
H
Me
OH
Me
H
HO
HO
7b
CDCl3, 300 MHz
abundance
X : parts per Million : 1H
1.00
1.00
1.00
1.00
1.01
1.02
1.03
1.04
1.01
1.14
1.28
58.01
3.10
3.06
7.260
6.526
6.489
6.058
6.020
5.173
4.924
4.226
4.217
3.866
3.837
3.746
3.645
3.491
3.355
3.333
3.316
2.883
2.842
2.172
1.578
1.558
1.543
1.511
1.480
1.364
1.323
1.312
1.296
1.251
1.217
1.165
0.949
0.928
0.825
0.547

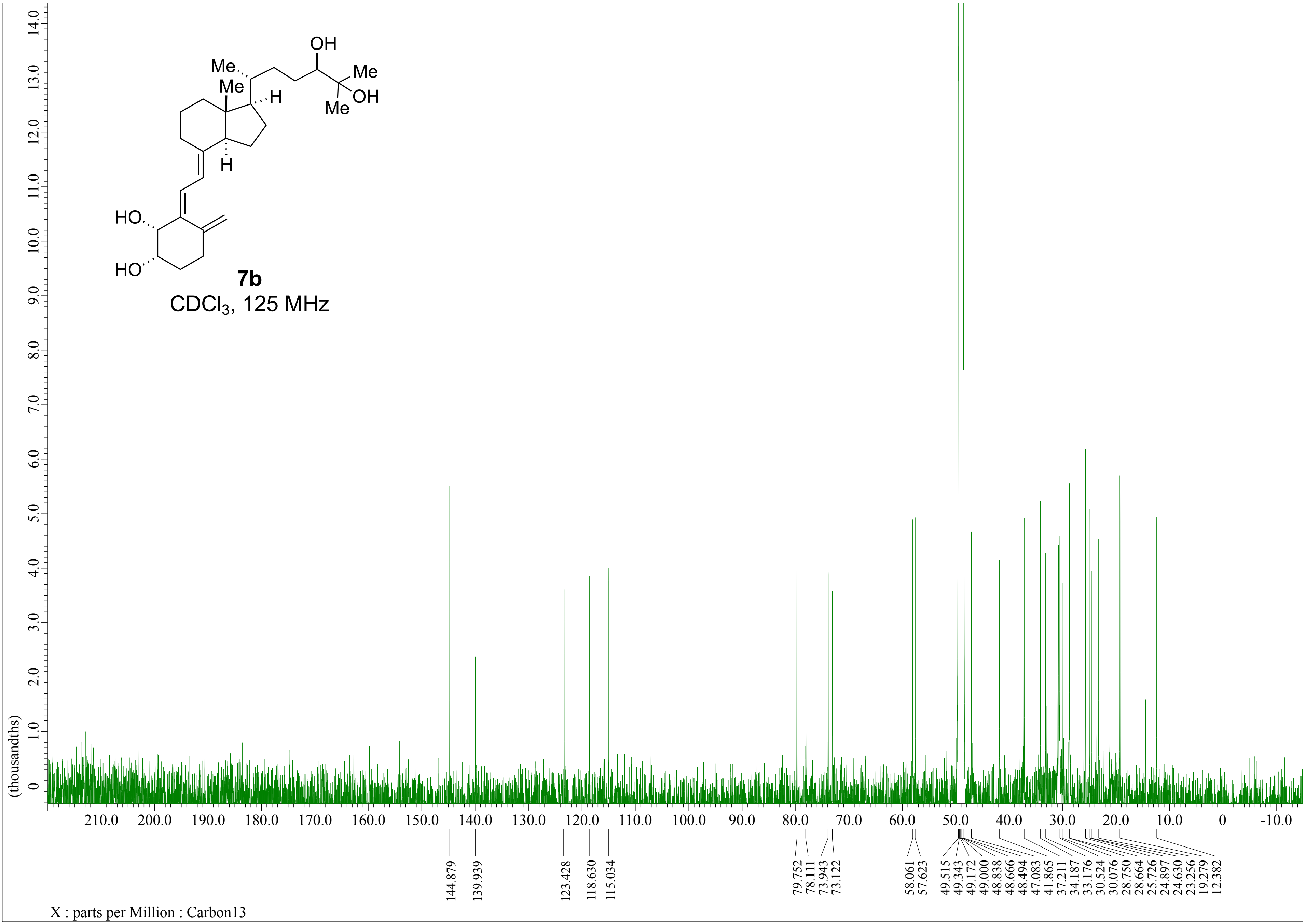
OH
Me
Me
Me
H
Me
OH
H
HO
HO
7b
$CDCl_3$, 125 MHz
(thousandths)
144.879
139.939
123.428
118.630
115.034
79.752
78.111
73.943
73.122
58.061
57.623
49.515
49.343
49.172
49.000
48.838
48.666
48.494
47.083
41.865
37.211
34.187
33.176
30.524
30.076
28.750
28.664
25.726
24.897
24.630
23.256
19.279
12.382
X : parts per Million : Carbon13
